# Effectiveness of virtual reality-based rehabilitation on quality of life and upper extremity functions among stroke survivors: A systematic review and meta-analysis

**DOI:** 10.1016/j.ijnsa.2026.100629

**Published:** 2026-07-12

**Authors:** Ranee Reesa Tan, Sok Ying Liaw, Yasmine Yan Qi Thng, Suebsarn Ruksakulpiwat, Ladislav Batalik, Mei Sin Chong

**Affiliations:** a Alexandra Hospital, 378 Alexandra Road, 159964, Singapore; b Alice Lee Centre for Nursing Studies, Yong Loo Lin School of Medicine, Centre for Translational Medicine, Block MD6, Level 5, 14 Medical Drive, 117599, Singapore; c Faculty of Nursing, Mahidol University, 2 Wang Lang Road, Siriraj, Bangkoknoi, Bangkok, 10700, Thailand; d Department of Rehabilitation, University Hospital Brno, Jihlavska 20, 62500, Brno, Czech Republic; e Department of Physiotherapy and Rehabilitation, Faculty of Medicine, Masaryk University, Kamenice 753/5, 62500, Brno, Czech Republic

**Keywords:** Virtual reality, Rehabilitation, Upper extremity, Quality of life, Stroke, Feasibility, Exergames

## Abstract

**Background:**

Stroke rehabilitation faces challenges such as limited accessibility and engagement, prompting interest in virtual reality-based interventions. While virtual reality shows promise, its effectiveness in improving quality of life (QoL) and upper extremity (UE) functions in stroke survivors remains unclear.

**Objectives:**

To evaluate (1) the effectiveness of virtual reality-based interventions in QoL and UE functions among stroke survivors and (2) the feasibility and safety indicators across trials.

**Data sources:**

A comprehensive search was conducted across six electronic databases: PubMed, EMBASE, Cochrane, Scopus, PsycINFO, and CINAHL to identify relevant articles published between Jan 2014 and December 2024. The search was updated on 9 May 2026.

**Review methods:**

The Cochrane Handbook for Systematic Reviews of Interventions and the Preferred Reporting Items for Systematic Reviews and Meta-Analyses (PRISMA) guidelines were employed to ensure methodological rigor and standardized reporting. Two independent reviewers assessed the eligibility of the articles, and the quality of the studies were appraised using the Revised Cochrane Risk of Bias Assessment Tool for Randomized Trials (RoB2). Feasibility indicators (participation, adherence, attrition) and adverse events were also extracted.

**Results:**

Thirty-four randomized controlled trials involving 1834 participants were included. Two studies had a low risk of bias, 19 had some concerns, and 13 had a high risk. Meta-analyses revealed non-significant effects of virtual reality-based interventions on QoL (SMD = –0.09, 95% CI –0.30 to 0.11, *p* = .36; low certainty) and UE functions assessed by upper extremity Fugl-Meyer (SMD = 0.56, 95% CI -0.02 to 1.15, *p* = .06; very low certainty) and Action Research Arm Test (SMD = 0.15, 95% CI -0.22 to 0,52, *p* = .41; low certainty). Narrative synthesis yielded mixed findings. Feasibility outcomes were generally favorable: participation among eligible patients was typically >80%, attrition <20%, and adherence exceeded 80% in the few trials reporting it. Adverse events were mainly mild and comparable to those in control groups.

**Conclusion:**

Current evidence does not demonstrate the superiority of virtual reality-based interventions over conventional rehabilitation for improving QoL or UE functions in stroke survivors, despite generally favorable feasibility and safety profiles. The low-to-very low certainty of evidence highlights the need for stronger methodological rigor, theoretical frameworks, and long-term follow-up. Virtual reality may serve as a complementary option when access to traditional rehabilitation is limited.

**Registration:**

PROSPERO (CRD42024619427).

**What is already known:**

• Virtual reality rehabilitation interventions which are game-based, are increasingly adopted and generally considered feasible and safe, but existing systematic reviews have reported mixed results regarding their efficacy.
• Conventional post-stroke rehabilitation is often limited by challenges related to access and adherence.

Alt-text: Unlabelled box dummy alt text

**What this paper adds:**

• This review found that virtual reality-based rehabilitation did not significantly improve quality of life and upper extremity functions compared to usual care or no intervention.
• This review also confirms the favorable feasibility of such interventions, demonstrating high participation (> 80%) and adherence (> 80%), low attrition (< 20%), and a mild adverse event profile comparable to control groups.

Alt-text: Unlabelled box dummy alt text

## 1 Introduction

Globally, the prevalence of stroke has escalated to over 100 million people, with an annual incidence of 12.2 million new strokes (World Stroke Organization, 2026). This trend is expected to worsen, driven by the rising prevalence of chronic diseases and an aging population (Béjot and Yaffe, 2019; Yousufuddin and Young, 2019). Stroke imposes substantial economic burdens on healthcare systems globally, particularly in post-stroke care. For instance, the highest costs of post-stroke care in outpatient settings were reported in the US, with a mean expenditure of $1236 per patient per month, followed by the UK at $1039 (Rajsic et al., 2019). Furthermore, post-stroke depression is a prevalent complication, affecting between 18% and 33% of stroke survivors, and is associated with poor patient outcomes (Medeiros et al., 2020). Approximately 50 and 75% of stroke survivors experience persistent motor impairment in their affected upper extremities (Anwer et al., 2022). As a result, many face challenges in performing activities of daily living. Wondergem et al. (2017) found that up to 40% of stroke survivors experience a deterioration in their ability to carry out daily tasks over time, often leading to functional dependence. The concomitant reduction in activities of daily living and the presence of post-stroke depression may reduce quality of life (QoL) (Li et al., 2023).

Given the complex nature and impacts of stroke, the needs of stroke survivors are often unique (Hawkins et al., 2017). Stroke rehabilitation initiated after 24 h of a stroke is deemed safe (Coleman et al., 2017). According to Vazquez-Guimaraens et al. (2021), most studies indicate that neurological recovery predominantly occurs within the first three months, with optimal recovery observed in the initial four to six weeks. Neuroplasticity, the brain’s ability to adapt and reorganize neural networks through repetitive experiences (Puderbaugh and Emmady, 2023), is vital for regaining motor and cognitive functions after a stroke (Aderinto et al., 2023). Conventional rehabilitation typically includes physiotherapy (PT), occupational therapy (OT), and speech therapy (ST), tailored to the nature and severity of stroke impairment (Nair & Taly, 2002). Outpatient stroke therapy sessions typically involved patients attending two to three days per week, with each day consisting of a single session that averaged 36 ± 14 min in duration (Lang et al., 2009). Despite the critical importance of stroke rehabilitation, participation and adherence rates have consistently been challenging (Miller et al., 2016). Conventional rehabilitation frequently presents significant challenges, including limited accessibility due to geographical disparities and the absence of individualized rehabilitation plans, which can hinder optimal patient outcomes (Li et al., 2024).

To overcome the challenges associated with conventional stroke rehabilitation, technological rehabilitation programs have been increasingly adopted. The programs not only accommodate the mobility limitations of stroke patients but also facilitate home-based rehabilitation, thereby minimizing inconvenience. Among these technologies, two related but distinct approaches have gained prominence including game-based rehabilitation and virtual reality (VR)-based rehabilitation. Game-based rehabilitation refers to interventions that incorporate game-like elements such as meaningful play, goals, feedback, rewards, or challenges to enhance patient engagement and motivation during therapy (Barrett et al., 2016). VR-based rehabilitation uses computer-generated environments to simulate real-world or imaginary scenarios for therapeutic purposes (Laver et al., 2011). Importantly, these two concepts are not mutually exclusion, with many VR-based interventions incorporate game-like features (Lohse et al., 2014), and game-based interventions can be delivered using VR. For the purposes of this review, both VR-based and game-based interventions are considered, recognizing their shared therapeutic goals.

The feasibility and effectiveness of game-based intervention protocols for stroke rehabilitation have been well-documented, offering engaging experiences (Tosto-Mancuso et al., 2022). Additionally, devices deployed in game-based interventions provide significant flexibility and adaptability to meet individual patient needs (Baranyi, 2023). Systematic reviews have been conducted on game-based rehabilitation for post-stroke patients. Saeedi et al. (2021) focused on physical rehabilitation, identifying key components including balancing training, limb mobilization, and muscle strengthening. This review reported that VR-based approaches and Nintendo Wii Fit games were most used, with a particular focus on limb movement and balance training. A more recent systematic review by Sanchez-Gil et al. (2025) examined the devices used in game-based rehabilitation and their impact on emotional, personal, and social aspects of stroke survivors. The authors highlighted that gamified devices improved motor and cognitive function and had a significant positive impact on emotional, social and personal levels of stroke survivors. In contrast to these broad systematic reviews, existing meta-analyses have primarily focused on game-based rehabilitation for the upper extremities post-stroke (Karamians et al., 2020; Wang et al., 2022). Across the 38 studies included in the review by Karamians et al. (2020), participants in the VR or gaming intervention groups achieved a mean improvement of 28.5% of the maximum possible score on the outcome measures (Wolf Motor Functioning Test, the Fugl-Meyer, or the Action Research Arm Test). Wang et al. (2022) included only non-immersive device rehabilitation modes and reported that these interventions significantly improved upper limb motor function and hand dexterity compared to conventional therapy.

With the rapid advancement of technologies, VR has emerged as a promising tool within game-based rehabilitation programs, enabling patients to engage with simulated environments that resemble real-life scenarios and facilitating interactive therapeutic experiences. VR devices can be classified by the level of immersion experienced by the user: 1) non-immersive VR, which uses standard computers or gaming consoles (e.g., mouse, keyboard, joystick), allowing users to experience virtual environment while fully aware of their real surroundings; 2) semi-immersive VR involves projecting the virtual environment onto large screens and employs advanced devices (e.g., cybergloves, motion sensors), offering partial immersion but not complete separation from the real world; and 3) immersive VR uses a head-mounted display and a 3D input device, allowing users to fully engaged with the virtual environment while remaining disconnected with the real world (Salatino et al., 2023).

Despite this promising evidence, it remains unclear which specific characteristics of virtual reality rehabilitation most significantly affect QoL and motor functions in stroke survivors. Therefore, this present review aimed to evaluate the effectiveness of VR rehabilitation on QoL and UE functions among stroke survivors undergoing rehabilitation. In addition, we aimed to describe the feasibility and safety indicators across trials.

## 2 Methods

This review was conducted in accordance with the Cochrane Handbook for Systematic Reviews of Interventions and the Preferred Reporting Items for Systematic reviews and Meta-Analyses (PRISMA) guidelines (Page et al., 2021) (Supplementary Material: Table S1). The study protocol has been registered in the International Prospective Register of Systematic Reviews (PROSPERO) (CRD42024619427).

### 2.1 Eligibility criteria

#### 2.1.1 Inclusion criteria

Randomized controlled trials (RCTs) utilizing VR-based interventions aimed at improving QoL and motor functions were deemed eligible for inclusion in this review. The review was guided by a PICO (population, intervention, comparison, outcome, study design) search strategy, which was employed to formulate review questions and direct comprehensive literature searches.

##### 2.1.1.1 Population

Patients who were clinically diagnosed with stroke (ischemic or hemorrhagic), aged 18 years and over, able to provide informed consent, and who understood instructions were included. Exclusion criteria include participants with post-stroke complications such as visual or auditory impairment, vestibular dysfunction, aphasia, or cognitive disabilities due to other neurological conditions.

##### 2.1.1.2 Intervention

Studies with any form of VR-based interventions were considered, regardless of delivery mode, and whether provided as a standalone or in combination with conventional rehabilitation.

##### 2.1.1.3 Comparison

Studies with any type of control, such as no intervention or conventional rehabilitation (usual care), were included.

##### 2.1.1.4 Outcomes

The primary outcome included QoL with any use of generic or stroke-specific QoL assessment tools. Meanwhile, the secondary outcome was the upper extremity (UE) functions. Feasibility and safety indicators, including participants rates, adherence, attrition, and adverse events were extracted as descriptive outcomes of interest to contextualize the primary and secondary outcomes.

### 2.2 Search strategy

A comprehensive search was conducted across six electronic databases (PubMed, EMBASE, Cochrane, Scopus, PsycINFO, CINAHL) to retrieve relevant published literature. The ProQuest Theses and Dissertations and Google Scholar were employed to access unpublished and grey literature. The search utilized a combination of keywords and MeSH terms, including ‘virtual reality’, ‘exergaming’, ‘gamification’, ‘stroke’, and ‘quality of life’, along with Boolean operators such as ‘AND’ and ‘OR’ as appropriate. The search was conducted for studies published between January 2014 and December 2024. This 10-year limit was applied due to the rapid pace of technological advancement, ensuring the review captured current and relevant interventions (Xu et al., 2021). The search was updated on 9 May 2026. Only English-language articles were considered, and a librarian was consulted to optimize the search strategies. Further details on the search strategies can be found in Supplementary Material: Table S2.

### 2.3 Selection process

The retrieved articles were imported into the EndNote 21 software for organization and management, with duplicate articles removed using the functionality of the software. Two reviewers (R.T.R. and Y.T.) independently screened the titles and abstracts against the predetermined eligibility criteria to identify studies for full-text review. Any disagreements were resolved by consulting a third reviewer (C.M.S).

### 2.4 Data extraction

Data extraction was independently conducted by two reviewers (R.T.R and C.M.S) using a custom-developed data extraction form, adapted from the Cochrane Data collection Form for Randomized Controlled Trials (Higgins et al., 2024). The extracted information encompassed the following details: authors, publication year, country of study, sample size, participant characteristics, stroke (ischemic or hemorrhagic), specifics of the intervention and control groups, outcome measurement tools, results, and attrition rate. In addition, we extracted indicators of trial feasibility and safety, including the numbers of participants screened, eligible, randomized and analyzed, attrition rates, adherence or engagement with the intervention and control conditions where reported, and adverse events (type and severity). These indicators were not predefined as formal outcomes for meta-analysis. Rather, they were extracted as descriptive outcomes of interest, informing the interpretation of the effectiveness data and providing practical guidance for stake holders of VR-based interventions in stroke rehabilitation settings.

### 2.5 Quality assessment and certainty of evidence

Quality appraisals were independently conducted by two reviewers (R.T.R. and Y.T.) using the revised Cochrane Risk of Bias for Randomized Trials tool (RoB 2), which assesses five domains: 1) bias arising from the randomization process, 2) bias due to deviations from intended interventions, 3) bias due to missing outcome data, 4) bias in measurement of the outcome, and 5) bias in selection of the reported result (Sterne et al., 2019). The overall risk-of-bias judgements were categorized as having low, some concerns, or high risk of bias. The Grading of Recommendations, Assessment, Development, and Evaluation (GRADE) framework was employed to determine the confidence in the quality of evidence of each outcome, considering factors such as study design, risk of bias, inconsistency, indirectness, and imprecision, and was rated as high, moderate, low or very low (Guyatt et al., 2011). The GRADEpro software was utilized to create a summary of the evidence table (GRADEpro GDT, 2025). Any disagreements were discussed with the third reviewer (C.M.S.).

### 2.6 Data synthesis and statistical analyses

Meta-analyses were performed using the Review Manager version 8.13.0. (RevMan, 2024). With a combination of results from two or more separate studies, the inverse-variance random effects model was used to analyze pooled effect size for studies, as it provides a better fit for actual sampling distribution (Borenstein et al., 2010). Studies with data presented as median and interquartile range (IQR) were converted to means and standard deviations (SDs) using formulas from Shi et al. (2020), Luo et al. (2018), and Wan et al. (2014). Mean difference (MD) was used for outcomes measured on the same scale, while standardized mean difference (SMD) was used for outcomes measured on different scales. The effect size was interpreted using the Cohen’s *d* effect size, categorized as small (*d* < 0.4), moderate (0.4 ≤ d < 0.8) and large (d ≥ 0.8) (Schünemann et al., 2019). Heterogeneity was assessed using Chi-square ($\chi^{2}$) and $I^{2}$ statistics, with a significance level of *p* < 0.10 (Higgins et al., 2024). The $I^{2}$ value was interpreted as indicating low heterogeneity (≤ 30%), moderate heterogeneity (31–50%), substantial heterogeneity (51–75%), and considerable heterogeneity (> 75%) (Higgins et al., 2024).

Sensitivity analysis, a process to evaluate the robustness of the findings, was conducted for analysis with substantial or considerable heterogeneity, by substituting alternative decisions or value ranges for those that were unclear (Higgins et al., 2024). Subgroup analyses were conducted to assess whether variations in QoL were influenced by different game elements and to explore potential causes of heterogeneity (Higgins et al., 2024). When statistical pooling for meta-analysis was not feasible, a narrative synthesis or synthesis without meta-analysis (SwiM) approach was employed to qualitatively describe the reported data from the studies (Campbell et al., 2020).

## 3 Results

### 3.1 Study selection

**Fig. 1:**
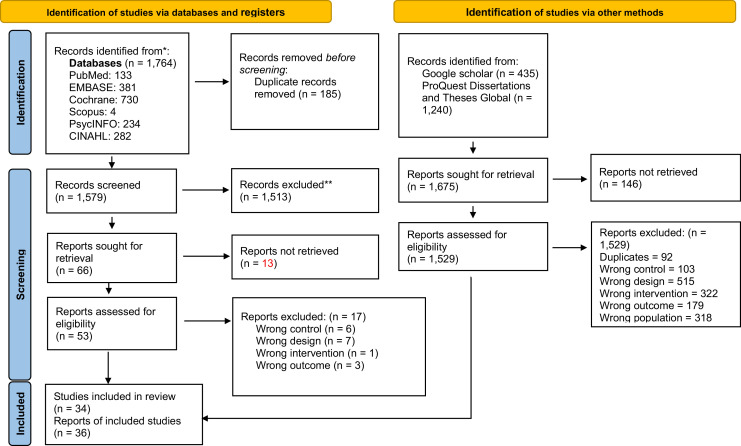
PRISMA Flow diagram.

A total of 1764 articles were retrieved from the six electronic bibliographic databases. The detailed study screening and selection process is illustrated in the PRISMA flow diagram (Fig. 1). After the removal of duplicates (n = 185), 1579 articles were excluded following the screening of their titles and abstracts, resulting in 66 articles for retrieval. Of these, 13 articles could not be retrieved due to unavailability of the full text. A total of 53 articles underwent full-text screening. An additional 1675 articles were identified through grey literature. Ultimately, 34 randomized controlled trials (36 reports) were included in this review. Supplementary Material: Table S3 (Supplementary Material) presents a comprehensive list of studies excluded during the full-text screening phase, along with specific exclusion reasons.

### 3.2 Characteristics of included studies

Table 1 summarizes the characteristics of the studies included in this review. A total of 1834 participants were involved in 34 RCTs (36 reports), with 928 adults in the intervention group and 906 in the control group. The studies were conducted across various countries: Brazil (n = 1), Canada (n = 1), China (n = 1), Czech Republic (n = 1), France (n = 3), Hong Kong (n = 1), India (n = 2), Indonesia (n = 1), Netherlands (n = 1), Pakistan (n = 2), Portugal (n = 1), Spain (n = 3), South Korea (n = 9), Switzerland (n = 1), Taiwan (n = 2), Turkey (n = 3), and the United Kingdom (n = 1). Sample sizes varied, ranging from as small as 10 participants (Song and Lee, 2021) to as large as 235 participants (Adie et al., 2017). The intervention duration ranged between two weeks (Ali et al., 2024) to 12 weeks (Huber et al., 2025; Kim, 2018). Attrition rates across studies ranged between 0% (Amin et al., 2024; Choi et al., 2014; da Silva Ribeiro et al., 2015; Faria et al., 2016; Kim, 2018; Latif et al., 2025; Marques-Sule et al., 2021; Park et al., 2021; Velmurugan et al., 2023) to 50% (Shin et al., 2016). Control groups in all included studies received usual care, which typically comprised standard exercise therapy or conventional therapy (i.e., physiotherapy and occupational therapy). In addition to usual rehabilitation, one study had participant-tailored arm exercises (Adie et al., 2017), Ali et al. (2024) provided upper limb task-based training. One study with active control with a motor relearning program (Velmurugan et al., 2023).

**Table 1:** Summary of the included studies (N = 34).

Author (year) Country	Study design	Participants a) Sample size b) Mean age c) Female d) Type of stroke e) Phase of stroke (acute/subacute/ chronic)	Study setting/ Provider	Intervention a) setting b) intervention provider c) description of the intervention d) dose e) other information	Control a) setting b) description of the intervention c) dose d) other information	Data collection time points Outcome(s)/measurement tools	a) Findings b) Attrition
Adie et al. (2017) United Kingdom	Multicenter, pragmatic, parallel group, RCT	a) N = 235 IG = 117, CG = 118 b) Mean age = 67.3 ± 13.4 IG: mean age = 66.8 ± 14.6 CG: mean age = 68.0 ± 11.9 c) IG: Female = 51 CG: Female = 53 d) IG: ischemic stroke = 104, hemorrhagic stroke = 13 CG: ischemic stroke = 105, hemorrhagic stroke = 13 e) Acute/ subacute stroke survivors	Home-based/ Research therapist	a) Home-based b) A research therapist c) Warm-up exercise for 15 min, followed by intervention. Wii sports games (bowling, tennis, golf and baseball). d) Dose: 1) Amount = 45 min 2) Frequency = daily 3) Duration = 6 weeks e) Daily diary, weekly telephone follow-up, usual rehabilitation therapy by local stroke team.	a) Home-based b) A research therapist c) Warm-up exercise for 15 min, followed by tailored arm exercises. Participant-tailored arm exercises (based on the Graded Repetitive Arm Supplementary Program). d) Dose: 1) Amount = 45 min 2) Frequency = daily 3) Duration = 6 weeks e) Daily diary, weekly telephone follow-up, usual rehabilitation therapy by local stroke team.	Baseline, 6 weeks, 6 months Primary outcome: 1) ARAT Secondary outcome: 1) Canadian Occupational Performance Measure 2) SIS 3) mRS 4) EQ-5D 3L 5) MAL-14* * only measured at 6 months	a) No significant differences for all outcomes b) 12.1%*
Ali et al. (2024) India	Multi-centered, single-blinded, RCT	a) N = 120 IG = 64, CG = 56 b) IG: mean age = 54.4 ± 11.7 CG: mean age = 57.7 ± 10.9 c) IG: Female = 14 CG: Female = 15 d) IG: ischemic stroke = 53, hemorrhagic stroke = 11 CG: ischemic stroke = 47, hemorrhagic stroke = 9 e) Acute/subacute stroke survivors	Rehabilitation center / physical therapist	a) Rehabilitation centers b) Physical therapist c) Received a gamified upper limb program (functional games) using the ArmAble device + conventional therapy. d) Dose: 1) Amount = 45–60 min/day 2) Frequency = 6 days/week 3) Duration = 2 weeks e) Received conventional therapy. For post-intervention - Received a functional upper limb rehabilitation home-based program - 30 min/day for 6 days/week for 4 weeks - Logbook - Weekly telephone follow-up	a) Rehabilitation centers b) Physical therapist c) Received upper limb task-based training + conventional therapy d) Dose: Amount = 45–60 min/day Frequency = 6 days Duration = 2 weeks e) Conventional therapy consisted of facilitatory/inhibitory techniques, strength training, passive and active limb mobilization, balance, and ambulatory training - 45 to 60 min/day For post-intervention - Received a functional upper limb rehabilitation home-based program - 30 min/day for 6 days/week for 4 weeks - Logbook - Weekly telephone follow-up	Baseline, 2nd week (immediate post-intervention), 6th week (follow-up assessment) Primary outcomes: 1) Upper limb function and activity - FM-UE and ARAT Secondary outcomes: 1) upper limb strength - motricity index (MI)-arm 2) degree of disability - mRS 3) Impact of the consequences of stroke - SIS-V 3.0 * game user experience - game user experience satisfaction scale (GUESS) only for IG at 2nd week	a) At 2 weeks post-intervention, a statistically significant improvement was observed for IG in the FM-UE (*p* =.003). At 6 weeks, FM-UE and ARAT scores were significantly higher in the IG than in the CG (*p* = .003 and *p* = .046, respectively). No significant differences were found in other outcomes. Adherence to exercise training at home during the 4-week period was 89.1% in the IG and 85.8% in the CG. b) 18.3%
Allegue et al. (2022) Canada	2-arm feasibility clinical trial	a) N = 11 IG = 6, CG = 5 b) IG*: mean age = 57.8 ± 21.8 CG^ϯ^: mean age = 56.4 ± 17.3 c) IG: Female = 2 CG: Female: 3 d) IG: ischemic stroke = 2, hemorrhagic stroke = 1 (1 participant in the IG had an unspecified type of stroke) CG^ϯ^: ischemic stroke = 3, hemorrhagic stroke = 2 e) Chronic stroke survivors * data from 4 participants ^ϯ^ data from 5 participants	Home-based / remote control by clinician	a) Home-based b) Clinician c) VirTele program: Before intervention - 1-h training session to familiarize with the Jintronix exergames (5 games) A) Jintronix exergames - upper extremity rehabilitation d) Dose: 1) Amount = 30 min/session 2) Frequency = 5 days/week 3) Duration = 8 weeks e) Reacts app - videoconference sessions with clinicians synchronized with sessions when the participant was playing exergames, used by the clinician to provide motivational interviewing to the participant	a) Home-based b) Participants were offered one session with a clinician c) Usual care: Graded Repetitive Arm Supplementary Program (GRASP) - home rehabilitation training program that included exercises for the arm and hand and functional activities targeting the upper extremity. d) Dose: 1) Amount = 30 min/session 2) Frequency = 5 days/week 3) Duration 8 weeks	Baseline, 8 weeks (post-intervention), 12 weeks (1 month post-intervention), 16 weeks (2 months post-interventions) Primary outcome: 1) FMA-UE Secondary outcomes: 1) MAL-30 2) SIS-16 3) TSRQ-15	a) FMA-UE and Motor Activity Log-30 - IG and CG demonstrated an improvement in >50% of the participants, from 1 month post-intervention to 2 months post-intervention. SIS-16 scores - CG reported improvement in activities of daily life (3/5, 60%), hand function (5/5, 100%), and mobility (2/5, 40%), from baseline to 2 months post-intervention. TSRQ-15 - 75% of the participants in IG demonstrated an increase in the autonomous motivation score from baseline to immediate post-intervention. b) 18.2%
Amin et al. (2024) Pakistan	Single-centered, RCT	a) N = 52 IG = 26, CG = 26 b) IG: mean age = 51.8 ± 12.9 CG: mean age = 49.8 ± 9.9 c) IG: Female = 10 CG: Female = 8 d) Type of stroke not mentioned e) Subacute stroke survivors	Physiotherapy department / physical therapist	a) Physiotherapy department b) Physical therapist c) VR based game intervention (immersive VR settings with VR headset) plus conventional physical therapy. Games were developed with the Unity3D game engine and run by the Android Package Kit (APK file) on the Oculus Quest 2 Virtual Reality device. d) Dose: 1) Amount = 24 min of VR hand games and 24 min of therapy (first 2 weeks); 40 min of VR hand games and 40 min of conventional therapy (next 4 weeks) 2) Frequency = 4 days/week 3) Duration = 6 weeks	a) Physiotherapy department b) Physical therapist c) Conventional physical therapy: Range of Motion (ROM), stretching, resistance, and strengthening exercises. d) Dose: 1) Amount = 48 min (Week 1 and Week 2); 80 min (for the next 4 weeks) 2) Frequency = 4days/week 3) Duration = 6 weeks	Baseline, Week 4, Week 6, Week 9 Primary outcomes: 1) FMA-UE 2) ARAT 3) Hand dexterity - BBT Secondary outcomes: 1) Capacity to carry out instrumental and everyday activities - MBI* 2) Quality of life - SSQOL* *Only assessed at baseline and Week 9	a) IG demonstrated significantly greater scores in FMA-UE, ARAT, and BBT at week 4, 6, and 9 when compared with CG (*p* <.005). IG demonstrated significantly greater scores in MBI and SSQOL when compared with CG (*p* <.001) b) 0%
Cano-Mañas et al. (2020) Spain	Single-centered, RCT	a) N = 56 IG = 28, CG = 28 b) Mean age = 63.13 ± 10.38 IG: mean age = 60.35 ± 9.84 CG: 65.68 ± 10.39 c) IG: Female = 11 CG: Female = 14 d) IG: ischemic stroke = 17, hemorrhagic stroke = 6 CG: 14, ischemic stroke = 15, hemorrhagic stroke = 10 e) Subacute stroke survivors	Hospital / physical therapist	a) Hospital b) Physical therapists c) Video game-based therapy using Xbox 360° video games console and the Kinect (20 min) plus conventional rehabilitation (35 min of physical therapy + 35 min of occupational therapy) d) Dose (for intervention): 1) Amount = 20 min 2) Frequency = 3 times/week 3) Duration = 8 weeks e) Otherwise followed the conventional rehabilitation schedule	a) Hospital b) Physical therapists c) Conventional rehabilitation - task-oriented motor training d) Dose: 1) Amount = 45 min of physical therapy and 45 min of occupational therapy 2) Frequency = 5 times/week 3) Duration = 8 weeks	Baseline, 8 weeks Outcomes: 1) mRS 2) BI 3) Tinetti Scale for Balance and Gait 4) Functional Reach Test 5) Get UP and Go Test 6) Baropodometry 7) EuroQoL 5D (5Q-5D) 8) Self-developed scale on satisfaction, adherence, and motivation with the treatment of video-game based therapy.	a) IG reported statistically higher mRS (*p* <.001), BI (*p* =.005), Tinetti gait assessment (*p*=.002), Functional Reach Test (*p* <.001), Get Up and Go Test (*p* =.005), EQ-5D [pain/discomfort dimension (*p* <.001), anxiety/depression dimension (*p* <.001)], VAS (*p* <.001). b) 14.3%
Choi et al. (2014) South Korea	Single-blind RCT	a) N = 20 IG = 10, CG = 10 b) IG: mean age = 64.30 ± 10.3 CG: mean age = 64.70 ± 11.3 c) IG: Female = 5 CG: Female = 5 d) IG: ischemic stroke = 8, hemorrhagic stroke = 2 CG: ischemic stroke = 6, hemorrhagic stroke = 4 e) Acute/ subacute stroke survivors (had a stroke within the last 90 days)	Hospital/ Occupational therapist	a) Department of rehabilitation b) Occupational therapist c) Commercial gaming based VR movement therapy using the Nintendo that consisted games like swordplay, table tennis, and canoe games. d) Dose (for intervention): 1) Amount = 30 min 2) Frequency = 5 times/week 3) Duration = 4 weeks e) Participants also received conventional rehabilitation therapy	a) Department of rehabilitation b) Occupational therapist c) Conventional occupational therapy that included stretching and strengthening exercises (full range of motion of the upper extremity) and chose appropriate tasks for each participant and developed them in stages. d) Dose: 1) Amount = 30 min 2) Frequency = 5 times/week 3) Duration = 4 weeks e) Participants also received conventional rehabilitation therapy	Baseline, at the end of the 4-week intervention Primary outcome: FMA-UL Secondary outcomes: 1) MFT 2) BBT 3) Grip strength was evaluated using a dynamometer 4) K-MMSE 5) Visual and auditory continuous performance tests (CPTs) to assess attention 6) K-MBI	a) There were no statistically significant differences between groups in FMA-UL (*p* = .63), MFT (*p* = .48), BBT (*p* = .43), grip strength (*p* = .53), K-MMSE (*p* = .22), CPT (*p*-values ranged from 0.12–0.85), and K-MBI (*p* = .53). b) 0%
Dabrowska et al. (2025) Czech Republic Dabrowska et al. (2023) *Another report	RCT with a parallel group	a) N = 70 IG = 35, CG = 35 b) IG: mean age = 59.4 ± 8.9 CG: mean age = 63.0 ± 8.8 c) IG: Female = 12 CG: Female = 12 d) Type of stroke not mentioned e) Acute/subacute stroke survivors	Rehabilitation sanatorium/ Physiotherapist or occupational therapist	a) Rehabilitation center b) Physiotherapist or occupational therapist c) VR therapy using the Oculus Quest 2 and VITALIS Pro VR (3 times/week) plus conventional rehabilitation. A total of four programs: 1) free painting; 2) 2D tracing, 3) 3D painting, and 4) puzzle. Each task has three types of environments: 1) forest; 2) space; and 3) sea. d) Dose (for intervention): 1) Amount = 30 min/session 2) Frequency = 3 times/week 3) Duration = 4–5 weeks	a) Rehabilitation centre b) Conventional therapy – individual physical therapy, occupational therapy, and miscellaneous therapies (e.g., iodobromine bath and wrap, oxygen therapy, etc.) d) Dose: 1) Amount = 30 min of physical therapy, 30 min of occupational therapy 2) Frequency = 2 times/week 3) Duration = 4–5 weeks	Baseline, immediately post intervention, 4 weeks post intervention, and one year post intervention. Outcomes: 1) MMSE 2) BI 3) EBI 4) WHODAS 2.0	a) No significant differences were found between IG and CG in any of the outcomes. b) 28.6% (post intervention) and 74.3% (1 year post intervention)
da Silva Ribeiro et al. (2015) Brazil	Single-blind RCT	a) N = 30 IG = 15, CG = 15 b) IG: mean age = 52.8 ± 8.6 CG: mean age = 53.7 ± 6.1 c) IG: Female = 10 CG: Female = 9 d) Type of stroke not mentioned e) Chronic stroke	Not specified / Physical therapists	a) Not specified b) Physical therapist c) Virtual rehabilitation via Nintendo Wii: Received an intervention in a 20 m2 room equipped with the Nintendo Wii and a multimedia projector. Started with a 10-min stretching including upper and lower limbs, and trunk muscles. 50-min protocol of Nintendo Wii games with 1 min rest interval between each game. d) Dose: 1) Amount:1 h (50 mins Nintendo Wii games) 2) Frequency = Twice/week 3) Duration = 8 weeks	a) Not specified b) Physical therapist c) Conventional physical therapy: including activities such as stretching, active-resisted mobilization of the trunk, straightening, balancing, gripping activities, scapular mobilization, active/active-assisted diagonal movement of the upper limbs d) Dose: 1) Amount = 60 mins/session 2) Frequency = Twice/week 3) Duration = 8 weeks	Baseline, 8 weeks (post-intervention) Outcomes: 1) SF-36 2) Fugl–Meyer (FM)	a) IG reported to have significantly higher SF-36 scores in physical functioning post-intervention when compared with CG (*p* <.001). No other significant differences were observed. b) 0%
de Rooij et al. (2021) Netherlands	Double-blinded RCT with two parallel groups	a) N = 55 IG = 28, CG = 27 b) IG: mean age = 65 (57–70) CG: mean age = 61 (53–71) c) IG: Female = 10 CG: Female = 6 d) IG: ischemic stroke = 24, hemorrhagic stroke = 4 CG: ischemic stroke = 20, hemorrhagic stroke = 4 e) Subacute stroke survivors *SD for age not specified, only age range	Rehabilitation center/hospital Physical therapists	a) Rehabilitation center b) Physical therapists c) Received VR gait training (VRT) on the Gait Real-time Analysis Interactive Lab (GRAIL), which is a dual-belt treadmill combined with a motion-capture system and a screen (180° semi cylindrical). It consisted of different VR environments (to train reactive balance, maneuverability, or dual tasks with modifiable difficulty levels) with specific rehabilitation goals and real-time feedback. d) Dose: 1) Amount = 30 min/session 2) Frequency = twice/week 3) Duration = 6 weeks	a) Rehabilitation center b) Physical therapists c) Received conventional treadmill training (10–15 min) and functional gait exercises which included 6 directional exercises (15 min). The intervention was progressive. c) Dose: 1) Amount = 30 min 2) Frequency = twice/week 3) Duration = 6 weeks	Baseline, 6 weeks, 3 months post-intervention Outcomes: 1) USER-P 2) SIS-16 3) Fatigue Severity Scale 4) HADS 5) Falls Efficacy Scale International 6) SSQOL 7) Timed “Up & Go” Test 8) 6-min walking test 9) Mini Balance Evaluation Systems Test 10) Daily-life walking activity measured with a triaxial accelerometer	a) Both groups showed significant improvements in participation and dynamic balance over time as quantified by the USER-P restrictions subscale (monthly increase of 2.53 points; 95% CI = 1.52 to 3.54, *p* < .001), the USER-P frequency subscale (1.05, 95% CI = 0.50 to 1.61, *p* < .001), and the Mini-BESTest score (0.62, 95% CI = 0.33 to 0.91, *p* < .001) No other significant outcomes b) 9.1%
Faria et al. (2016) Portugal	Single-blind RCT	a) N = 18 IG = 9, CG = 9 b) IG: median (IQR) age = 58 (48–71) CG: median (IQR) = 53 (50.5–65.5) c) IG: Female = 5 CG: Female = 5 d) Type of stroke not specified e) The phase of stroke: median (IQR) of 7 (4–49 for IG and 4 (3–11.5) for CG.	Hospitals / Psychologist or occupational therapist	a) Hospital b) Psychologist c) In addition to conventional motor rehabilitation, participants received cognitive rehabilitation through simulation of ADLs with the VR based system Reh@City (a desktop computer, a 24″ LCD monitor, and an arcade type of joystick). It consisted of interactive cognitive training (3-dimensional environment) to accomplish common ADLs in a supermarket, a post office, a bank and a pharmacy. Participants were given goal instructions in a task. It also allowed increase/decrease of the visuo-spatial orientation. There were visual feedback elements (to provide feedback on the accomplishment) and attention training tasks. d) Dose: 1) Amount = 20 min/session 2) Frequency = 12 sessions over 4 – 6 weeks	a) Hospital b) Occupational therapist c) In addition to conventional motor rehabilitation, participants received time-matched cognitive rehabilitation through traditional methods (e.g., puzzles, calculus, problem resolution, and shape sorting) c) Dose: 1) Amount = 20 min/session 2) Frequency = 12 sessions over 4 – 6 weeks	Baseline, post-intervention Outcomes: 1) ACE 2) Trail Making Test A and B (TMT A and B) 3) WAIS III 4) SIS 3.0 5) SUS	a) *Global cognitive functioning* - IG improved significantly more than CG in general cognitive functioning (assessed by ACE) (U = 13.500, Z=−2.388, *p*=.014, r =.56) and MMSE (U = 18.000, Z=−1.996, *p* =.050, r =.47). - IG demonstrated significantly higher scores in the attention domain (U = 17.000, Z=−2.066, *p* =.040, r=.49). - There were significant differences between groups in the fluency task (U = 13.000, Z=−2.487, *p* =.014, r=.59). *Attention* - No significant differences between group for the number of errors in TMT A (U = 40.000, Z=.047, *p* = 1, r=.01) and TMT B (U = 35.500, Z=−.482, *p* =.666, r=.11). Executive functions - No significant difference between groups in Picture Arrangement test scores (WAIS III). *QoL* - No significant differences between groups under SIS. *Usability* - Good levels of usability and satisfaction (Mdn=80/100, IQR=75–87.5). b) 0%
Huber et al. (2025) Switzerland	Single-blind, parallel, RCT	a) N = 46 IG = 24, CG = 22 b) IG: mean age = 68.75 ± 8.51 CG: mean age = 63.18 ± 9.69 c) IG: Female = 5 CG: Female = 4 d) IG: ischemic stroke = 19, hemorrhagic stroke = 4 CG: ischemic stroke = 17, hemorrhagic stroke = 5 e) Chronic stroke survivors	Hospitals/ rehabilitation centers / Trained movement scientist/therapist	a) Hospitals, rehabilitation centers b) Trained movement scientist c) In addition to usual care, participants received concept-guided, personalized, motor-cognitive exergame (Dividat Senso which included pressure-sensitive plate, handrails in use, and a screen showing targets). Intervention provided personalized progression and variability in training considering principles for neuroplasticity, motor learning, and training. It also offered real-time feedback on the participant’s performance through visual, auditory, and tactile cues, which helped the participant engaged more effectively with the video games. d) Dose: 1) Amount = 30 – 40 min 2) Frequency = twice/week 3) Duration = 12 weeks e) Participants received one-to-one training sessions, supervised by trained movement scientists of the study team.	a) Hospitals, rehabilitation centers b) Not specified In addition to the usual care (physical and cognitive activities), participants also received a weekly phone call (5- to 10-min conversations) to balance contact to the study team. c) Dose: 1) Amount = 5–10 min 2) Frequency = weekly 3) Duration = 12 weeks	Baseline, 12 weeks, 24 weeks Outcomes: 1) MoCA 2) German SIS 3.0 3) Vienna Test System (VTS); SRT, TMT, NBT, MRT) 4) 10MWT 3) OWA	a) The SIS domain Mobility showed a significant interaction effect for IG (T2, ITT: *p* = .03, r = .24; PP: *p* = .06, r = .23). For cognitive assessment, a significant interaction effect in favor of the IG (intrinsic visual alertness) (T2, ITT: *p* = .02, r = .26; PP: *p* = .04, r = .25). Per protocol analyses showed significant interaction effects in favor of IG for mistakes in TMT-B (T1, *p* = .01; T2, *p* = .02), and NBT (T2, *p* = .02). No significant interaction effects for any parameter related to 10MWT. Outdoor gait speed (T1, ITT: *p* = .02, r = .25; PP: *p* = .11, r = 0.20) in favor of IG. Swing width unaffected measured outdoors (T1, ITT: *p* = .004, r = .31; PP: p = 0.02, r = 0.29 / T2, ITT: *p* = .003, r = .33; PP: *p* = .007, r = .33) in favor of IG. No other significant outcome findings. *Safety* - A participant with a pre-existing heart condition experienced an adverse event that was mild and potentially associated with the intervention in terms of timing, though not necessarily caused by it. b) 19.6%
Kilinc et al. (2023) Turkey	Assessor-blinded RCT	a) N = 30 IG = 15, CG = 15 b) IG: median age = 61 (46–65) CG: median age = 52.5 (42–65) c) IG: Female = 4 CG: Female = 7 d) IG: ischemic stroke = 11, hemorrhagic stroke = 4 CG: ischemic stroke = 12, hemorrhagic stroke = 2 e) Chronic stroke survivors	Hospital/ Physiotherapists	a) Department of Physical Medicine and Rehabilitation b) Physiotherapists c) A combination of virtual balance training (VBT) and conservative rehabilitation. VBT used Thera-Trainer Balo (TTB) device which comprises 2 cylinders connected to a base plate with knee support bars and pelvis support table. Sensors were used and an avatar was displayed on screen to determine the center of gravity. Activities included collecting objects arranged in a circle and dropping them into the storage unit of their center of gravity. Participants were trained at 50% range of motion and the difficulty level would be increased according to the precision and time pressure of the avatar when collecting objects. d) Dose: 1) Amount = 20 min/session (VBT) + 60 min conservative rehabilitation 2) Frequency = 4 times/week 3) Duration = 8 weeks	a) Department of Physical Medicine and Rehabilitation b) Physiotherapists c) Patient-specific conservative program which included exercises to 1) strengthen affected side, 2) increase range of motion, 3) strengthen muscles, 4) enhance balance and coordination. d) Dose: 1) Amount = 60 mins/session 2) Frequency = 4 times/week 3) Duration = 8 weeks	Baseline, post-intervention Primary outcomes: 1) BBS Secondary outcomes: 1) BI 2) NIHSS 3) BMR 4) FAS 5) SF-36	a) There were no significant differences observed between groups in BBS scores, BMR stages, FAS scores, SF-36 scale scores, and spasticity degrees at post-intervention. b) 3.3%
Kim (2018) South Korea	Single-blind RCT	a) N = 24 IG = 12, CG = 12 b) IG: mean age = 50.91 ± 9.57 CG: mean age = 57.23 ± 14.63 years c) IG and CG: Male = 15, Female = 9 d) No information e) Chronic stroke survivors	Not specified / occupational therapist	a) Not specified b) Occupational therapist c) Virtual reality exercise program using video games (Wii Sports and Wii Fit) + traditional rehabilitation d) Dose: 1) Amount = 40 min/session 2) Frequency = 3 times/week 3) Duration = 12 weeks	a) Not specified b) Occupational therapist c) Traditional rehabilitation (exercise program): d) Dose: 1) Amount = 30 min/session 2) Frequency = 5 times/week 3) Duration = 12 weeks	Baseline, 12 weeks (post-intervention) Outcomes: 1) Upper extremity function - FMA and MFT 2) SIS	a) A statistically significant difference in SIS scores was found between the two groups (*p* <.005). No statistically significant differences were found in FMA and MFT scores between groups. b) 0%
Kuo et al. (2023) Taiwan	Single-blind RCT	a) N = 37 IG = 19, CG = 18 b) IG: mean age = 57.47 ± 6.99 CG: mean age = 59.5 ± 10.65 c) IG: Female = 6 CG: Female = 3 d) IG: ischemic stroke = 10, hemorrhagic stroke = 9 CG: ischemic stroke = 4, hemorrhagic stroke = 14 e) Chronic stroke survivors	Not specified / occupational therapist	a) Not specified b) Occupational therapist c) VR game (a television, PABLO Tyromotion device, and immersive VR games) with two motion sensors and a grip sensor attached to the participant. No head-mounted device used. d) Dose: 1) Amount = 30 min/session (intervention) + 30 min of conventional occupational therapy 2) Frequency = 2 times/week 3) Duration = 9 weeks	a) Not specified b) Occupational therapist c) Standard rehabilitation -conventional occupational therapy program d) Dose: 1) Amount = 60 min/session 2) Frequency = 2 times/week 3) Duration = 9 weeks	Baseline, 9 weeks (immediate post-intervention) Primary outcome: 1) FMA-UE Secondary outcomes: 1) active ranges of motion (AROMs) of the shoulder and elbow 2) hand grip strength - Jamar dynamometer 3) unilateral gross manual dexterity - BBT 4) SIS (strength, hand function and ADL/IADL)	a) IG exhibited greater improvements in the hand dexterity (*p* = .05), shoulder flexion (*p* = .03) and elbow pronation between groups (*p* = .03). b) 8.1%
Laffont et al. (2020) France	Multicentric single-blind RCT	a) N = 51 IG = 25, CG = 26 b) IG: mean age = 60.8 ± 14.1 CG: mean age = 55.8 ± 14.0 c) IG: Female = 9 CG: F = 11 d) IG: ischemic stroke = 22, hemorrhagic stroke = 3 CG: ischemic stroke = 16, hemorrhagic stroke = 10 e) Subacute stroke survivors	Physical and rehabilitation medicine departments / occupational therapist	a) Hospital b) Occupational therapist c) VR + standard care [physiotherapy (30 min) and occupational therapy sessions (30–60 min) per day depending on patient's needs] d) Dose: 1) Amount = 15–45 min (virtual reality), 60–90 min (standard care) 2) Frequency = 5 times/week 3) Duration = 6 weeks	a) Hospital b) Occupational therapist c) Standard care [physiotherapy and occupational therapy sessions (a total of 60–90 min) per day depending on patient's needs] + additional sessions of occupational therapy d) Dose: 1) Amount = 15–45 min (additional occupational therapy), 60–90 min (standard care) 2) Frequency = 5 times/week 3) Duration = 6 weeks	Baseline, 6 weeks, 6 months Primary outcome: 1) UL-FMS Secondary outcomes: 1) BBT 2) Motor function of upper limbs - WMFT 3) MAL 4) SF-36 5) BI	a) At 6 weeks, gain in UL-FMS did not significantly differ between the groups (*p* = .10), but BBT was significantly improved the IG (*p* = .02). b) 9.8%
Lam et al. (2022) Hong Kong Lam et al. (2020) *Another report	Single-blind RCT	a) N = 93 IG = 47, CG = 46 b) IG: mean age = 65.1 ± 10.2 years CG: mean age = 66.0 ± 9.0 c) IG: Female = 20 CG: Female = 18 d) IG: ischemic stroke = 38, hemorrhagic stroke = 9 CG: ischemic stroke = 38, hemorrhagic stroke = 8 e) Subacute stroke survivors	Stroke rehabilitation in hospital / physiotherapists	a) Geriatric day hospital b) not specified for the intervention of computer games training c) Bilateral movement computer games training (3 different computer games) + conventional therapy (1.5 h of conventional physiotherapy and 1.5 h of multidisciplinary occupational therapy) d) Dose: 1) Amount = 30 min (computer games training) + 3 h conventional therapy 2) Frequency = 2 times/week 3) Duration = 8 weeks	a) Geriatric day hospital b) Monitored by a patient care assistant c) Video-directed conventional training (30 min) + conventional therapy (1.5 h of conventional physiotherapy and 1.5 h of multidisciplinary occupational therapy) d) Dose: 1) Amount = 30 min (video-directed conventional training) + 3 h conventional therapy 2) Frequency = 2 times/week 3) Duration = 8 weeks	Baseline, 4 weeks, 8 weeks (post-intervention), 12 weeks (4 weeks post-intervention) Primary outcome: 1) FMA-UE Secondary outcomes: 1) ARAT 2) Grip Strength - digital dynamometer 3) SF-36	a) Participants in IG demonstrated greater improvements in FMA-UE scores from mid-intervention to 1 month follow-up than CG. IG showed better improvements in ARAT scores than CG from post-intervention to 1 month follow. No significant differences were found in grip strength and SF-36 scores between groups. b) 10.8%
Latif et al. (2025) Indonesia	Single-centered, RCT	a) N = 60 IG = 30, CG = 30 b) IG: mean age = 58.43 ± 8.89 years CG: mean age = 53.97 ± 11.09 c) IG: Female = 9 CG: Female = 14 d) Type of stroke not mentioned e) A mixture of stroke survivors	Hospital outpatient care/therapists	a) Outpatient clinic b) Physiotherapists c) Received a combination of 1) VR therapy using MusicGlove Hand Therapy tools and 2) joint range of motion and fine motor exercises (conventional therapy) d) Dose (*for VR therapy and conventional therapy) 1) Amount = 1 h and 15 min/session 2) Frequency = not reported 3) Duration = 8 weeks	) Outpatient clinic b) Occupational therapists c) Received joint range of motion and fine motor exercises d) Dose: 1) Amount = 1 h/session 2) Frequency = not reported 3) Duration = 8 weeks	Baseline, 4 weeks, 8 weeks (post-intervention), 9 weeks (1 post-intervention) Outcomes: 1) Hand function - FMA-UE 2) Fine motor skills - 9-HPT	a) At week 4, both groups had no significant difference in hand functional status (*p* = .284). There were significant differences in hand functional status between groups at week 8 (*p* = .045) and 1 week after intervention (*p* = .037). There were significant changes in fine motor function between groups at all time points (*p* < .05), except between immediate post intervention and one week post intervention. b) 0%
Lee et al. (2016) South Korea	Single-blind RCT	a) N = 20 IG = 10, CG = 10 b) IG = 69.20±5.514 CG = 73.13±8.983 c) IG: Female = 5 CG: Female = 3 d) IG: ischemic stroke = 7, hemorrhagic stroke = 3. CG: ischemic stroke = 4, hemorrhagic stroke = 4. e) Chronic stroke survivors *These data do not include the two participants who dropped out	Hospital / Not specified	a) Hospital b) Not specified c) Participants received VR-based bilateral upper extremity training (VRBT) which consisted of visual feedback. The animation comprised: symmetric UE training (0° and 45°) and asymmetric UE training (0° and 45°). Equipment: a laptop, webcam, and a 23-inch monitor. d) Dose: 1) Amount = 30 min/session 2) Frequency = 3 days weekly 3) Duration = 6 weeks e) Also received conventional occupational therapy for 30 min/session, 5 days/week for 6 weeks	a) Hospital b) Not specified b) Bilateral upper extremity training (BT) – same UE training as VRBT group b) Dose: 1) Amount = 30 min/session 2) Frequency = 3 days weekly 3) Duration = 6 weeks e) BT group watched an irrelevant video. Also received conventional occupational therapy for 30 min/session, 5 days/week for 6 weeks	Baseline, post-intervention Outcomes: 1) JHFT 2) BBT 3) GPT 4) DMMT 5) Jamar Plus Hands on Evaluation Kit	a) IG demonstrated significant improvements in JHFT (five items, *p* <.05), BBT (*p* =.029), and GPT (*p* =.009) when compared with CG. Also, IG was statistically significantly improved in DMMT (elbow flexion/extension) and hand strength test (all *p*-values < .01) when compared to CG. b) 10%
Lee et al. (2017) Taiwan	Single-centered, RCT	a) N = 50 IG = 26, CG = 24 b) IG: mean age = 59.35 ± 8.95 CG: mean age = 55.76 ± 9.59 c) IG: Female = 10 CG: Female = 3 d) IG: ischemic stroke = 16, hemorrhagic stroke = 10 CG: ischemic stroke = 14, hemorrhagic stroke = 7 e) Chronic stroke survivors	Hospital / occupational therapist	a) Neurorehabilitation Unit b) Occupational therapist c) VR (comprised a television, Microsoft Kinect, and a commercial game) + standard treatment d) Dose: 1) Amount = 45 min interactive virtual reality balance-related games, 45 min standard treatment 2) Frequency = Twice/week 3) Duration = 6 weeks	a) Neurorehabilitation Unit b) Occupational therapist c) Standard treatment - strengthening, endurance training, ambulation, and ADL training d) Dose: 1) Amount = 90 min 2) Frequency = Twice/week 3) Duration = 6 weeks	Baseline, 6 weeks (post-intervention), 12 weeks (follow-up) Primary outcome: 1) BBS Secondary outcomes: 1) FRT16 2) TUG cognition test 3) MBI 4) ABC 5) SIS For both groups, modified Physical Activity Enjoyment Scale (M-PAES) and incidence for adverse events were recorded for each session.	a) Both groups demonstrated significant improvement over time in the BBS (*p* =.000) and TUG-cog test (*p* =.005). IG reported to have higher M-PAES scores compared to CG (*p* =.027). No other significant differences were found. b) 6%
Long et al. (2020) China	Assessor-blinded RCT	a) N = 60 IG = 30, CG = 30 b) IG = 53.28±15.30 CG = 54.11±14.81 c) IG: Female = 7 CG: Female = 11 d) IG: ischemic stroke = 21, hemorrhagic stroke = 4. CG: ischemic stroke = 19, hemorrhagic stroke = 8. e) Acute/ subacute /chronic stroke survivors (onset time ≤ 1 year)	Acute hospital / therapists	a) Acute hospital b) Therapist (not specified) c) Used VR-based game system (Doctor Kinetic) with a touch-controlled computer screen, infrared sensor smart recognition camera, and a human-shaped model on the computer screen. VR games consisted of five tasks including bilateral upper limb flexion; abduction activity; gold coins picking game, including shoulder circle; cross and mixed training for 3–5 min. Difficulty and intensity were adjusted according to the participant’s ability. d) Dose: 1) Amount = 45 min/session 2) Frequency = 5 times weekly 3) Duration = 3 weeks e) Received conventional training as well	a) Acute hospital b) Therapist (not specified) c) Received conventional training d) Dose: 1) Amount = 45 min/session 2) Frequency = 5 times weekly 3) Duration = 3 weeks	Baseline, post-intervention (after 3-week intervention) Outcomes: 1) COPM 2) SSEQ 3) FMA-UE 4) FTHUE 5) MBI	a) A significant difference between groups in SSEQ was observed after intervention (Median Difference = 8, *p* = .043, Z = − 2.027). And only daily activities domain in SSEQ demonstrated significance (Median Difference = 6, *p* = .017, Z = − 2.392). No significant differences in COPM were found between groups. A significant difference in MBI was found between groups (Median Difference = 10, *p* = .03, Z = − 2.171). No significant between-group difference in FMA-UE and FTHUE was noted, and both groups had improved upper limb function (*p* < .05). b) 13.3%
Marques-Sule et al. (2021) Spain	Single-blind RCT	a) N = 29 IG = 15, CG = 14 b) IG: mean age = 61.5 ± 8.4 CG: mean age = 58.2 ± 7.4, c) IG: Female = 6 CG: Female = 5 d) Type of stroke not specified e) Chronic stroke survivors	Lab of university rehabilitation clinic / Physical therapist	a) Lab of university rehabilitation clinic b) Physical therapist c) Virtual rehabilitation program using Nintendo Wii with the Wii Remote and Wii Balance Board in addition to conventional PT. d) Dose: 1) Amount = 30 min/session 2) Frequency = twice/week 3) Duration = 4 weeks e) Lower limb balance training (15 min) and upper limb training (15 min)	a) Lab of university rehabilitation clinic b) Physical therapist c) Received conventional PT c) Dose: 1) Amount = 30 min/session 2) Frequency = twice/week 3) Duration = 4 weeks	Pre-intervention, post-intervention Outcomes: 1) TUG 2) POMA 3) BBS 4) FMA-UL 5) BI 6) FAI	a) Significant differences in time for TUG (*p*< .001), POMA-total (*p* = .009), BBS (P = .003), FMA-UL (*p* = .0002), BI (*p* = .016), FAI (*p* < .001). Significant differences for group*time interaction for TUG (*p* = .018), POMA-total (*p* = .001), BBS (*p* = .002), BI (*p* = .016), FAI (*p* < .001) b) 0%
Mazher et al. (2025) Pakistan	Double blinded RCT	a) N = 32 IG = 16, CG = 16 b) IG: mean age = 52.94 ± 5.28 CG: mean age = 56.17 ± 6.44 c) IG: Female = 3 CG: Female = 6 d) IG: ischemic stroke = 11, hemorrhagic stroke = 7 CG: ischemic stroke = 14, hemorrhagic stroke = 2 e) Subacute stroke survivors	Hospital/ Not specified	a) Physical therapy department b) Not specified c) Xbox kinetic-based rehabilitation training for upper limbs (e.g., programs such as Boxing and Bowling in Kinect Sports Pack, Rally Ball, 20,000 leaks and Space Pop in the Kinect Adventure Pack) plus standardized physical therapy treatment d) Dose: 1) Amount = 30 min/session 2) Frequency = 5 times/week 3) Duration = 4 weeks	a) Physical therapy department b) Not specified c) Conventional exercise therapy in addition to training in daily tasks such as feeding, grooming, dressing, using the restroom, and transferring d) Dose: 1) Amount = 30 min/session 2) Frequency = 5 times/week 3) Duration = 4 weeks	Baseline, 4 weeks (post intervention), 8 weeks Outcomes: 1) FMA-UE 2) SIS Version 3.0	a) There were statistically significant differences between groups at post intervention and follow-up in QoL (*p* < .05). As for the upper extremity function, a statistically significant difference was observed between groups at 8 weeks. b) 0%
Oh et al. (2019) South Korea	Single-blinded RCT	a) N = 33 IG = 18, CG = 15 b) IG = 57.4 ± 12.2 CG = 52.6 ± 10.7 c) IG: Female = 5 CG: Female = 5 d) Type of stroke not specified e) Chronic stroke survivors	Not reported / Occupational therapist	a) Not specified b) Occupational therapist c) VR combined real instrument training. Joystim, a 3-dimensional manipulator consists of a monitor, conventional computer, and real instruments (e.g., thumb pinch, doorknob, button, air tube, gas valve, tool turn, steering wheel) with 3° of freedom. It consisted of nine modules, basic tools, games each, and two missions. The level of difficulty could be adjusted based on each participant’s performance during the training period. d) Dose: 1) Amount = 30 min/session 2) Frequency = 3 days weekly 3) Duration = 6 weeks	a) Not specified b) Occupational therapist c) Conventional occupational therapy which included UE training with task-related exercises and ADL board, hand fine motor training with pegboards, and perception and cognition training. The level of difficulty could be adjusted based on each participant’s performance during the training period. d) Dose: 1) Amount = 30 min/session 2) Frequency = 3 days weekly 3) Duration = 6 weeks	Pre-intervention. Post-intervention, 4 weeks post-intervention Outcomes: 1) FMA-UE 2) MMT 3) Hand grip test 4) BBT 5) 9-HPT 6) MAS 7) K-MMSE 8) K-MoCA	a) IG demonstrated significant improvements in the MMT (*p* =.039) and MAS wrist extension (*p* =.041), MAS for elbow flexion (*p* =.022), BBT (*p* =.002) in a time dependent manner. IG showed significant improvements in BBT (*p* =.010), 9-HPT (*p* =.025), lateral (*p* =.005), palmar (*p* =.012), and tip pinch (*p* =.006). Lateral pinch power was maintained at the follow-up (*p* =.002). Both groups significantly improved in MMT finger extension, FMA-UE, grip power, palmar pinch power, tip pinch power, 9-HPT, K-MMSE, and K-MoCA. Improvement rate in the tip pinch power (*p* =.036) scores and MAS for elbow flexion (*p* =.041) between the pretraining and post training periods was significantly higher in IG. b) 6.1%
Ozen et al. (2021) Turkey	Single-centered, RCT	a) N = 38 IG = 20, CG = 18 b) IG: mean age = 62.00 ± 13.12 CG: mean age = 69.8 ± 8.41 c) IG: Female = 5 GG: Female = 5 d) IG: ischemic stroke = 12, hemorrhagic stroke = 3 CG: ischemic stroke = 13, hemorrhagic stroke = 2 e) Subacute - chronic stroke patients (≥ three months since stroke)	University hospital / physiotherapist and occupational therapist	a) Physical Medicine and Rehabilitation ward b) Physiotherapist and occupational therapist c) Computer game assisted task specific exercises (CGATSE) + conventional therapy: 30 min CGATSE using the Rehabilitation Joystick for Computerized Exercise (Rejoyce) system d) Dose: 1) Amount = 30 min of CGATSE and 1 h physical therapy 2) Frequency = 5 times/week 3) Duration = 4 weeks	a) Physical Medicine and Rehabilitation ward b) Physiotherapist and occupational therapist c) Conventional neurorehabilitation physical therapy + occupational therapy: 1 h of proprioceptive neuromuscular and neurodevelopmental facilitation techniques, range of motion, strengthening exercises, balance-coordination, and ambulation training 30 min occupational therapy - task-based exercises d) Dose: 1) Amount = 30 min of occupational therapy, 1 h physical therapy 2) Frequency = 5 times/week 3) Duration = 4 weeks	Baseline, 4 weeks Primary outcomes: 1) FMA-UE 2) BSSR Secondary outcomes: 1) MoCA 2) SSQOL	a) While FMA-UE, BSSR arm, and SSQOL improved significantly in both groups (*p* < 0.05), no significant between-group differences were observed. b) 21.1%
Park and Ha (2023) South Korea	3-arm RCT (One of the control groups (CG1) is excluded in this review as it included computer-assisted cognitive rehabilitation with some game elements)	a) N = 41 IG = 21, CG2 = 20 b) IG: mean age = 62.5 ± 4.7 CG2: mean age = 62.5 ± 4.8 years c) IG: Female = 9 CG: Female = 8 d) IG: ischemic stroke = 11, hemorrhagic stroke = 9 CG: ischemic stroke = 12, hemorrhagic stroke = 8 e) Subacute - chronic stroke patients	Hospital/ Occupational therapist	a) Virtual reality room b) An occupational therapist c) One-on-one patient-specific individualized program with 30 min of conventional cognitive rehabilitation (morning) and 30 min of VR-based cognitive rehabilitation (afternoon). VR-based cognitive (grounded in self-efficacy theory) consisted of immersive programs (e.g., Finding same fishes’, ‘Save the planet’, ‘Throwing dar’, and ‘Other contents’) with goggles and controllers (15 min of VR and 15 of individual training using a stroke cognitive recovery workbook). Group discussions (4–5 participants in each group) were conducted every Friday. d) Dose: 1) Amount = 30 min (conventional) + 30 min (VR-based) 2) Frequency = 5 times/week 3) Duration = 8 weeks	a) Rehabilitation therapy room b) An occupational therapist c) Conventional cognitive rehabilitation (one-on-one) included paper-and-pencil tasks such as puzzles, calculation, picture matching, and others. d) Dose: 1) Amount = 30 min morning and afternoon 2) Frequency = 5 times/week 3) Duration = 8 weeks	Baseline, 4 weeks, 8 weeks Outcomes: 1) Stroke self-efficacy 2) Cognitive function – K-MMSE – 2:SV 3) Visual perception – MVPT-3 4) Activities of daily living – K-MBI 5) Health-related QoL – SF-12	a) There were statistically significant differences between groups and time points in stroke self-efficacy (F = 78.62, *p* < .001), cognitive function (F = 9.33, *p* < .001), ADLs (F = 14.15, *p* < .001), and HRQoL (F = 213.87, *p* < .001). b) 2.4%
Park et al. (2021) South Korea	Single-site RCT	a) N = 44 IG = 22, CG = 22 b) IG: mean age = 60.59 ± 18.12, CG: mean age = 62.29 ± 13.97 years c) IG: Female = 10 CG: Female = 10 d) Type of stroke not specified e) Acute stroke patients	Not reported/ Therapists	a) Not reported b) Therapists c) Game-based upper limb training with RAPAEL Smart GloveTM + conventional physical therapy. Functional training and ADLs such as catching butterflies and balls, squeezing an orange are adjusted based on the level of difficulty suitable for participants. d) Dose (intervention): 1) Amount = 30 min + 30 min (conventional physical therapy) 2) Frequency = 5 times/week 3) Duration = 4 weeks	a) Not reported b) Therapists c) Conventional physical therapy based on the exercise and tools to improve upper limb function (passive/active should joint and hand functions) and ADLs (basic, instrumental activities). d) Dose: 1) Amount = 30 min 2) Frequency = 5 times/week 3) Duration = 4 weeks	Baseline, post-intervention (end of 4-week) Outcomes: 1) Upper limb function - FMA-UE - Hand strength - JTHFT 2) ADLs - K-MBI	There were no significant interactions between time and group in FMA. There were significant differences in hand strength, JTHFT, and K-MBI between two groups over time. b) 0%
Rémy-Néris et al. (2021) France	Multi-centered, RCT	a) N = 215 IG = 107, CG = 108 b) IG: mean age = 58.08 ± 14.05 CG: mean age = 58.53 ± 13.27 c) IG: Female = 40 CG: Female = 35 d) IG: ischemic stroke = 77, hemorrhagic stroke = 30 CG: ischemic stroke = 75, hemorrhagic stroke = 33 e) Subacute stroke survivors	Hospital / physiotherapist and occupational therapist	a) Rehabilitation centers b) Physiotherapists and occupational therapists c) Gravity-supported, games-based training using an exoskeleton + usual rehabilitation: The ArmeoSpring exoskeleton device was used to train movements (shoulder and elbow), pronation and supination, and grip-release. d) Dose: 1) Amount = 30 min x 2 times game-based training, 1.5 h upper limb rehabilitation 2) Frequency = 5 days/week 3) Duration = 4 weeks	a) Rehabilitation centers b) Physiotherapists and occupational therapists c) Basic stretching and active exercises + usual rehabilitation d) Dose: 1) Amount = 30 min x 2 times basic stretching and active exercises, 1.5 h upper limb 2) Frequency = 5 days/week 3) Duration = 4 weeks	Baseline, 30 days, 3 months, 6 months, 12 months Primary outcome: 1) FMA-UE Secondary outcomes: 1) Change in sensorimotor impairment (FMA-UE) 2) Change in severity of shoulder pain - VAS 3) Change in spasticity - MAS 4) Function in self-care, continence, mobility, transfer, communication, and cognition - FIM 5) ARAT 6) EQ-5D 7) SIS 8) Cost utility 9) Participants' perception of exercise intervention	a) No significant between-group differences were observed in any outcome measures at any time point. b) 20%
Rodríguez-Hernández et al. (2021) Spain	2-arm, single-site RCT	a) N = 46 IG = 23, CG = 23 b) IG: mean age = 62.6 ± 13.5 CG: mean age = 63.6 ± 12.2 c) IG: Female = 5 CG: Female = 3 d) IG: ischemic stroke = 21, hemorrhagic stroke = 2 CG: ischemic stroke = 18, hemorrhagic stroke = 2 e) Subacute stroke survivors	General hospital/ Physiotherapist and occupational therapist	a) Hospital rehabilitation unit b) Physiotherapist and occupational therapist c) VR plus conventional therapy. Motor training with virtual reality devices: Hand Tutor© glove and 3DTutor© which were based on intensive and repetitive practice through movement and feedback instructions provided by the software with virtual environments and tasks that simulate movements that stroke survivors require for daily life. The Rehametrics© software [32] + Microsoft Kinect sensor was used for the recovery of the upper limb, trunk, and lower body. By monitoring and capturing real-time movement, the system allowed the therapist to adjust treatment parameters, including difficulty, duration, range of motion, and the number of distracting/visual aids. d) Dose: 1) Amount = VR (50 min) + conventional therapy (100 min) 2) Frequency = 5 consecutive days/week 3) Duration = 3 weeks	a) Hospital rehabilitation unit b) Physiotherapist and occupational therapist c) Conventional therapy consisted of manual therapy techniques (massage); passive and active assisted mobilization; walking in parallel; exercises with and without resistance; active-assisted mobility exercises (upper limb and fingers) in a sitting position; moving objects horizontally on a table; elevation and superposition of objects; biomechanical tasks. d) Dose: 1) Amount = 75 min of physiotherapy + 75 min of occupational therapy 2) Frequency = 5 consecutive days/week 3) Duration = 3 weeks	Baseline, 3 weeks after the start (post-intervention), and 3 months after its completion (follow-up) Outcomes: 1) EQ-5D-5L 2) EQ – VAS	a) IG had statistically significant higher EQ-VAS scores at post-intervention and follow-up when compared to CG. At post-intervention and follow-up, IG demonstrated significantly better HRQoL compared to CG across most EQ-5D-5 L dimensions (except for pain/ discomfort). b) 6.5%
Shin et al. (2015) South Korea	Single-blind, RCT	a) N = 35 IG = 18, CG = 17 b) IG: mean age = 53.3 ± 11.8 CG: mean age = 54.6 ± 13.4 c) IG: Female = 5 CG: Female = 3 d) No information e) Chronic stroke survivors	Not specified / occupational therapists	a) Not specified b) Occupational therapists c) VR + occupational therapy: Game-based VR rehabilitation with the RehabMaster™ system [compliant depth sensor, 3D awareness sensor, infrared projectors (60-inch monitor), and image sensors for VR]. Participants sat on a chair in front of the monitor and depth sensor and moved their upper limbs/trunk based on the training protocol. d) Dose: 1) Amount = 30 min virtual reality rehabilitation, 30 min conventional occupational therapy 2) Frequency = 5 days/week 3) Duration = 4 weeks	a) Not specified b) Occupational therapists c) Occupational therapy: Included range of motion and strengthening exercises, table-top activities, and activities of daily living training d) Dose: 1) Amount = 30 min conventional occupational therapy + 30 min additional occupational therapy 2) Frequency = 5 days/week 3) Duration = 4 weeks	Baseline, 4 weeks Outcomes: 1) SF-36 2) HAMD 3) FMA-UE	a) A significant difference between groups was observed for role limitation due to physical problems (*p* =.031). No other significant differences were found. b) 8.6%
Shin et al. (2016) South Korea	Single-blind, RCT	a) N = 46 IG = 24, CG = 22 b) IG: mean age = 57.2 ± 10.3 CG: mean age = 59.8 ± 13.0 c) IG: Female = 5 CG: Female = 5 d) IG: ischemic stroke = 15, hemorrhagic stroke = 9 CG: ischemic stroke = 14, hemorrhagic stroke = 8 e) There is no specified information found on the phase of stroke	National Rehabilitation Center / occupational therapist	a) Rehabilitation hospital b) Occupational therapists c) The RAPAEL Smart Glove: Glove-shaped sensor device and a software application (training games in the system with intended upper extremity movements). d) Dose: 1) Amount = 30 min Smart glove + 30 min standard occupational therapy 2) Frequency = 5 days/week 3) Duration = 4 weeks	a) Rehabilitation hospital b) Occupational therapists c) Standard occupational therapy d) Dose: 1) Amount = 1 h 2) Frequency = 5 days/week 3) Duration = 4 weeks	Baseline, middle of the intervention (after the 10th session), immediately after the intervention, and 1 month after the intervention. Primary outcome: 1) FMA-UE Secondary outcomes: 1) Hand function - JTT and PPT 2) SIS v3.0	a) The FM (FM total, *p* =.006; FM-prox, *p* =.007; FM-dist, *p* =.024, JTT (JTT total, p=.032; JTT-gross, *p* =.025), and SIS (composite, *p* =.021; overall score, *p* =.015) scores were significantly greater in IG than CG. No significant differences were found in PPT scores between two groups. b) 50%
Şimşek and Çekok (2016) Turkey	Single-centered, RCT	a) N = 44 IG = 22, CG = 22 b) IG: mean age = 54.15 ± 20.29 CG: mean age = 61.5 ± 11.63 c) IG: Female = 5 CG: Female = 8 d) IG: ischemic stroke = 11, hemorrhagic stroke = 9 CG: ischemic stroke = 9, hemorrhagic stroke = 13 e) Subacute stroke survivors	Medicalpark ˙Izmir Hospital (Physical Therapy and Rehabilitation) / physiotherapists	a) Department of Physical Therapy and Rehabilitation b) Physiotherapist c) Nintendo Wii video game (5 games) for upper limbs and balance training: 3 sets of game each, with 5 min interval d) Dose: 1) Amount = 45–60 min/session 2) Frequency = 3 times/week 3) Duration = 10 weeks	a) Department of Physical Therapy and Rehabilitation b) Physiotherapist c) Bobath neurodevelopmental treatment (conventional therapy): d) Dose: 1) Amount = 45–60 min/session 2) Frequency = 3 times/week 3) Duration = 10 weeks	Baseline, post-treatment (after 10 weeks) Outcomes: 1) FIM 2) NHP 3) VAS	a) No significant differences were found between groups with regard to FIM and NHP. IG reported higher satisfaction of treatment when compared with CG (*p* <.001). b) 4.5%
Song and Lee (2021) South Korea	Multi-centered RCT	a) N = 10 IG = 5, CG = 5 b) IG: mean age = 64.20 ± 7.08 CG: mean age = 60.00 ± 10.88 c) IG: Female = 2 CG: Female = 2 d) IG: ischemic stroke = 3, hemorrhagic stroke = 2 CG: ischemic stroke = 4, hemorrhagic stroke = 1 e) Subacute/ chronic stroke survivors (at least 6 months)	Hospital/ Not reported	a) Hospital (not specified) b) Multidisciplinary rehabilitation teams c) In addition to 60 min of conventional rehabilitation, participants also had a VR-based bilateral arm training intervention which consisted of 1) daily life training, 2) visual perception and cognitive component, 3) exercise evaluation component, and 4) 14 visual perception tasks. DK2 Oculus Rift and Oculus Rift controller were used (immersive VR experience), participants were sitting while performing the training. d) Dose (intervention): 1) Amount = 30 min 2) Frequency = 5 times/week 3) Duration = 4 weeks	a) Hospital (not specified) b) Multidisciplinary rehabilitation teams c) Normal bilateral arm training group (e.g., turning on lights, arranging a chest of drawers) in a real environment + 60 min conventional rehabilitation d) Dose: 1) Amount = 30 min 2) Frequency = 5 times/week 3) Duration = 4 weeks	Baseline, post-intervention (after 4 weeks) Outcomes: Upper extremity function: EMG - MFT - Sensory function test (two-point discrimination, proprioception, and stereognosis).	There was no statistically significant difference between groups in MFT (*p* = .07), EMG analysis and sensory function tests except for proprioceptive test that showed a significant difference between groups (*p* = .04) b) 0%
Térémetz et al. (2022) France	Single-centered, RCT	a) N = 43 IG = 21, CG = 22 b) IG: mean age = 55.8 (95% CI: 49.7 - 61.9) CG: mean age = 56.2 (95% CI: 50.5 - 61.9) c) IG: Female = 6 CG: Female = 10 d) No information e) Chronic stroke survivors	Hospital / physiotherapist	a) University hospital b) Physiotherapist c) Wii therapy (3 games): Tennis, gold, and boxing 15 min of each sitting on a stool d) Dose: 1) Amount = 1 h 2) Frequency = 3 times/week 3) Duration = 4 weeks	a) University hospital b) Physiotherapist c) Conventional therapy: Focused on functional exercise - passive and active movements of impaired joints and functional, task-orientated reaching and grasping exercises d) Dose: 1) Amount = 1 h 2) Frequency = 3 times/week 3) Duration = 4 weeks	Baseline, the week after intervention Primary outcome: 1) change in elbow extension and forward trunk motion Secondary outcomes: 1) Pain - VAS 2) Perceived effort - 10-point Borg scale 3) FMA-UE 4) BBT 5) ARAT 6) MAL 7) SIS	a) No significant differences in mean change in elbow extension angle (*p* =.61) and forward trunk position (*p* =.65). No significant between-group differences in change in FMA-UE, ARAT, BBT, MAL scores, and SIS. b) 7%
Velmurugan et al. (2023) India	Parallel group design, single-blind RCT	a) N = 40 IG = 20, CG = 20 b) IG: mean age = 52.45±6.24 CG: mean age = 53.65±5.87 c) IG: Female = 7 CG: Female = 6 d) IG: ischemic stroke = 16, hemorrhagic stroke = 4 CG: ischemic stroke = 14, hemorrhagic stroke = 6 e) Subacute and chronic stroke survivors	Department of physiotherapy in a college/ physiotherapists	a) Physiotherapy department b) Physiotherapist c) Five games from Nintendo Wii (N-Wii) software video games including Wii sports and Wii flit for upper limbs (e.g., tennis, punches out, light-rope tension, tilt table, and heading) d) Dose: 1) Amount = each games x 3 sets with 5 min intervals between sets (a total of 45 min) 2) Frequency = 5 sessions/week 3) Duration = 6 weeks	a) Physiotherapy department b) Physiotherapist c) Active control: Motor relearning program (e.g., activities such as opening/closing bottle lids and drinking water in a glass). d) Dose: 1) Amount = each task was repeated 10–15 min on affected side (a total of 45 min) 2) Frequency = 5 sessions/week 3) Duration = 6 weeks	Baseline, after 6 weeks of intervention Outcome: Upper limb motor function – FMA	a) The intervention group demonstrated significantly higher improvement in FMA- upper limb motor recovery when compared to the control group. b) 0%

Note: 9-HPT = 9-Hole Peg Test; 10MWT = 10-Meter Walk Test; ABC = Activities-specific Balance Confidence; ACE = Addenbrooke Cognitive Examination; ARAT = Action Research Arm Test; BBS = Berg balance scale; BBT = Box and Block Test = BBT; BI = Barthel Index; BMR = Brunnstrom motor recovery; CG = control group; COPM = Canadian Occupational Performance Measure; EBI = Extended Barthel Index; EMG = Electromyography; FAI = Frenchay Activity Index; FAS = Functional Ambulatory Scale; FIM = Functional Independence Measure; FM = Fugl-Meyer; FMA-UL = Fugl-Meyer Assessment Upper Limb; FRT 16 = Functional Reach Test 16; FTHUE = Functional Test for the Hemiplegic Upper Extremity; HADS = Hospital Anxiety and Depression Scale; HAMD = Hamilton Depression Rating Scale; IG = intervention group; JTT = Jebsen–Taylor hand function test; K-MBI = Korean version of the Modified Barthel Index; K-MMSE = Korean version of the Mini-Mental State Examination; K-MoCA = Korean-Montreal Cognitive Assessment; MAL = Motor Activity Log; MAS = Modified Ashworth Scale; MBI = Modified Barthel Index; MFT = manual function test; MMDT = Minnesota Manual Dexterity Test; MMSE = Mini Mental State Examination; MoCA = Montreal Cognitive Assessment; mRS = Modified Rankin Scale; MVPT-3 = Motor-Free Visual Perception Test, 3rd edition; NHP = Nottingham Health Profile; NIHSS = National Institute of Health Severity Scale; OWA = Outdoor Walking Assessment; POMA = Performance-oriented mobility assessment; PPT = Purdue pegboard test; SIS = Stroke Impact Scale; SSEQ = Stroke Self-Efficacy Questionnaire; SSQOL = Stroke-Specific Quality of Life; SUS = System Usability Scale; TSRQ-15 = Treatment Self-Regulation Questionnaire-15; TUG = Timed up and go; USER-P = Utrecht Scale for Evaluation of Rehabilitation-Participation; VAS = visual analogue scale; WAIS III = Wechsler Adult Intelligence Scale III; WMFT = Wolf Motor Function Test.

### 3.3 Characteristics of participants

Among the 34 included studies, the highest average age reported for participants in the intervention and control groups was 69.2 years (Lee et al., 2016) and 69.8 years (Ozen et al., 2021), respectively. Twenty-three studies specifically mentioned the inclusion of participants with both ischemic and hemorrhagic stroke. Conversely, 11 studies did not specify the type of stroke experienced by the participants (Amin et al., 2024; Dabrowska et al., 2025; da Silva Ribeiro et al., 2015; Kim, 2018; Latif et al., 2025; Marques-Sule et al., 2021; Oh et al., 2019; Park et al., 2021; Shin et al., 2015; Song and Lee, 2021; Térémetz et al., 2022). The phases of stroke in the included studies were categorized into acute, subacute, and chronic. The subacute phase is identified as occurring 15 to 180 days following the initial stroke, while the chronic phase begins at 180 days post-stroke, marked by typically slow or no clinical progress (Ammann et al., 2014). One study focused on acute stroke survivors (Park et al., 2021). Nine studies focused on subacute stroke survivors (Amin et al., 2024; Cano-Mañas et al., 2020; de Rooij et al., 2021; Laffont et al., 2020; Lam et al., 2022; Mazher et al., 2025; Rémy-Néris et al., 2021; Rodríguez-Hernández et al., 2021; Şimşek and Çekok, 2016). Thirteen studies examined chronic stroke survivors (Allegue et al., 2022; da Silva Ribeiro et al., 2015; Huber et al., 2025; Kilinc et al., 2023; Kim, 2018; Kuo et al., 2023; Lee et al., 2017, 2016; Marques-Sule et al., 2021; Oh et al., 2019; Shin et al., 2015; Song and Lee, 2021; Térémetz et al., 2022). Four studies included participants across both acute and subacute phases (Adie et al., 2017; Ali et al., 2024; Choi et al., 2014; Dabrowska et al., 2025), and three studies included participants across both subacute and chronic phases (Ozen et al., 2021; Park and Ha, 2023; Velmurugan et al., 2023). Three studies did not specify the phase of stroke (Faria et al., 2016; Latif et al., 2025; Shin et al., 2016). Long et al. (2020) included stroke survivors with onset time ≤ 1 year.

### 3.4 Characteristics of interventions

#### 3.4.1 Setting and providers

Game-based interventions were predominantly conducted at rehabilitation centers or hospitals. Two studies reported that the interventions were conducted at participants’ homes (Adie et al., 2017; Allegue et al., 2022). Velmurugan et al. (2023) conducted the intervention in a college. Seven studies did not specify the intervention settings (da Silva Ribeiro et al., 2015; Kim, 2018; Kuo et al., 2023; Lee et al., 2016; Oh et al., 2019; Park et al., 2021; Shin et al., 2015).

All interventions were provided by either physical therapists, physiotherapists, or occupational therapists, with the following exceptions: one study was remotely monitored by a clinician (Allegue et al., 2022); one study used a psychologist to provide cognitive rehabilitation (Faria et al., 2016); and one used a trained movement scientist (Huber et al., 2025). No information was provided on the interventionist for two studies (Lee et al., 2016; Mazher et al., 2025), and another two studies only mentioned ‘therapist’ as the interventionist (Long et al., 2020; Park et al., 2021).

#### 3.4.2 Types of VR-based rehabilitation

Twenty-six studies utilized non-immersive VR without head-mounted devices, two studies used semi-immersive VR (de Rooij et al., 2021; Shin et al., 2015), and four studies employed immersive VR (Amin et al., 2024; Dabrowska et al., 2025; Park and Ha, 2023; Song and Lee, 2021). The level of immersion was unable to be determined in two studies due to insufficient information (Latif et al., 2025; Velmurugan et al., 2023).

Of the included studies, 13 used mainstream entertainment or consumer gaming systems, such as the Microsoft Kinect (Lee et al., 2017), Nintendo Wii (Adie et al., 2017; Choi et al., 2014; da Silva Ribeiro et al., 2015; Kim, 2018; Marques-Sule et al., 2021; Şimşek and Çekok, 2016; Térémetz et al., 2022; Velmurugan et al., 2023), Oculus Quest 2 (Amin et al., 2024; Dabrowska et al., 2025), and Xbox 360° (Cano-Mañas et al., 2020; Mazher et al., 2025). Another 15 studies used non-commercial or dedicated rehabilitation gaming platforms and devices, including the AbleX (Lam et al., 2022), ArmAble (Ali et al., 2024), Armeo Spring (Rémy-Néris et al., 2021), Dividat Senso (Huber et al., 2025), Doctor Kinetic (Long et al., 2020), Hand Tutor glove and 3Dtutor (Rodríguez-Hernández et al., 2021), Jintronix (Allegue et al., 2022), JoyStim (Oh et al., 2019), and PABLO (Kuo et al., 2023), RAPAEL Smart Glove (Park et al., 2021; Shin et al., 2016), Rehabilitation Joystick for Computerized Exercise (Rejoyce) (Ozen et al., 2021), Reh@City (Faria et al., 2016), Thera-Trainer Balo (TTB) (Kilinc et al., 2023), RehabMaster (Shin et al., 2015).

Four studies did not specify whether the gaming platform was commercial or non-commercial (Laffont et al., 2020; Latif et al., 2025; Lee et al., 2016; Park and Ha, 2023). Song and Lee (2021) used custom VR content that was developed by a professor, delivered through the DK2 Oculus Rift and Oculus Rift controller to provide immersive experience. Finally, one study delivered the VR-based intervention through the Gait Real-time Analysis Interactive Lab (GRAIL) (de Rooij et al., 2021).

#### 3.4.3 Delivery dose

Among the studies reviewed, individual session lengths ranged from a minimum of 15 min (Laffont et al., 2020) and to a maximum of 60 min (Ali et al., 2024; Rémy-Néris et al., 2021; Şimşek and Çekok, 2016; Térémetz et al., 2022). The frequency of interventions varied from daily (Adie et al., 2017) to only twice a week (da Silva Ribeiro et al., 2015; de Rooij et al., 2021; Huber et al., 2025; Kuo et al., 2023; Lam et al., 2022; Lee et al., 2017; Marques-Sule et al., 2021), while the overall duration of interventions ranged from two weeks (Ali et al., 2024) to 12 weeks (Huber et al., 2025; Kim, 2018). Notably, the most common intervention duration was four weeks (n = 10) among the included studies.

#### 3.4.4 Application of neurorehabilitation principles or theoretical frameworks

There are 15 principles of neurorehabilitation post-stroke, guiding the design and implementation of rehabilitation interventions that enhance optimal brain recovery and functional improvement. According to Maier et al. (2019), these include: 1) repetitive practice, 2) spaced practice, 3) dosage, 4) task-specific practice, 5) goal-oriented practice, 6) variable practice, 7) increasing difficulty, 8) multisensory stimulation, 9) rhythmic cueing, 10) explicit feedback/knowledge of results, 11) implicit feedback/knowledge of performance, 12) modulate effector selection, 13) action observation/embodied practice, 14) motor imagery, and 15) social interaction.

All studies included in this review explicitly reported the dosage of the game-based intervention. Almost all studies implemented game-based interventions with progressively increasing difficulty, with the exception of eight studies (Adie et al., 2017; Choi et al., 2014; Dabrowska et al., 2025; Latif et al., 2025; Lee et al., 2016; Mazher et al., 2025; Song and Lee, 2021; Térémetz et al., 2022). Twelve studies provided explicit or implicit feedback to participants (e.g., visual feedback, verbal feedback) (Allegue et al., 2022; Amin et al., 2024; de Rooij et al., 2021; Faria et al., 2016; Kuo et al., 2023; Lee et al., 2016, 2017; Rodríguez-Hernández et al., 2021; Shin et al., 2015, 2016; Şimşek and Çekok, 2016; Térémetz et al., 2022). Eight studies specifically highlighted the use of repetitive practice within their game-based interventions (Ali et al., 2024; Cano-Mañas et al., 2020; Ozen et al., 2021; Park et al., 2021; Rémy-Néris et al., 2021; Rodríguez-Hernández et al., 2021; Shin et al., 2016; Velmurugan et al., 2023). Three studies reported goal-oriented practice (da Silva Ribeiro et al., 2015; de Rooij et al., 2021; Faria et al., 2016). Notably, da Silva Ribeiro et al. (2015) selected Nintendo Wii games to achieve goals similar to those targeted in conventional rehabilitation.

The majority of studies did not incorporate a theoretical framework in the design or implementation of their game-based interventions. Notably, Allegue et al. (2022) and Park and Ha (2023) underpinned their VR-based interventions with self-determination theory (SDT) and self-efficacy theory, respectively. The former integrated SDT principles into motivational interviewing. Similarly, Kuo et al. (2023) and Lee et al. (2017) designed their VR game training protocols based on motor learning theory. In contrast, the remaining studies reviewed did not specify the use of any theoretical framework in the development of their interventions.

### 3.5 Feasibility and safety outcomes

Across 29 of the 34 included RCTs that reported screening figures, the participation rate ranged from 25.4% (Térémetz et al., 2022) to 100% (Allegue et al., 2022; Amin et al., 2024; de Rooij et al., 2021; Faria et al., 2016; Laffont et al., 2020; Oh et al., 2019, Ozen et al., 2021; Park and Ha, 2023; Park et al., 2021; Rodríguez-Hernández et al., 2021; Song and Lee, 2021) with a mean of approximately 84% and a median close to 95%. Most trials exceeded 80% participation among eligible patients, suggesting that, when offered, game‑based interventions were acceptable to the majority of stroke survivors. Five studies were clear outliers: Adie et al. (2017), Huber et al. (2025), Lee et al. (2016), Lee et al. (2017), and Shin et al. (2016) reported participation rate of 71%, 28.8%, 66.7%, 71.4%, and 64.8%, respectively. Térémetz et al. (2022) only had 25%, primarily due to a high refusal rate among otherwise eligible patients.

Retention after enrolment was generally good. Overall attrition ranged from 0% to 50%, with a mean of about 9% and a median of around 7%. All but one trial (Shin et al., 2016) reported withdrawal rates below 30%, a threshold commonly used to indicate feasible retention, and several trials (Amin et al., 2024; Choi et al., 2014; da Silva Ribeiro et al., 2015; Faria et al., 2016; Kim, 2018; Latif et al., 2025; Marques-Sule et al., 2021; Mazher et al., 2025; Park et al., 2021; Song and Lee, 2021; Velmurugan et al., 2023) reported no dropout. Attrition rates were broadly similar between game‑based and control arms, indicating that VR‑based programs were at least as acceptable as conventional therapy once participants had agreed to take part.

Adherence or engagement with the additional game‑based components was explicitly quantified in four trials (Ali et al., 2024; Allegue et al., 2022; Cano-Mañas et al., 2020; Laffont et al., 2020). In these studies, adherence ranged from 83% to 95% of the prescribed dose, with three of the four reporting values above 90%. Although adherence was under‑reported overall, the available data suggest that participants who initiated game‑based or home‑based training were generally able to complete most of the planned sessions or practice time, surpassing the ≥75% engagement threshold often used to indicate acceptable usability.

Adverse events were described in varying detail. Adie et al. (2017) and Rémy-Néris et al. (2021) reported a substantial number of serious adverse events (e.g. recurrent stroke, hospital readmission), but none were judged to be related to the trial interventions. Huber et al. (2025) reported that two participants withdrew during the intervention period due: one due to a non-casually related adverse event, and one due to an unrelated serious adverse event. When adverse events were broken down by group, their frequency and profile were similar in the intervention and control arms. Laffont et al. (2020) documented eight events possibly related to therapy (shoulder pain, complex regional pain syndrome, worsening spasticity), again distributed across both groups. Lee et al. (2017) reported 19 adverse events in the VR group versus 30 in the standard treatment group, consisting mainly of transient soreness, increased muscle tone, dizziness and pain that resolved with rest. Similarly, de Rooij. (2021) reported that 11 participants in the VR group and 15 participants in the conventional therapy group experienced events such as near-falls, dizziness, pain, and fatigue. Kuo et al. (2023) also noted shoulder soreness and transient dizziness in both groups.

In the remaining trials, either no adverse events were observed, or they were described as unrelated medical events or as minor symptoms leading to discontinuation in isolated cases. Importantly, no study reported a technology‑related serious adverse event, and VR‑based interventions were generally considered safe when delivered within structured rehabilitation programs.

### 3.6 Quality appraisal

**Fig. 2:**
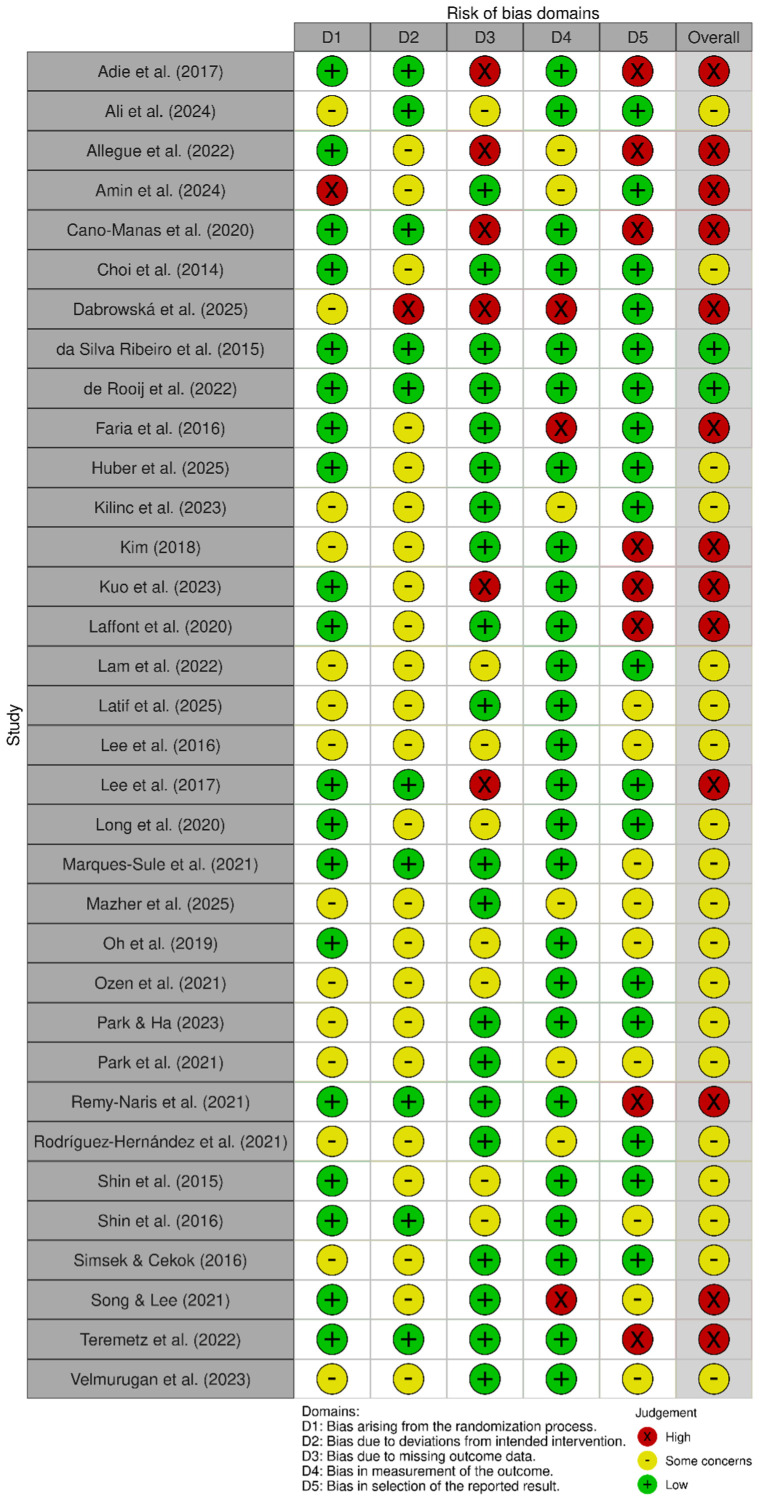
Risk of bias assessment of included studies using the Revised Cochrane Risk of Bias Assessment Tool for Randomized Trials (RoB2).

Overall, the Cochrane ROB 2.0 rated two studies with an overall “low” risk of bias, 19 studies with “some concerns” and the remaining 13 studies as a “high” overall bias (Fig. 2). Studies rated as “high” were mostly due to data presented, such as domain results reported instead of the total score, differences from baseline between groups and no data table provided (Kim, 2018). Adie et al. (2017) and Lee et al. (2017) analyzed results based on the initial allocated number of participants when the availability of data was <95%, influencing their true value. Adie et al. (2017) and Kim (2018) had multiple eligible outcome measurements with the use of two different scales for the same outcome. Some studies were unable to be blinded since knowledge of interventions was required. The GRADEpro software rated the QoL outcome of low quality, the UE functions as low to very low quality, and explanations are provided in Supplementary Material: Fig. S4.

### 3.7 Effectiveness of game-based interventions

#### 3.7.1 Quality of life

**Fig. 3:**
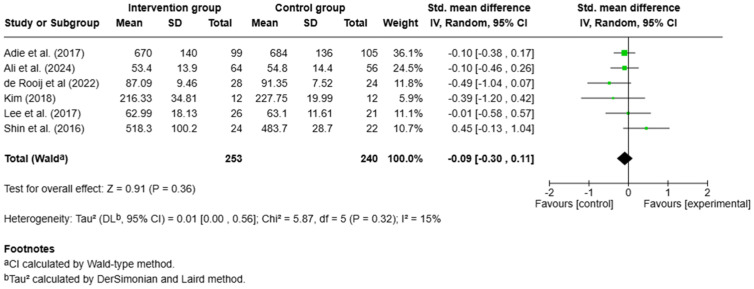
Forest plots: quality of life.

The QoL was assessed using the Stroke Impact Scale (SIS) (n = 13) (Adie et al., 2017; Ali et al., 2024; Allegue et al., 2022; de Rooij et al., 2021; Faria et al., 2016; Huber et al., 2025; Kim, 2018; Kuo et al., 2023; Lee et al., 2017; Mazher et al., 2025; Rémy-Néris et al., 2021; Shin et al., 2016; Térémetz et al., 2022), Short Form 36 (SF-36) (n = 5) (da Silva Ribeiro et al., 2015; Kilinc et al., 2023; Laffont et al., 2020; Lam et al., 2022; Shin et al., 2015), SF-12 (n = 1) (Park and Ha, 2023), EuroQoL (EQ-5D) (n = 2) (Cano-Mañas et al., 2020; Rémy-Néris et al., 2021), EQ-5D-5 L (n = 1) (Rodríguez-Hernández et al., 2021), Stroke-specific QoL (SS-QoL) (n = 3) (Amin et al., 2024; de Rooij et al., 2021; Ozen et al., 2021), WHODAS 2.0 (Dabrowska et al., 2025), and Nottingham Health Profile (NHP) (n = 1) (Şimşek and Çekok, 2016). Meta-analysis with random effects model for QoL was performed for the six studies involving 493 participants using SIS (Fig. 3). The pooled results showed a non-statistically significant small effect favoring conventional therapy (SMD = -0.09, 95% CI:0.30–0.11, *p* = .36) with non-statistically significant heterogeneity ($I^{2}$ = 15%, *p* = .32).

To explore potential sources of heterogeneity across studies, subgroup analyses were conducted based on: 1) neurorehabilitation principle (feedback versus no feedback), 2) intervention components (VR-based versus a combination of VR and conventional therapy/usual care), and 3) type of VR system/device (mainstream entertainment/consumer gaming system versus non-commercial/dedicated rehabilitation gaming platform/device). The pool effect estimates for the intervention group compared to control remained non-statistically significant across all subgroups (*p* > .05) (Supplementary Material, Fig. S1). However, moderate to substantial heterogeneity was observed in some subgroups (*I*^2^ = 33%, 62%).

Sensitivity analyses were performed to assess the robustness of the primary findings. First, a sensitivity analysis excluding all three studies rated as having high risk of bias (Adie et al., 2017; Kim, 2018; Lee et al., 2017) yielded results consistent with the primary analysis (primary: SMD = -0.09, 95% CI:0.30–0.11, *p* = .36 versus sensitivity: SMD = -0.06, 95% CI:0.52–0.40, *p* = .81) [Supplementary Material, Fig. S1 (d)]. Heterogeneity increased from *I^2^* = 15% (*p* = .32) to *I^2^* = 62 (*p* = .07). Second, a sensitivity analysis excluding only Shin et al. (2016), the study with the highest attrition rate from the primary analysis also yielded consistent results, with heterogeneity reduced from *I^2^* = 15% (*p* = .32) to *I^2^* = 0% (*p* = .7) [Supplementary Material, Fig. S1 (e)]. When Shin et al. (2016) was removed (highest attrition rate) from the subgroup analysis comparing ‘mainstream entertainment/consumer gaming system’ versus ‘non-commercial/dedicated rehabilitation gaming platform/device’, there remained no statistically significant differences between both subgroups, with heterogeneity of *I^2^* = 0% and *I^2^* = 25% [Supplementary Material, Fig. S1 (f)].

Narratively summarizing, the findings on QoL across included studies are mixed. Amin et al. (2024) found that the immersive VR game intervention led to significant improvement in QoL compared to the control group at the 9th week follow-up, following a six-week intervention (*p*<.001). Similarly, Kim (2018), Mazher et al. (2025), Shin et al. (2016), and Şimşek and Çekok (2016) reported significant improvements in QoL at post-intervention with the use of game-based interventions (all *p*-values of <.05). Huber et al. (2025) found significant interaction effects favoring the exergame group, as measured by SIS 3.0 scores. Rodríguez-Hernández et al. (2021) reported that, at post-intervention, the intervention group demonstrated significantly better EQ-5D-5 L scores across all dimensions (except pain/discomfort) compared to control group.

Two studies reported significant effects in specific QoL domains favoring the game-based intervention. Cano-Mañas et al. (2020) found significant improvements in the domains of anxiety or depression (*p* <.01), pain or discomfort (*p* <.01), and the visual analogue scale (*p* <.01). Shin et al. (2015) reported significant improvement s in the domain of role limitation due to physical problems (*p* =.031). However, the latter finding contrasts with da Silva Ribeiro et al. (2015), where physical functioning outcomes favored conventional therapy over the game-based intervention.

Eight studies reported no significant difference between groups at post-intervention (all *p* values > .05) (Faria et al., 2016; Kilinc et al., 2023; Kuo et al., 2023; Laffont et al., 2020; Lam et al., 2022; Ozen et al. (2021); Rémy-Néris et al. (2021) and Térémetz et al., 2022). Inconclusive findings were also reported. Allegue et al. (2022) found inconsistent results for the intervention group across four time points using the SIS-16 scores, whereas the control group showed improvements in activities of daily life, hand function, and mobility. Park and Ha (2023), using the SF-12, reported significant group-by-time interactions in health related QoL favoring the intervention group.

#### 3.7.2 Upper extremity functions

**Fig. 4:**
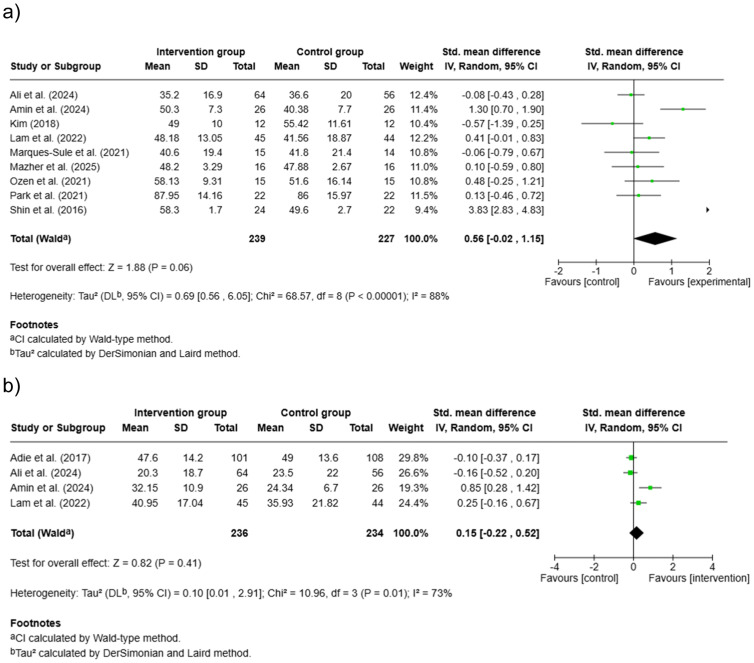
Forest plots: (a) upper motor functions measured by Fugl Meyer, (b) upper motor functions measured by ARAT.

The pooled results from nine studies (Fig. 4a) using the Fugl-Meyer Assessment for Upper Extremity (FMA-UE), including 466 participants, demonstrated that game-based interventions did not yield statistically significant improvements in UE functions compared to control groups (SMD = 0.56, 95% CI:0.02–1.15, *p* = .06). The analysis exhibited considerable statistical heterogeneity ($I^{2}$ = 88%, *p* < .01), indicating substantial variability among the included studies. Subgroup analyses were conducted comparing 1) feedback versus no feedback in terms of neurorehabilitation principles, 2) stroke phase: chronic stroke versus acute or acute/subacute stroke, 3) training frequency, and 4) interventions delivered using consumer gaming system versus non-commercial/dedicated rehabilitation gaming platform/device. The findings are consistent with the primary analysis, where there were no statistically significant differences observed across the subgroup analyses (Supplementary Material Fig.S2 (a)-(d). Post-hoc sensitivity analyses were attempted to explore the effects by removing outliers (studies with high risk of bias/attrition rates). However, results remained similar to the primary and subgroup analyses (Supplementary Material Fig.S2 (e)-(h).

A random-effects meta-analysis of four studies (N = 470) examining UE function using the Action Research Arm Test (ARAT) yielded a pooled SMD of 0.15 (95% CI:0.22–0.52, *p* =.41), with substantial heterogeneity (*I^2^* = 73%, *p* = .01) (Fig. 4b). Sensitivity analysis excluding the sole study using immersive virtual reality (the other three studies using non-immersive VR) did not change the statistical significance of the result (Supplementary Material Fig. S3).

A narrative synthesis of five studies, which lacked sufficient data for meta-analysis, corroborated these findings. Studies reported no significant differences between game-based interventions and control groups in UE motor function, as measured by standardized assessments such as the FMA-UE, ARAT, and the Motor Function Test (MFT). Rémy-Néris et al. (2021) observed no significant between-group differences in FMA-UE (*p*=.22) and ARAT (*p* = .074) scores following a four-week intervention. Consistent findings were reported by Térémetz et al. (2022), with no significant group differences in FMA-UE (*p* = .43) and ARAT (*p* = .93) scores. Similarly, three additional studies reported no significant between-group differences in UE function following the interventions, as measured by the FMA-UE (Choi et al., 2014; Kuo et al., 2023; Long et al., 2020; Shin et al., 2015), or by MFT (Kim, 2018; Song and Lee, 2021). da Silva Ribeiro et al., 2015 reported that there were no significant differences between groups in FM scores across seven domains, including upper limb motor function where post-intervention scores were M = 44.7 (SD = 14.2) in intervention group versus M = 38.7(SD = 19.6) in control group, *p* = .486. Allegue et al. (2022) reported that both intervention and control groups had 50% (two out of four participants in each group) demonstrated an improvement change scores in FMA-UE, which was maintained over time. Similarly, Oh et al. (2019) reported that both intervention and control groups demonstrated significant improvements in FMA-UE. However, one study reported that the intervention group showed significant improvement in hand functional status compared to control group at post-intervention (*p* < .05) (Latif et al., 2025).

Song and Lee (2021) reported there was no statistically significant difference observed in UE sensory functions (two-point discrimination and stereognosis tests) in the affected arm between group at the end of the four weeks training, except for proprioception test which reported to be statistically significant different between intervention and control groups (*p* = .04).

Regarding real-world arm use, as assessed by the Motor Activity Log, no significant between-group differences were found by Adie et al. (2017) at a six-month follow-up or by Térémetz et al. (2022) at one-week post-therapy. In contrast, Allegue et al. (2022) reported that all participants in the intervention group exhibited improvement from baseline to post-intervention, compared to 80% improvement in the control group.

Muscle strength in the upper arm, as measured by the Motricity Index (MI), was also evaluated. Ali et al. (2024) found no significant differences between groups after two weeks of game-based intervention compared with task-based training [−2.5 (95%CI: −5.8, 0.8); Cohen’s d = 0.19; *p* = .143].

Analyses of hand function revealed some differential effects. Shin et al. 2016), employing the Jebsen–Taylor hand function test (JTT) and Purdue pegboard test (PPT), found that the intervention group demonstrated significant improvements in the JTT-total (F = 4.073, df = 1.497, *p* = .032) and JTT-gross (F = 4.155, df = 1.705, *p* = .025) scores, as reflected in significant Time × Group interactions. However, no significant differences between groups were observed in fine hand motor function (Shin et al., 2016). In terms of hand dexterity, two studies reported that the intervention group demonstrated significantly greater improvement in the Box and Block Test (BBT) scores when compared to the control group (Amin et al., 2024; Kuo et al., 2023).

## 4 Discussion

This review evaluated the characteristics and effectiveness of VR-based interventions on QoL and UE motor functions among stroke survivors. Thirty-four randomized controlled trials encompassing 1834 participants were included. The interventions varied in terms of setting, technology platforms, dose, and theoretical underpinnings. Our feasibility analyses indicated that VR-based interventions were generally acceptable and safe: participation among eligible patients typically exceeded 80%, attrition rates were usually below 20%, and adherence to the prescribed VR or game-based components was above 80% in the few trials that reported it (Bovim et al., 2025; Su et al., 2024). However, despite this encouraging feasibility profile, the effects on QoL and UE functions showed no significant advantage over conventional rehabilitation.

The meta-analysis revealed that VR-based interventions had a non-significant effect on QoL as measured by SIS, with inconsistencies observed across narrative synthesis findings. Our meta-analysis on QoL contrasts with a prior review reported by Domínguez-Téllez et al. (2020), which reported mixed outcomes for QoL, inconclusive results when assessed via the Modified Barthel Index (MBI), but favorable effects when measured with the Functional Independence Measure (FIM). However, it is important to note that neither the MBI nor the FIM is specifically designed to measure quality of life among stroke survivors, but can be used for patients with various conditions causing physical disability. One of the reasons for the non-significant effects could be the fact that our review conducted the pooled effects for pre- and post-intervention scores, which were of short duration. Furthermore, Bartlova et al. (2022) suggest that QoL is the worst among patients with the shortest time since their last stroke, whereas it improves with the time that has passed since the event.

Our findings suggest that VR interventions yield comparable outcomes to conventional therapy for improving UE functions in stroke survivors, with no significant difference between groups. This contrasts with the meta-analysis by Domínguez-Téllez et al. (2020), which reported favorable results of VR interventions on UE motor function (Fugl-Meyer Assessment for UE, standardized mean difference [SMD] = 1.53, 95% CI [0.51–2.54], *p* = .003). While their review pool had nine studies (year 2009–2016; sample sizes between 14 and 68 participants), the cited study is absent ‘Rubio (2016)’. Domínguez-Téllez et al. (2020) rated the included studies as methodologically robust using the PEDro scale. Further supporting the potential of VR, Wu et al. (2021) conducted a meta-analysis on six studies which reported to have satisfactory methodological quality and found that VR-based interventions significantly improved UE functions, measured by FMA-UE, with a large effect size (SMD 4.606, 95% CI: 2.733–6.479, *p* <.05), albeit with considerable heterogeneity (*I*^2^ = 92.045) (Wu et al., 2021). Similarly, Soleimani et al. (2024) conducted a review with 34 RCTs and demonstrated VR’s significant advantage over conventional occupational therapy (SMD 0.63, 95% CI 0.33–0.92, *p* <.001), though heterogeneity remained (*I*^2^ = 82%) (Soleimani et al., 2024). The divergence between our findings and prior reviews may stem from temporal shifts in conventional rehabilitation standards. Notably, previous reviews included some studies that were conducted more than a decade ago. Stroke rehabilitation guidelines have evolved to recommend higher amounts (Stinear et al., 2020). Additionally, most VR interventions in our review were non-immersive (with only semi-immersive and one fully immersive VR intervention), whereas Kiper et al. (2023) reported that fully immersive VR significantly improved overall UE functions as measured by FMA-UE (MD 6.33, 95% CI: 4.15–8.50, *I*^2^ = 25%, *p* <.001). This suggests immersion level critically influences outcomes, as fully immersive systems better simulate real-world tasks, thereby optimizing motor relearning.

Despite the meta-analyses of our intended outcomes revealing no statistically significant effects of VR-based interventions on QoL and UE functions, these findings do not automatically imply clinical irrelevance. Interpreting these findings in the context of minimal clinically important differences (MCIDs) will provide additional insights into whether the observed effect sizes are large enough to be perceived as meaningful for stroke survivors. For the QoL, the pooled standardized mean difference of -0.09 (favoring control group) fell below established MCID thresholds by Lin et al. (2010) for SIS physical domains. Authors reported that the mean change scores must reach 9.2 points on the strength subscale, 5.9 points on ADL/IADL, 4.5 points on mobility, and 17.8 points on hand function to be regarded as clinically important improvements. This indicates that the magnitude of benefit may remain below what patients perceive clinically meaningful, which may require substantial modification of the intervention protocols. Nonetheless, the different versions of SIS used across included studies may have contributed to heterogeneity in the pooled estimate. Future studies should standardize the SIS version.

For the UE functions, the pooled SMD of 0.56 approached statistical significance (*p* =.06) and exceeded commonly cited MCID thresholds for UE motor recovery in stroke rehabilitation. Page et al. (2012) estimated the clinical important difference for the UE Fugl Meyer to be 4.25 to 7.25 points in chronic stroke survivors using the ROC analysis, where the CID corresponds to an approximate SMD range of 0.25 to 0.60. Hence, the observed SMD of 0.56 falls within this range, suggesting that the effect, while imprecise due to wide confidence intervals and very low certainty evidence, may be clinically meaningful in some patients or settings. In contrast, the effect using ARAT (SMD = 0.15) fell below typical MCID thresholds for UE function. Lang et al. (2008) reported MCID values for the ARAT of 12–17 points in the early subacute phase after stroke, while Van der Lee et al. (2001) established that ARAT can detect a clinically relevant difference of 5.7 points. Thus, these raw score thresholds correspond to SMDs of approximately 0.2 to 0.6, suggesting that the magnitude of improvement observed in this review is unlikely to be clinically detectable.

Outcome measure heterogeneity further complicates the interpretation of the pooled estimates. Nine different QoL were employed across studies, with only six RCTs being eligible for SIS-based pooling. The FMA-UE and ARAT assess fundamentally different aspects of motor recovery, neurological impairment versus functional task performance, and their differential sensitivity to change may partly explain the discrepancy in effect sizes and the considerable heterogeneity observed (*I^2^* = 88% and *I^2^* = 73%, respectively). Future trials should adopt a consensus-based core outcome set to enable more reliable synthesis.

The null findings may also reflect insufficient intervention intensity. The most common duration was four weeks (n = 10), with sessions of 20–60 min delivered two to five times weekly, below high repetition, task specific volumes recommended by current stroke rehabilitation guidelines (Kwakkel et al., 2023; O’Flaherty and Ali, 2024). Furthermore, the predominance of non-immersive VR (n = 26 of 34 studies) may have limited sensorimotor engagement; Kiper et al. (2023) reported that fully immersive VR yielded significant FMA-UE improvements (MD = 6.33, 95% CI: 4.15–8.50, *I^2^* = 25%), suggesting that immersion level critically influences outcomes. Collectively, these observations suggest the null findings may reflect study design constraints rather than an inherent ceiling on the therapeutic potential of VR.

A notable challenge in interpreting our pooled estimates is the considerable statistical heterogeneity observed across UE outcomes. While we conducted sensitivity analyses to assess the robustness of the findings, the sources of this heterogeneity were not fully explored. Several factors are likely to have contributed to the observed variability. First, the included studies enrolled patients at different stages of stroke recovery (acute to chronic phases). Chronicity is known to influence the trajectory of spontaneous neurological recovery and the responsiveness to rehabilitation interventions (Langhorne et al., 2011; Stinear et al., 2020). Stroke survivors in the subacute phase may exhibit greater capacity for improvement due to the natural recovery processes, whereas those in the chronic phase may show more modest gains but potentially greater differentiation between intervention and control groups. Thus, the pooling of studies across these distinct recovery stages may have diluted or exaggerated the observed effect sizes, contributing to the high *I*^2^ values. Second, the intervention characteristics varied including total dose, session frequency, duration of intervention, and the nature of the VR systems used. As noted earlier, fully immersive systems appear to yield larger effects (Kiper et al., 2023), suggesting that technological immersion is a key moderator. However, only one study in this review employed fully immersive VR, limiting the ability to conduct subgroup analyses by immersion level. Third, the control interventions were not uniform across studies, introducing further variability into the pooled comparisons. This lack of standardization in both arms is a well-recognized source of heterogeneity in rehabilitation systematic reviews. For instance, Pollock et al. (2014) highlighted that evidence related to the dose of interventions is limited by substantial heterogeneity and noted that a lack of high-quality evidence prevents robust comparisons of interventions. Additionally, future reviews with larger numbers of studies should consider meta-regression to systematically examine these moderators and better understand the effectiveness of VR-based interventions on patient outcomes.

Beyond clinical effectiveness, our descriptive synthesis of safety data further supports the overall safety of game-based rehabilitation. Although adverse events were not the focus of our primary analyses, it is important to extract these data descriptively to address potential safety concerns that clinicians might have when considering VR-based interventions. Across the trials that systematically reported adverse events, serious events (such as recurrent stroke or unplanned hospitalization) occurred in both arms and were consistently judged to be unrelated to the VR or game-based components (Adie et al., 2017; Rémy-Néris et al., 2021). Where detailed information was available, most adverse events were mild and transient (typically musculoskeletal soreness, increased muscle tone, dizziness, or fatigue) and resolved after rest without lasting consequences (Kuo et al., 2023; Laffont et al., 2020; Lee et al., 2017). In some studies, the control groups experienced equal or greater numbers of such events compared with the VR groups (Laffont et al., 2020; Lee et al., 2017). Taken together, these data suggest that, when appropriately monitored, VR-based interventions have a safety profile comparable to that of conventional rehabilitation (Choi et al., 2016; Norouzi-Gheidari et al., 2021).

At the same time, several trials emphasized the importance of therapist guidance to ensure safe and effective use of game-based systems. Şimşek and Çekok (2016) reported that some patients using Nintendo Wii exhibited compensatory trunk and shoulder movements, postural imbalance, and increased muscle tone, leading the authors to recommend continuous physiotherapist supervision, particularly for individuals with persistent balance problems. Similarly, Lee et al. (2017) and Kuo et al. (2023) described how therapists adjusted task difficulty and posture in response to emerging soreness or dizziness. These observations highlight that VR-based training is not inherently “self-correcting”; without adequate supervision, there is a risk of reinforcing maladaptive movement patterns even in the absence of overt adverse events (Hung et al., 2014; Jandaghi et al., 2021). For home-based or minimally supervised programs, integrating initial supervised training, remote monitoring, and clear safety guidelines may therefore be essential (Proffitt and Lange, 2015; Winstein et al., 2016).

Among the 34 RCTs included in our review, only four were underpinned by theory, and only one of these contributed to the meta-analysis of QoL, and one to UE functions. This limited theoretical integration into intervention development represents a significant gap, given that theory-based interventions are critical for targeting the underlying mechanisms of behavior change (Michie et al., 2008). Furthermore, beyond ensuring design efficacy, theory-driven interventions facilitate theory testing, allowing refinement of behavioral models (Craig et al., 2008) and improving our understanding of the factors that influence outcomes (Nilsen, 2015). The scarcity of such theoretically informed trials in our review may explain the inconsistent findings in QoL and UE functions across studies.

### 4.1 Limitations

This review has several limitations that should be considered when interpreting the findings. First, the meta-analyses incorporated studies with considerable heterogeneity, particularly in intervention characteristics. This heterogeneity challenges the generalizability and comparison of the effects of the interventions. Second, the majority of included studies were rated as having either ‘some concerns’ or ‘high’ risk of bias in methodological quality. Consequently, with the overall certainty of evidence assessed using the GRADE criteria was rated as ‘low’ and ‘very low’ for outcomes (QoL and UE functions), limiting confidence in the pooled estimates. Third, our analyses focused on short-term outcomes (baseline and immediately post-intervention), which restricts the ability to evaluate long-term effects or the sustainability of benefits. Lastly, our search was restricted to articles published in English, which potentially introduces language bias by excluding relevant articles published in other languages.

### 4.2 Implications for practice and research

Although the pooled effects of VR-based interventions were not statistically significant, these interventions nevertheless show promise as a complementary approach to conventional rehabilitation, particularly for mitigating access barriers such as distance and transportation. Given that the overall certainty of evidence was rated as low and very low, the clinical implications of our findings must be interpreted with considerable caution. The pooled analyses did not demonstrate a significant advantage of VR-based interventions over conventional rehabilitation. As such, VR-based interventions are not a replacement for conventional therapy in routine stroke care. However, VR-based interventions may still hold value as a complementary or adjunctive option, particularly for stroke survivors who face barriers to accessing traditional rehabilitation. To maximize their impact, a collaborative, multidisciplinary approach should be adopted to integrate VR-based interventions, tailoring them to patients’ specific needs, preferences, and technological literacy.

Our feasibility findings, with recruitment generally exceeding 20% of eligible patients and withdrawal rates below 30% in most trials, are consistent with commonly applied feasibility benchmarks and further support the practicality of integrating VR-based programs into routine stroke care, provided that appropriate clinical supervision and follow-up are in place. Notably, adherence was only quantified in four trials, yet all reported high engagement with the VR or game-based components (83–95% of the prescribed dose), suggesting good usability among participants who initiated these programs, while also highlighting the need for more consistent reporting of adherence in future trials.

Our review also highlights critical research gaps. First, there is a need for more rigorous study designs, adequately powered RCTs with longer follow-up periods. Future trials should also stratify participants by stroke chronicity and standardize intervention protocols to reduce heterogeneity and enable more meaningful meta-analytic synthesis. Second, the optimal content and design of interventions in the included studies remain unclear due to high heterogeneity and poor evidence quality among existing studies, highlighting the necessity of incorporating patients’ perspectives into the development process to better tailor interventions to their needs. Additionally, most included studies lacked a theoretical framework to guide intervention development. Future research should integrate theoretical models to underpin the design of game-based interventions, thereby facilitating a deeper understanding of the causal determinants (e.g., motivation/adherence to intervention).

## 5 Conclusion

This review found that VR-based interventions do not confer superior benefits over conventional rehabilitation for improving QoL or UE functions in stroke survivors. The overall certainty of the evidence was rated as low to very low, substantially limiting confidence in the estimated effects. Consistent with this, the review identified several critical gaps in the literature, including methodological heterogeneity, limited use of theoretical frameworks, and a lack of long-term follow-up data. These gaps underscore the need for rigorously designed, adequately powered RCTs with greater theoretical underpinning. Although our descriptive synthesis suggested that these interventions are generally feasible and safe, this finding is based on limited reported data, particularly for adherence which was quantified in only a minority of RCTs. As such, the lack of demonstrable clinical effectiveness indicates that VR-based rehabilitation, in its current form should not replace conventional rehabilitation, but may serve as a complementary or adjunctive option, particularly when access to traditional rehabilitation is limited.

## Data availability

The data underlying this article are available in the article and in its online supplementary material.

## Funding

This work was supported by the NUS Research Fellow Start Up Grant (WBS No. A-0010310-00-00). Dr. Batalik is supported by the Ministry of Health of the Czech Republic - conceptual development of research organization (FNBr, 65269705).

## CRediT authorship contribution statement

**Ranee Reesa Tan:** Writing – review & editing, Writing – original draft, Methodology, Formal analysis, Data curation, Conceptualization. **Sok Ying Liaw:** Writing – review & editing, Supervision, Conceptualization. **Yasmine Yan Qi Thng:** Methodology, Data curation. **Suebsarn Ruksakulpiwat:** Writing – review & editing. **Ladislav Batalik:** Writing – review & editing, Writing – original draft. **Mei Sin Chong:** Writing – review & editing, Writing – original draft, Supervision, Methodology, Formal analysis, Conceptualization.

## Declaration of competing interest

All authors declare no competing interests.

## Appendix Supplementary materials

Image, application 1
