# Supplementary material for: Effectiveness of virtual reality-based rehabilitation on quality of life and upper extremity functions among stroke survivors: A systematic review and meta-analysis
Source: Int J Nurs Stud Adv. 2026 Jul 12;11:100629. doi: 10.1016/j.ijnsa.2026.100629 (PMC13427661; doi:10.1016/j.ijnsa.2026.100629)
Supplement: Supplementary file 1 [file mmc1.docx]

**Effectiveness of virtual reality-based rehabilitation on quality of life and upper extremity functions among stroke survivors: A systematic review and meta-analysis.**

**Background:** Stroke rehabilitation faces challenges such as limited accessibility and engagement, prompting interest in virtual reality-based interventions. While virtual reality shows promise, its effectiveness in improving quality of life (QoL) and upper extremity (UE) functions in stroke survivors remains unclear.

**Objectives:** To evaluate (1) the effectiveness of virtual reality-based interventions in QoL and UE functions among stroke survivors and (2) the feasibility and safety indicators across trials.

**Data Sources:** A comprehensive search was conducted across six electronic databases: PubMed, EMBASE, Cochrane, Scopus, PsycINFO, and CINAHL to identify relevant articles published between Jan 2014 and December 2024. The search was updated on 9 May 2026.

**Review Methods:** The Cochrane Handbook for Systematic Reviews of Interventions and the Preferred Reporting Items for Systematic Reviews and Meta-Analyses (PRISMA) guidelines were employed to ensure methodological rigor and standardized reporting. Two independent reviewers assessed the eligibility of the articles, and the quality of the studies were appraised using the Revised Cochrane Risk of Bias Assessment Tool for Randomized Trials (RoB2). Feasibility indicators (participation, adherence, attrition) and adverse events were also extracted.

**Results:** Thirty-four randomized controlled trials involving 1,834 participants were included. Two studies had a low risk of bias, 19 had some concerns, and 13 had a high risk. Meta-analyses revealed non-significant effects of virtual reality-based interventions on QoL (SMD = –0.09, 95% CI –0.30 to 0.11, *p* = .36; low certainty) and UE functions assessed by upper extremity Fugl-Meyer (SMD = 0.56, 95% CI -0.02 to 1.15, *p* = .06; very low certainty) and Action Research Arm Test (SMD = 0.15, 95% CI -0.22 to 0,52, *p* = .41; low certainty). Narrative synthesis yielded mixed findings. Feasibility outcomes were generally favorable: participation among eligible patients was typically >80%, attrition <20%, and adherence exceeded 80% in the few trials reporting it. Adverse events were mainly mild and comparable to those in control groups.

**Conclusion:** Current evidence does not demonstrate the superiority of virtual reality-based interventions over conventional rehabilitation for improving QoL or UE functions in stroke survivors, despite generally favorable feasibility and safety profiles. The low-to-very low certainty of evidence highlights the need for stronger methodological rigor, theoretical frameworks, and long-term follow-up. Virtual reality may serve as a complementary option when access to traditional rehabilitation is limited.

**Registration:** PROSPERO (CRD42024619427).

**Keywords:** virtual reality, rehabilitation, upper extremity, quality of life, stroke, feasibility, exergames

**Highlights**

What is already known?

- Virtual reality rehabilitation interventions which are game-based, are increasingly adopted and generally considered feasible and safe, but existing systematic reviews have reported mixed results regarding their efficacy.
- Conventional post-stroke rehabilitation is often limited by challenges related to access and adherence.

What this paper adds?

- This review found that virtual reality-based rehabilitation did not significantly improve quality of life and upper extremity functions compared to usual care or no intervention.
- This review also confirms the favorable feasibility of such interventions, demonstrating high participation (> 80%) and adherence (> 80%), low attrition (< 20%), and a mild adverse event profile comparable to control groups.

**1. Introduction**

Globally, the prevalence of stroke has escalated to over 100 million people, with an annual incidence of 12.2 million new strokes (World Stroke Organization, 2022). This trend is expected to worsen, driven by the rising prevalence of chronic diseases and an aging population (Béjot & Yaffe, 2019; Yousufuddin & Young, 2019). Stroke imposes substantial economic burdens on healthcare systems globally, particularly in post-stroke care. For instance, the highest costs of post-stroke care in outpatient settings were reported in the US, with a mean expenditure of $1,236 per patient per month, followed by the UK at $1,039 (Rajsic et al., 2019). Furthermore, post-stroke depression is a prevalent complication, affecting between 18% and 33% of stroke survivors, and is associated with poor patient outcomes (Medeiros et al., 2020). Approximately 50 and 75% of stroke survivors experience persistent motor impairment in their affected upper extremities (Anwer et al., 2022). As a result, many face challenges in performing activities of daily living. Wondergem et al. (2017) found that up to 40% of stroke survivors experience a deterioration in their ability to carry out daily tasks over time, often leading to functional dependence. The concomitant reduction in activities of daily living and the presence of post-stroke depression may reduce quality of life (QoL) (Li et al., 2022).

Given the complex nature and impacts of stroke, the needs of stroke survivors are often unique (Hawkins et al., 2017). Stroke rehabilitation initiated after 24 hours of a stroke is deemed safe (Coleman et al., 2017). According to Vazquez-Guimaraens et al. (2021), most studies indicate that neurological recovery predominantly occurs within the first three months, with optimal recovery observed in the initial four to six weeks. Neuroplasticity, the brain’s ability to adapt and reorganize neural networks through repetitive experiences (Puderbaugh & Emmady, 2023), is vital for regaining motor and cognitive functions after a stroke (Aderinto et al., 2023). Conventional rehabilitation typically includes physiotherapy (PT), occupational therapy (OT), and speech therapy (ST), tailored to the nature and severity of stroke impairment (Nair & Taly, 2002). Outpatient stroke therapy sessions typically involved patients attending two to three days per week, with each day consisting of a single session that averaged 36 ± 14 minutes in duration (Lang et al., 2009). Despite the critical importance of stroke rehabilitation, participation and adherence rates have consistently been challenging (Miller et al., 2017). Conventional rehabilitation frequently presents significant challenges, including limited accessibility due to geographical disparities and the absence of individualized rehabilitation plans, which can hinder optimal patient outcomes (Li et al., 2024).

To overcome the challenges associated with conventional stroke rehabilitation, technological rehabilitation programs have been increasingly adopted. The programs not only accommodate the mobility limitations of stroke patients but also facilitate home-based rehabilitation, thereby minimizing inconvenience. Among these technologies, two related but distinct approaches have gained prominence including game-based rehabilitation and virtual reality (VR)-based rehabilitation. Game-based rehabilitation refers to interventions that incorporate game-like elements such as meaningful play, goals, feedback, rewards, or challenges to enhance patient engagement and motivation during therapy (Barrett et al., 2016). VR-based rehabilitation uses computer-generated environments to simulate real-world or imaginary scenarios for therapeutic purposes (Laver et al., 2011). Importantly, these two concepts are not mutually exclusion, with many VR-based interventions incorporate game-like features (Lohse et al.2014), and game-based interventions can be delivered using VR. For the purposes of this review, both VR-based and game-based interventions are considered, recognizing their shared therapeutic goals.

The feasibility and effectiveness of game-based intervention protocols for stroke rehabilitation have been well-documented, offering engaging experiences (Tosto-Mancuso et al., 2022). Additionally, devices deployed in game-based interventions provide significant flexibility and adaptability to meet individual patient needs (Baranyi, 2023). Systematic reviews have been conducted on game-based rehabilitation for post-stroke patients. Saeedi et al. (2021) focused on physical rehabilitation, identifying key components including balancing training, limb mobilization, and muscle strengthening. This review reported that VR-based approaches and Nintendo Wii Fit games were most used, with a particular focus on limb movement and balance training. A more recent systematic review by Sanchez-Gil et al. (2025) examined the devices used in game-based rehabilitation and their impact on emotional, personal, and social aspects of stroke survivors. The authors highlighted that gamified devices improved motor and cognitive function and had a significant positive impact on emotional, social and personal levels of stroke survivors. In contrast to these broad systematic reviews, existing meta-analyses have primarily focused on game-based rehabilitation for the upper extremities post-stroke (Karamians et al., 2020; Wang et al., 2022). Across the 38 studies included in the review by Karamians et al. (2020), participants in the VR or gaming intervention groups achieved a mean improvement of 28.5% of the maximum possible score on the outcome measures (Wolf Motor Functioning Test, the Fugl-Meyer, or the Action Research Arm Test). Wang et al. (2020) included only non-immersive device rehabilitation modes and reported that these interventions significantly improved upper limb motor function and hand dexterity compared to conventional therapy.

With the rapid advancement of technologies, VR has emerged as a promising tool within game-based rehabilitation programs, enabling patients to engage with simulated environments that resemble real-life scenarios and facilitating interactive therapeutic experiences. VR devices can be classified by the level of immersion experienced by the user: 1) non-immersive VR, which uses standard computers or gaming consoles (e.g., mouse, keyboard, joystick), allowing users to experience virtual environment while fully aware of their real surroundings; 2) semi-immersive VR involves projecting the virtual environment onto large screens and employs advanced devices (e.g., cybergloves, motion sensors), offering partial immersion but not complete separation from the real world; and 3) immersive VR uses a head-mounted display and a 3D input device, allowing users to fully engaged with the virtual environment while remaining disconnected with the real world (Salatino et al., 2023).

Despite this promising evidence, it remains unclear which specific characteristics of virtual reality rehabilitation most significantly affect QoL and motor functions in stroke survivors. Therefore, this present review aimed to evaluate the effectiveness of VR rehabilitation on QoL and UE functions among stroke survivors undergoing rehabilitation. In addition, we aimed to describe the feasibility and safety indicators across trials.

**2. Methods**

This review was conducted in accordance with the Cochrane Handbook for Systematic Reviews of Interventions and the Preferred Reporting Items for Systematic reviews and Meta-Analyses (PRISMA) guidelines (Page et al., 2021) (Supplementary Material: Table S1). The study protocol has been registered in the International Prospective Register of Systematic Reviews (PROSPERO) (CRD42024619427).

*2.1 Eligibility criteria*

*2.1.1 Inclusion criteria*

Randomized controlled trials (RCTs) utilizing VR-based interventions aimed at improving QoL and motor functions were deemed eligible for inclusion in this review. The review was guided by a PICO (population, intervention, comparison, outcome, study design) search strategy, which was employed to formulate review questions and direct comprehensive literature searches.

*2.1.1.1 Population*

Patients who were clinically diagnosed with stroke (ischemic or hemorrhagic), aged 18 years and over, able to provide informed consent, and who understood instructions were included. Exclusion criteria include participants with post-stroke complications such as visual or auditory impairment, vestibular dysfunction, aphasia, or cognitive disabilities due to other neurological conditions.

*2.1.1.2 Intervention*

Studies with any form of VR-based interventions were considered, regardless of delivery mode, and whether provided as a standalone or in combination with conventional rehabilitation.

*2.1.1.3 Comparison*

Studies with any type of control, such as no intervention or conventional rehabilitation (usual care), were included.

*2.1.1.4 Outcomes*

The primary outcome included QoL with any use of generic or stroke-specific QoL assessment tools. Meanwhile, the secondary outcome was the upper extremity functions. Feasibility and safety indicators, including participants rates, adherence, attrition, and adverse events were extracted as descriptive outcomes of interest to contextualize the primary and secondary outcomes.

*2.2 Search strategy*

A comprehensive search was conducted across six electronic databases (PubMed, EMBASE, Cochrane, Scopus, PsycINFO, CINAHL) to retrieve relevant published literature. The ProQuest Theses and Dissertations and Google Scholar were employed to access unpublished and grey literature. The search utilized a combination of keywords and MeSH terms, including ‘virtual reality’, ‘exergaming’, ‘gamification’, ‘stroke’, and ‘quality of life’, along with Boolean operators such as ‘AND’ and ‘OR’ as appropriate. The search was conducted for studies published between January 2014 and December 2024. This 10-year limit was applied due to the rapid pace of technological advancement, ensuring the review captured current and relevant interventions (Xu et al., 2021). The search was updated on 9 May 2026. Only English-language articles were considered, and a librarian was consulted to optimize the search strategies. Further details on the search strategies can be found in Supplementary Material: Table S2.

*2.3 Selection process*

The retrieved articles were imported into the EndNote 21 software for organization and management, with duplicate articles removed using the functionality of the software. Two reviewers (R.T.R. and Y.T.) independently screened the titles and abstracts against the predetermined eligibility criteria to identify studies for full-text review. Any disagreements were resolved by consulting a third reviewer (C.M.S).

*2.4 Data extraction*

Data extraction was independently conducted by two reviewers (R.T.R and C.M.S) using a custom-developed data extraction form, adapted from the Cochrane Data collection Form for Randomized Controlled Trials (Higgins et al., 2024). The extracted information encompassed the following details: authors, publication year, country of study, sample size, participant characteristics, stroke (ischemic or hemorrhagic), specifics of the intervention and control groups, outcome measurement tools, results, and attrition rate. In addition, we extracted indicators of trial feasibility and safety, including the numbers of participants screened, eligible, randomized and analyzed, attrition rates, adherence or engagement with the intervention and control conditions where reported, and adverse events (type and severity). These indicators were not predefined as formal outcomes for meta-analysis. Rather, they were extracted as descriptive outcomes of interest, informing the interpretation of the effectiveness data and providing practical guidance for stake holders of VR-based interventions in stroke rehabilitation settings.

*2.5 Quality assessment and certainty of evidence*

Quality appraisals were independently conducted by two reviewers (R.T.R. and Y.T.) using the revised Cochrane Risk of Bias for Randomized Trials tool (RoB 2), which assesses five domains: 1) bias arising from the randomization process, 2) bias due to deviations from intended interventions, 3) bias due to missing outcome data, 4) bias in measurement of the outcome, and 5) bias in selection of the reported result (Sterne et al., 2019). The overall risk-of-bias judgements were categorized as having low, some concerns, or high risk of bias. The Grading of Recommendations, Assessment, Development, and Evaluation (GRADE) framework was employed to determine the confidence in the quality of evidence of each outcome, considering factors such as study design, risk of bias, inconsistency, indirectness, and imprecision, and was rated as high, moderate, low or very low (Guyatt et al., 2011). The GRADEpro software was utilized to create a summary of the evidence table (GRADEpro GDT, 2025). Any disagreements were discussed with the third reviewer (C.M.S.).

*2.6 Data Synthesis and Statistical Analyses*

Meta-analyses were performed using the Review Manager version 8.13.0. (RevMan, 2024). With a combination of results from two or more separate studies, the inverse-variance random effects model was used to analyze pooled effect size for studies, as it provides a better fit for actual sampling distribution (Borenstein et al., 2010). Studies with data presented as median and interquartile range (IQR) were converted to means and standard deviations (SDs) using formulas from Shi et al. (2023), Luo et al. (2018), and Wan et al. (2014). Mean difference (MD) was used for outcomes measured on the same scale, while standardized mean difference (SMD) was used for outcomes measured on different scales. The effect size was interpreted using the Cohen’s *d* effect size, categorized as small (*d* < 0.4), moderate (0.4 ≤ d < 0.8) and large (d ≥ 0.8) (Schünemann et al., 2024). Heterogeneity was assessed using Chi-square ($\chi^{2}$) and $I^{2}$ statistics, with a significance level of *p* < 0.10 (Higgins & Thomas, 2024). The $I^{2}$ value was interpreted as indicating low heterogeneity (≤ 30%), moderate heterogeneity (31% - 50%), substantial heterogeneity (51% - 75%), and considerable heterogeneity (> 75%) (Higgins & Thomas, 2024).

Sensitivity analysis, a process to evaluate the robustness of the findings, was conducted for analysis with substantial or considerable heterogeneity, by substituting alternative decisions or value ranges for those that were unclear (Higgins & Thomas, 2024). Subgroup analyses were conducted to assess whether variations in QoL were influenced by different game elements and to explore potential causes of heterogeneity (Higgins & Thomas, 2024). When statistical pooling for meta-analysis was not feasible, a narrative synthesis or synthesis without meta-analysis (SwiM) approach was employed to qualitatively describe the reported data from the studies (Campbell et al., 2020).

**3. Results**

*3.1 Study selection*

A total of 1,764 articles were retrieved from the six electronic bibliographic databases. The detailed study screening and selection process is illustrated in the PRISMA flow diagram (Fig. 1). After the removal of duplicates (n = 185), 1,579 articles were excluded following the screening of their titles and abstracts, resulting in 66 articles for retrieval. Of these, 13 articles could not be retrieved due to unavailability of the full text. A total of 53 articles underwent full-text screening. An additional 1,675 articles were identified through grey literature. Ultimately, 34 randomized controlled trials (36 reports) were included in this review. Supplementary Material: Table S3 (Supplementary Material) presents a comprehensive list of studies excluded during the full-text screening phase, along with specific exclusion reasons.

[Insert Figure 1]

Fig. 1. PRISMA Flow diagram.

*3.2 Characteristics of included studies*

Table 1 summarizes the characteristics of the studies included in this review. A total of 1,834 participants were involved in 34 RCTs (36 reports), with 928 adults in the intervention group and 906 in the control group. The studies were conducted across various countries: Brazil (n = 1), Canada (n = 1), China (n = 1), Czech Republic (n = 1), France (n = 3), Hong Kong (n = 1), India (n = 2), Indonesia (n = 1), Netherlands (n = 1), Pakistan (n = 2), Portugal (n = 1), Spain (n = 3), South Korea (n = 9), Switzerland (n = 1), Taiwan (n = 2), Turkey (n = 3), and the United Kingdom (n = 1). Sample sizes varied, ranging from as small as 10 participants (Song & Lee, 2021) to as large as 235 participants (Adie et al., 2017). The intervention duration ranged between two weeks (Ali et al., 2024) to 12 weeks (Huber et al., 2025; Kim, 2018). Attrition rates across studies ranged between 0% (Amin et al., 2024; Choi et al., 2014; da Silva Ribeiro et al., 2015; Faria et al., 2016; Kim, 2018; Latif et al., 2025; Marques-Sule et al., 2021; Park et al., 2021; Velmurugan et al., 2023) to 50% (Shin et al., 2016). Control groups in all included studies received usual care, which typically comprised standard exercise therapy or conventional therapy (i.e., physiotherapy and occupational therapy). In addition to usual rehabilitation, one study had participant-tailored arm exercises (Adie et al., 2017), Ali et al. (2024) provided upper limb task-based training. One study with active control with a motor relearning program (Velmurugan et al., 2023).

Table 1. Summary of the included studies (N=34)

| Author (year) Country | Study design | Participants a) Sample size b) Mean age  c) Female  d) Type of stroke  e) Phase of stroke (acute/subacute/ chronic) | Study setting/ Provider | Intervention a) setting b) intervention provider c) description of the intervention d) dose e) other information | Control a) setting b) description of the intervention c) dose d) other information | Data collection time points Outcome(s)/measurement tools | a) Findings b) Attrition |
| --- | --- | --- | --- | --- | --- | --- | --- |
| Adie et al (2017) United Kingdom | Multicenter,  pragmatic, parallel group, RCT | a) N = 235  IG = 117, CG = 118  b) Mean age = 67.3 ± 13.4  IG: mean age = 66.8 ± 14.6  CG: mean age = 68.0 ± 11.9  c) IG: Female = 51  CG: Female = 53  d) IG: ischemic stroke = 104, hemorrhagic stroke = 13 CG: ischemic stroke = 105, hemorrhagic stroke = 13  e) Acute/  subacute stroke survivors | Home-based/ Research therapist | a) Home-based b) A research therapist c) Warm-up exercise for 15 min, followed by intervention. Wii sports games (bowling, tennis, golf and baseball). d) Dose: 1) Amount = 45 min 2) Frequency = daily 3) Duration = 6 weeks e) Daily diary, weekly telephone follow-up, usual rehabilitation therapy by local stroke team. | a) Home-based b) A research therapist c) Warm-up exercise for 15 min, followed by tailored arm exercises. Participant-tailored arm exercises (based on the Graded Repetitive Arm Supplementary Program). d) Dose: 1) Amount = 45 min 2) Frequency = daily 3) Duration = 6 weeks e) Daily diary, weekly telephone follow-up, usual rehabilitation therapy by local stroke team. | Baseline, 6 weeks, 6 months  Primary outcome:  1) ARAT Secondary outcome:  1) Canadian Occupational Performance Measure 2) SIS 3) mRS 4) EQ-5D 3L 5) MAL-14*   * only measured at 6 months | a) No significant differences for all outcomes b) 12.1%* |
| Ali et al (2024) India | Multi-centered, single-blinded, RCT | a) N = 120  IG = 64, CG = 56  b) IG: mean age = 54.4±11.7  CG: mean age = 57.7±10.9  c) IG: Female = 14  CG: Female = 15  d) IG: ischemic stroke = 53, hemorrhagic stroke = 11 CG: ischemic stroke = 47, hemorrhagic stroke = 9  e) Acute/subacute stroke survivors | Rehabilitation center / physical therapist | a) Rehabilitation centers b) Physical therapist c) Received a gamified upper limb program (functional games) using the ArmAble device + conventional therapy. d) Dose: 1) Amount = 45-60 min/day 2) Frequency = 6 days/week 3) Duration = 2 weeks e) Received conventional therapy. For post-intervention - Received a functional upper limb rehabilitation home-based program - 30 min/day for 6 days/week for 4 weeks - Logbook - Weekly telephone follow-up | a) Rehabilitation centers b) Physical therapist c) Received upper limb task-based training + conventional therapy d) Dose: Amount = 45-60 min/day Frequency = 6 days Duration = 2 weeks e) Conventional therapy consisted of  facilitatory/inhibitory techniques, strength training, passive and active limb mobilization, balance, and ambulatory training - 45 to 60 min/day For post-intervention - Received a functional upper limb rehabilitation home-based program - 30 min/day for 6 days/week for 4 weeks - Logbook - Weekly telephone follow-up | Baseline, 2nd week (immediate post-intervention), 6th week (follow-up assessment) Primary outcomes: 1) Upper limb function and activity - FM-UE and ARAT Secondary outcomes: 1) upper limb strength - motricity index (MI)-arm  2) degree of disability - mRS  3) Impact of the consequences of stroke - SIS-V 3.0  * game user experience - game user experience satisfaction scale (GUESS) only for IG at 2nd week | a) At 2 weeks post-intervention, a statistically significant improvement was observed for IG in the FM-UE (*p* =.003).  At 6 weeks, FM-UE and ARAT scores were significantly higher in the IG than in the CG (*p* = .003 and *p* = .046, respectively). No significant differences were found in other outcomes.  Adherence to exercise training at home during the 4-week period was 89.1% in the IG and 85.8% in the CG. b) 18.3% |
| Allegue et al (2022) Canada | 2-arm feasibility clinical trial | a) N = 11  IG = 6, CG = 5  b) IG*: mean age = 57.8 ± 21.8  CG^ϯ^: mean age = 56.4 ± 17.3  c) IG: Female = 2  CG: Female: 3  d) IG: ischemic stroke = 2, hemorrhagic stroke = 1 (1 participant in the IG had an unspecified type of stroke)  CG^ϯ^: ischemic stroke = 3, hemorrhagic stroke = 2   e) Chronic stroke survivors  * data from 4 participants  ^ϯ^ data from 5 participants | Home-based / remote control by clinician | a) Home-based b) Clinician c) VirTele program: Before intervention - 1-hour training session to familiarize with the Jintronix exergames (5 games) A) Jintronix exergames - upper extremity rehabilitation  d) Dose: 1) Amount = 30 min/session 2) Frequency = 5 days/week 3) Duration = 8 weeks e) Reacts app - videoconference sessions with clinicians synchronized with sessions when the participant was playing exergames, used by the clinician to provide motivational interviewing to the participant | a) Home-based b) Participants were offered one session with a clinician c) Usual care: Graded Repetitive Arm Supplementary Program (GRASP) - home rehabilitation training program that included exercises for the arm and hand and functional activities targeting the upper extremity.  d) Dose: 1) Amount = 30 min/session 2) Frequency = 5 days/week 3) Duration 8 weeks | Baseline, 8 weeks (post-intervention), 12 weeks (1 month post-intervention), 16 weeks (2 months post-interventions)  Primary outcome: 1) FMA-UE Secondary outcomes: 1) MAL-30  2) SIS-16 3) TSRQ-15 | a) FMA-UE and Motor Activity Log-30 - IG and CG demonstrated an improvement in >50% of the participants, from 1 month post-intervention to 2 months post-intervention.  SIS-16 scores - CG reported improvement in activities of daily life (3/5, 60%), hand function (5/5, 100%), and mobility (2/5, 40%), from baseline to 2 months post-intervention.  TSRQ-15 - 75% of the participants in IG demonstrated an increase in the autonomous motivation score from baseline to immediate post-intervention. b) 18.2% |
| Amin et al (2024) Pakistan | Single-centered, RCT | a) N = 52  IG = 26, CG = 26  b) IG: mean age = 51.8 ± 12.9  CG: mean age = 49.8 ± 9.9  c) IG: Female = 10 CG: Female = 8  d) Type of stroke not mentioned  e) Subacute stroke survivors | Physiotherapy department / physical therapist | a) Physiotherapy department b) Physical therapist c) VR based game intervention (immersive VR settings with VR headset) plus conventional physical therapy.  Games were developed with the Unity3D game engine and run by the Android Package Kit (APK file) on the Oculus Quest 2 Virtual Reality device. d) Dose: 1) Amount = 24 min of VR hand games and 24 min of therapy (first 2 weeks); 40 min of VR hand games and 40 min of conventional therapy (next 4 weeks) 2) Frequency = 4 days/week 3) Duration = 6 weeks | a) Physiotherapy department b) Physical therapist c) Conventional physical therapy: Range of Motion (ROM), stretching, resistance, and strengthening exercises. d) Dose: 1) Amount = 48 min (Week 1 and Week 2); 80 min (for the next 4 weeks) 2) Frequency = 4days/week 3) Duration = 6 weeks | Baseline, Week 4, Week 6, Week 9  Primary outcomes: 1) FMA-UE 2) ARAT 3) Hand dexterity - BBT  Secondary outcomes: 1) Capacity to carry out instrumental and everyday activities - MBI* 2) Quality of life - SSQOL* *Only assessed at baseline and Week 9 | a) IG demonstrated significantly greater scores in FMA-UE, ARAT, and BBT at week 4, 6, and 9 when compared with CG (*p* <.005). IG demonstrated significantly greater scores in MBI and SSQOL when compared with CG (*p* <.001)  b) 0% |
| Cano-Mañas et al (2020) Spain | Single-centered, RCT | a) N = 56  IG = 28, CG = 28  b) Mean age = 63.13 ± 10.38 IG: mean age = 60.35 ± 9.84  CG: 65.68 ± 10.39  c) IG: Female = 11  CG: Female = 14  d) IG: ischemic stroke = 17, hemorrhagic stroke = 6 CG: 14, ischemic stroke = 15, hemorrhagic stroke = 10   e) Subacute stroke survivors | Hospital / physical therapist | a) Hospital b) Physical therapists c) Video game-based therapy using Xbox 360° video games console and the Kinect (20 min) plus conventional rehabilitation (35 min of physical therapy + 35 min of occupational therapy) d) Dose (for intervention): 1) Amount = 20 min 2) Frequency = 3 times/week 3) Duration = 8 weeks e) Otherwise followed the conventional rehabilitation schedule | a) Hospital b) Physical therapists c) Conventional rehabilitation - task-oriented motor training d) Dose: 1) Amount = 45 min of physical therapy and 45 min of occupational therapy 2) Frequency = 5 times/week 3) Duration = 8 weeks | Baseline, 8 weeks  Outcomes: 1) mRS 2) BI 3) Tinetti Scale for Balance and Gait 4) Functional Reach Test 5) Get UP and Go Test 6) Baropodometry 7) EuroQoL 5D (5Q-5D) 8) Self-developed scale on satisfaction, adherence, and motivation with the treatment of video-game based therapy. | a) IG reported statistically higher mRS (*p* <.001), BI (*p* =.005), Tinetti gait assessment (*p*=.002), Functional Reach Test (*p* <.001), Get Up and Go Test (*p* =.005), EQ-5D [pain/discomfort dimension (*p* <.001), anxiety/depression dimension (*p* <.001)], VAS (*p* <.001). b) 14.3% |
| Choi et al. (2014)  South Korea | Single-blind RCT | a) N = 20  IG = 10, CG = 10  b) IG: mean age = 64.30 ± 10.3  CG: mean age = 64.70 ± 11.3  c) IG: Female = 5  CG: Female = 5  d) IG: ischemic stroke = 8, hemorrhagic stroke = 2 CG: ischemic stroke = 6, hemorrhagic stroke = 4   e) Acute/ subacute stroke survivors (had a stroke within the last 90 days) | Hospital/  Occupational therapist | a) Department of rehabilitation b) Occupational therapist c) Commercial gaming based VR movement therapy using the Nintendo that consisted games like swordplay, table tennis, and canoe games. d) Dose (for intervention): 1) Amount = 30 min 2) Frequency = 5 times/week 3) Duration = 4 weeks e) Participants also received conventional rehabilitation therapy | a) Department of rehabilitation b) Occupational therapist c) Conventional occupational therapy that included stretching and strengthening exercises (full range of motion of the upper extremity) and chose appropriate tasks for each participant and developed them in stages. d) Dose: 1) Amount = 30 min 2) Frequency = 5 times/week 3) Duration = 4 weeks  e) Participants also received conventional rehabilitation therapy | Baseline, at the end of the 4-week intervention  Primary outcome:  FMA-UL  Secondary outcomes:  1) MFT  2) BBT  3) Grip strength was evaluated using a dynamometer  4) K-MMSE  5) Visual and auditory continuous performance tests (CPTs) to assess attention  6) K-MBI | a) There were no statistically significant differences between groups in FMA-UL (*p* = .63), MFT (*p* = .48), BBT (*p* = .43), grip strength (*p* = .53), K-MMSE (*p* = .22), CPT (*p*-values ranged from 0.12-0.85), and K-MBI (*p* = .53).  b) 0% |
| Dabrowska et al. (2025)  Czech Republic  Dabrowska et al. (2023)  *Another report | RCT with a parallel group | a) N = 70  IG = 35, CG = 35  b) IG: mean age = 59.4 ± 8.9  CG: mean age = 63.0 ± 8.8  c) IG: Female = 12  CG: Female = 12  d) Type of stroke not mentioned   e) Acute/subacute stroke survivors | Rehabilitation sanatorium/  Physiotherapist or occupational therapist | a) Rehabilitation center b) Physiotherapist or occupational therapist c) VR therapy using the Oculus Quest 2 and VITALIS Pro VR (3 times/week) plus conventional rehabilitation.  A total of four programs: 1) free painting; 2) 2D tracing, 3) 3D painting, and 4) puzzle. Each task has three types of environments: 1) forest; 2) space; and 3) sea. d) Dose (for intervention): 1) Amount = 30 min/session 2) Frequency = 3 times/week 3) Duration = 4-5 weeks | a) Rehabilitation centre b) Conventional therapy – individual physical therapy, occupational therapy, and miscellaneous therapies (e.g., iodobromine bath and wrap, oxygen therapy, etc.) d) Dose: 1) Amount = 30 min of physical therapy, 30 min of occupational therapy 2) Frequency = 2 times/week 3) Duration = 4-5 weeks | Baseline, immediately post intervention, 4 weeks post intervention, and one year post intervention.  Outcomes:  1) MMSE  2) BI  3) EBI  4) WHODAS 2.0 | a) No significant differences were found between IG and CG in any of the outcomes.  b) 28.6% (post intervention) and 74.3% (1 year post intervention) |
| da Silva Ribeiro et al (2015) Brazil | Single-blind RCT | a) N = 30  IG = 15, CG = 15  b) IG: mean age = 52.8 ± 8.6 CG: mean age = 53.7 ± 6.1  c) IG: Female = 10  CG: Female = 9  d) Type of stroke not mentioned  e) Chronic stroke | Not specified / Physical therapists | a) Not specified b) Physical therapist c) Virtual rehabilitation via Nintendo Wii: Received an intervention in a 20 m2 room equipped with the Nintendo Wii and a multimedia projector. Started with a 10-min stretching including upper and lower limbs, and trunk muscles. 50-min protocol of Nintendo Wii games with 1 min rest interval between each game.   d) Dose:  1) Amount:1h (50 mins Nintendo Wii games) 2) Frequency = Twice/week 3) Duration = 8 weeks | a) Not specified b) Physical therapist c) Conventional physical therapy: including activities such as stretching, active-resisted mobilization of the trunk, straightening, balancing, gripping activities, scapular mobilization, active/active-assisted diagonal movement of the upper limbs  d) Dose: 1) Amount = 60 mins/session 2) Frequency = Twice/week 3) Duration = 8 weeks | Baseline, 8 weeks (post-intervention)  Outcomes: 1) SF-36 2) Fugl–Meyer (FM) | a) IG reported to have significantly higher SF-36 scores in physical functioning post-intervention when compared with CG (*p* <.001). No other significant differences were observed. b) 0% |
| de Rooij et al. (2022) Netherlands | Double-blinded RCT with two parallel groups | a) N = 55  IG = 28, CG = 27  b) IG: mean age = 65 (57–70)  CG: mean age = 61 (53–71)  c) IG: Female = 10  CG: Female = 6  d) IG: ischemic stroke = 24, hemorrhagic stroke = 4  CG: ischemic stroke = 20, hemorrhagic stroke = 4  e) Subacute stroke survivors  *SD for age not specified, only age range | Rehabilitation center/hospital  Physical therapists | a) Rehabilitation center  b) Physical therapists  c) Received VR gait training (VRT) on the Gait Real-time Analysis  Interactive Lab (GRAIL), which is a dual-belt treadmill combined with a motion-capture system and a screen (180° semi cylindrical).  It consisted of different VR environments (to train reactive balance, maneuverability, or dual tasks with modifiable difficulty levels) with specific rehabilitation goals and real-time feedback.  d) Dose:  1) Amount = 30 min/session  2) Frequency = twice/week  3) Duration = 6 weeks | a) Rehabilitation center  b) Physical therapists  c) Received conventional treadmill training (10–15 minutes) and functional gait exercises which included 6 directional exercises (15 minutes). The intervention was progressive.  c) Dose:  1) Amount = 30 min  2) Frequency = twice/week  3) Duration = 6 weeks | Baseline, 6 weeks, 3 months post-intervention  Outcomes:  1) USER-P  2) SIS-16  3) Fatigue Severity Scale 4) HADS  5) Falls Efficacy Scale International  6) SSQOL  7) Timed “Up & Go” Test  8) 6-minute walking test  9) Mini Balance Evaluation Systems Test  10) Daily-life walking activity measured with a triaxial accelerometer | a) Both groups showed significant improvements in participation and  dynamic balance over time as quantified by the USER-P  restrictions subscale (monthly increase of 2.53 points; 95% CI = 1.52 to 3.54, *p* < .001), the USER-P frequency subscale (1.05, 95% CI = 0.50 to 1.61, *p* < .001), and the Mini-BESTest score (0.62, 95% CI = 0.33 to 0.91, *p* < .001)  No other significant outcomes  b) 9.1% |
| Faria et al. (2016) Portugal | Single-blind RCT | a) N = 18  IG = 9, CG = 9  b) IG: median (IQR) age = 58 (48–71)  CG: median (IQR) = 53 (50.5–65.5)  c) IG: Female = 5  CG: Female = 5  d) Type of stroke not specified  e) The phase of stroke: median (IQR) of 7 (4–49 for IG and 4 (3–11.5) for CG. | Hospitals /  Psychologist or  occupational therapist | a) Hospital  b) Psychologist  c) In addition to conventional motor rehabilitation, participants received cognitive rehabilitation through simulation of ADLs with the VR based system Reh@City (a desktop computer, a 24” LCD monitor, and an arcade type of joystick).  It consisted of interactive cognitive training (3-dimensional environment) to accomplish common ADLs in a supermarket, a post office, a bank and a pharmacy.  Participants were given goal instructions in a task.  It also allowed increase/decrease of the visuo-spatial orientation.  There were visual feedback elements (to provide feedback on the accomplishment) and attention training tasks.  d) Dose:  1) Amount = 20 min/session  2) Frequency = 12 sessions over 4 – 6 weeks | a) Hospital  b) Occupational therapist  c) In addition to conventional motor rehabilitation, participants received time-matched cognitive rehabilitation through traditional methods (e.g., puzzles, calculus, problem resolution, and shape sorting)  c) Dose:  1) Amount = 20 min/session  2) Frequency = 12 sessions over 4 – 6 weeks | Baseline, post-intervention  Outcomes:  1) ACE  2) Trail Making Test A and B (TMT A  and B)  3) WAIS III  4) SIS 3.0  5) SUS | a) *Global cognitive functioning*  - IG improved significantly more than CG in general cognitive functioning (assessed by ACE) (U=13.500, Z=−2.388, *p*=.014, r =.56) and MMSE (U=18.000, Z=−1.996, *p* =.050, r =.47).  - IG demonstrated significantly higher scores in the attention domain (U= 17.000, Z=−2.066, *p* =.040, r=.49).  - There were significant differences between groups in the fluency task (U=13.000, Z=−2.487, *p* =.014, r=.59).  *Attention*  - No significant differences between group for the number of errors in TMT A (U= 40.000, Z=.047, *p*=1, r=.01) and TMT B (U=35.500, Z=−.482, *p* =.666, r=.11).  Executive functions  - No significant difference between groups in Picture Arrangement test scores (WAIS III).  *QoL*  - No significant differences between groups under SIS.  *Usability*  - Good levels of usability and satisfaction (Mdn=80/100, IQR=75–87.5).  b) 0% |
| Huber et al  (2025)  Switzerland | Single-blind, parallel, RCT | a) N = 46  IG = 24, CG = 22  b) IG: mean age = 68.75 ± 8.51  CG: mean age = 63.18 ± 9.69  c) IG: Female = 5  CG: Female = 4  d) IG: ischemic stroke = 19, hemorrhagic stroke = 4  CG: ischemic stroke = 17, hemorrhagic stroke = 5  e) Chronic stroke survivors | Hospitals/ rehabilitation centers /  Trained movement scientist/therapist | a) Hospitals, rehabilitation centers  b) Trained movement scientist  c) In addition to usual care, participants received concept-guided, personalized, motor-cognitive exergame (Dividat Senso which included pressure-sensitive plate, handrails in use, and a screen showing targets).  Intervention provided personalized progression and variability in training considering principles for neuroplasticity, motor learning, and training. It also offered real-time feedback on the participant’s performance through visual, auditory, and tactile cues, which helped the participant engaged more effectively with the video games.  d) Dose:  1) Amount = 30 – 40 min  2) Frequency = twice/week  3) Duration = 12 weeks  e) Participants received one-to-one training sessions, supervised by trained movement scientists of the study team. | a) Hospitals, rehabilitation centers  b) Not specified  In addition to the usual care (physical and cognitive activities), participants also received a weekly phone call (5- to 10-min conversations) to balance contact to the study team.  c) Dose:  1) Amount = 5-10 min  2) Frequency = weekly  3) Duration = 12 weeks | Baseline, 12 weeks, 24 weeks  Outcomes:  1) MoCA  2) German SIS 3.0  3) Vienna Test System (VTS); SRT, TMT, NBT, MRT)  4) 10MWT  3) OWA | a) The SIS domain Mobility showed a significant interaction effect for IG (T2, ITT: *p* = .03, r = .24; PP: *p* = .06, r = .23).  For cognitive assessment, a significant interaction effect in favor of the IG (intrinsic visual alertness) (T2, ITT: *p* = .02, r = .26; PP: *p* = .04, r = .25).  Per protocol analyses showed significant interaction effects in favor of IG for mistakes in TMT-B (T1, *p* = .01; T2, *p* = .02), and NBT (T2, *p* = .02).  No significant interaction effects for any parameter related to 10MWT.  Outdoor gait speed (T1, ITT: *p* = .02, r = .25; PP: *p* = .11, r = 0.20) in favor of IG.  Swing width unaffected measured outdoors (T1, ITT: *p* = .004, r = .31; PP: p = 0.02, r = 0.29 / T2, ITT: *p* = .003, r = .33; PP: *p* = .007, r = .33) in favor of IG.  No other significant outcome findings.  *Safety*  - A participant with a pre-existing heart condition experienced an adverse event that was mild and potentially associated with the intervention in terms of timing, though not necessarily caused by it.  b) 19.6% |
| Kilinc et al. (2023)  Turkey | Assessor-blinded RCT | a) N = 30  IG = 15, CG = 15  b) IG: median age = 61 (46-65)  CG: median age = 52.5 (42-65)  c) IG: Female = 4  CG: Female = 7  d) IG: ischemic stroke = 11, hemorrhagic stroke = 4 CG: ischemic stroke = 12, hemorrhagic stroke = 2  e) Chronic stroke survivors | Hospital/  Physiotherapists | a) Department of Physical Medicine and Rehabilitation  b) Physiotherapists  c) A combination of virtual balance training (VBT) and conservative rehabilitation.  VBT used Thera-Trainer Balo (TTB) device which comprises 2 cylinders connected to a base plate with knee support bars and pelvis support table. Sensors were used and an avatar was displayed on screen to determine the center of gravity.  Activities included collecting objects arranged in a circle and dropping them into the storage unit of their center of gravity. Participants were trained at 50% range of motion and the difficulty level would be increased according to the precision and time pressure of the avatar when collecting objects.  d) Dose: 1) Amount = 20 min/session (VBT) + 60 min conservative rehabilitation  2) Frequency = 4 times/week 3) Duration = 8 weeks | a) Department of Physical Medicine and Rehabilitation  b) Physiotherapists  c) Patient-specific conservative program which included exercises to 1) strengthen affected side, 2) increase range of motion, 3) strengthen muscles, 4) enhance balance and coordination.  d) Dose: 1) Amount = 60 mins/session  2) Frequency = 4 times/week 3) Duration = 8 weeks | Baseline, post-intervention  Primary outcomes:  1) BBS  Secondary outcomes:  1) BI  2) NIHSS  3) BMR  4) FAS  5) SF-36 | a) There were no significant differences observed between groups in BBS scores, BMR stages, FAS scores, SF-36 scale scores, and spasticity degrees at post-intervention.  b) 3.3% |
| Kim (2018) South Korea | Single-blind RCT | a) N = 24  IG = 12, CG = 12  b) IG: mean age = 50.91 ± 9.57  CG: mean age = 57.23 ± 14.63 years  c) IG and CG: Male = 15, Female = 9  d) No information  e) Chronic stroke survivors | Not specified / occupational therapist | a) Not specified b) Occupational therapist c) Virtual reality exercise program using video games (Wii Sports and Wii Fit) + traditional rehabilitation d) Dose: 1) Amount = 40 min/session 2) Frequency = 3 times/week 3) Duration = 12 weeks | a) Not specified b) Occupational therapist c) Traditional rehabilitation (exercise program): d) Dose: 1) Amount = 30 min/session 2) Frequency = 5 times/week 3) Duration = 12 weeks | Baseline, 12 weeks (post-intervention) Outcomes: 1) Upper extremity function - FMA and MFT 2) SIS | a) A statistically significant difference in SIS scores was found between the two groups (*p* <.005). No statistically significant differences were found in FMA and MFT scores between groups. b) 0% |
| Kuo et al (2023) Taiwan | Single-blind RCT | a) N = 37  IG = 19, CG = 18  b) IG: mean age = 57.47 ± 6.99  CG: mean age = 59.5 ± 10.65  c) IG: Female = 6  CG: Female = 3  d) IG: ischemic stroke = 10, hemorrhagic stroke = 9 CG: ischemic stroke = 4, hemorrhagic stroke = 14  e) Chronic stroke survivors | Not specified / occupational therapist | a) Not specified b) Occupational therapist c) VR game (a television, PABLO Tyromotion device, and immersive VR games) with two motion sensors and a grip sensor attached to the participant. No head-mounted device used. d) Dose: 1) Amount = 30 min/session (intervention) + 30 min of conventional occupational therapy 2) Frequency = 2 times/week 3) Duration = 9 weeks | a) Not specified b) Occupational therapist c) Standard rehabilitation -conventional occupational therapy program d) Dose: 1) Amount = 60 min/session 2) Frequency = 2 times/week 3) Duration = 9 weeks | Baseline, 9 weeks (immediate post-intervention)  Primary outcome: 1) FMA-UE Secondary outcomes: 1) active ranges of motion (AROMs) of the shoulder and elbow 2) hand grip strength - Jamar dynamometer 3) unilateral gross manual dexterity - BBT 4) SIS (strength, hand function and ADL/IADL) | a) IG exhibited greater improvements in the hand dexterity (*p* = .05), shoulder flexion (*p* = .03) and elbow pronation between groups (*p* = .03).  b) 8.1% |
| Laffont et al (2020) France | Multicentric single-blind RCT | a) N = 51  IG = 25, CG = 26  b) IG: mean age = 60.8 ± 14.1  CG: mean age = 55.8 ± 14.0  c) IG: Female = 9  CG: F = 11  d) IG: ischemic stroke = 22, hemorrhagic stroke = 3 CG: ischemic stroke = 16, hemorrhagic stroke = 10  e) Subacute stroke survivors | Physical and rehabilitation medicine departments / occupational therapist | a) Hospital b) Occupational therapist c) VR + standard care [physiotherapy (30 min) and occupational therapy sessions (30-60min) per day depending on patient's needs] d) Dose: 1) Amount = 15-45 min (virtual reality), 60-90 min (standard care) 2) Frequency = 5 times/week 3) Duration = 6 weeks | a) Hospital b) Occupational therapist c) Standard care [physiotherapy and occupational therapy sessions (a total of 60-90 min) per day depending on patient's needs] + additional sessions of occupational therapy d) Dose:  1) Amount = 15-45 min (additional occupational therapy), 60-90 min (standard care) 2) Frequency = 5 times/week 3) Duration = 6 weeks | Baseline, 6 weeks, 6 months  Primary outcome: 1) UL-FMS Secondary outcomes: 1) BBT 2) Motor function of upper limbs - WMFT 3) MAL 4) SF-36 5) BI | a) At 6 weeks, gain in UL-FMS did not significantly differ between the groups (*p* = .10), but BBT was significantly improved the IG (*p* = .02). b) 9.8% |
| Lam et al (2022) Hong Kong  Lam et al. (2020)  *Another report | Single-blind RCT | a) N = 93  IG = 47, CG = 46  b) IG: mean age = 65.1 ± 10.2 years  CG: mean age = 66.0 ± 9.0  c) IG: Female = 20  CG: Female = 18  d) IG: ischemic stroke = 38, hemorrhagic stroke = 9 CG: ischemic stroke = 38, hemorrhagic stroke = 8  e) Subacute stroke survivors | Stroke rehabilitation in hospital / physiotherapists | a) Geriatric day hospital b) not specified for the intervention of computer games training c) Bilateral movement computer games training (3 different computer games) + conventional therapy (1.5 h of conventional physiotherapy and 1.5 h of multidisciplinary occupational therapy) d) Dose: 1) Amount = 30 min (computer games training) + 3 h conventional therapy 2) Frequency = 2 times/week 3) Duration = 8 weeks | a) Geriatric day hospital b) Monitored by a patient care assistant c) Video-directed conventional training (30 min) + conventional therapy (1.5 h of conventional physiotherapy and 1.5 h of multidisciplinary occupational therapy) d) Dose: 1) Amount = 30 min (video-directed conventional training) + 3 h conventional therapy 2) Frequency = 2 times/week 3) Duration = 8 weeks | Baseline, 4 weeks, 8 weeks (post-intervention), 12 weeks (4 weeks post-intervention)  Primary outcome: 1) FMA-UE Secondary outcomes: 1) ARAT 2) Grip Strength - digital dynamometer 3) SF-36 | a) Participants in IG demonstrated greater improvements in FMA-UE scores from mid-intervention to 1 month follow-up than CG. IG showed better improvements in ARAT scores than CG from post-intervention to 1 month follow. No significant differences were found in grip strength and SF-36 scores between groups.  b) 10.8% |
| Latif et al. (2025)  Indonesia | Single-centered, RCT | a) N = 60  IG = 30, CG = 30  b) IG: mean age = 58.43 ± 8.89 years  CG: mean age = 53.97 ± 11.09  c) IG: Female = 9  CG: Female = 14  d) Type of stroke not mentioned  e) A mixture of stroke survivors | Hospital outpatient care/therapists | a) Outpatient clinic b) Physiotherapists c) Received a combination of 1) VR therapy using MusicGlove Hand Therapy tools and 2) joint range of motion and fine motor exercises (conventional therapy) d) Dose (*for VR therapy and conventional therapy)  1) Amount = 1 hour and 15 minutes/session 2) Frequency = not reported 3) Duration = 8 weeks | ) Outpatient clinic b) Occupational therapists c) Received joint range of motion and fine motor exercises  d) Dose:  1) Amount = 1 hour/session  2) Frequency = not reported 3) Duration = 8 weeks | Baseline, 4 weeks, 8 weeks (post-intervention), 9 weeks (1 post-intervention)  Outcomes: 1) Hand function - FMA-UE 2) Fine motor skills - 9-HPT | a) At week 4, both groups had no significant difference in hand functional status (*p* = .284). There were significant differences in hand functional status between groups at week 8 (*p* = .045) and 1 week after intervention (*p* = .037).  There were significant changes in fine motor function between groups at all time points (*p* < .05), except between immediate post intervention and one week post intervention.  b) 0% |
| Lee et al. (2016) South Korea | Single-blind RCT | a) N = 20  IG = 10, CG = 10  b) IG = 69.20±5.514  CG = 73.13±8.983  c) IG: Female = 5  CG: Female = 3  d) IG: ischemic stroke = 7, hemorrhagic stroke = 3.  CG: ischemic stroke = 4, hemorrhagic stroke = 4.  e) Chronic stroke survivors  *These data do not include the two participants who dropped out | Hospital /  Not specified | a) Hospital  b) Not specified  c) Participants received VR-based bilateral  upper extremity training (VRBT) which consisted of visual feedback.  The animation comprised: symmetric UE training (0° and 45°) and asymmetric UE training (0° and 45°).  Equipment: a laptop, webcam, and a 23-inch monitor.  d) Dose:  1) Amount = 30 min/session  2) Frequency = 3 days weekly  3) Duration = 6 weeks  e) Also received conventional occupational therapy for 30 min/session, 5 days/week for 6 weeks | a) Hospital  b) Not specified  b) Bilateral  upper extremity training (BT) – same UE training as VRBT group  b) Dose:  1) Amount = 30 min/session  2) Frequency = 3 days weekly  3) Duration = 6 weeks  e) BT group watched an irrelevant video.  Also received conventional occupational therapy for 30 min/session, 5 days/week for 6 weeks | Baseline, post-intervention  Outcomes:  1) JHFT  2) BBT  3) GPT  4) DMMT  5) Jamar Plus Hands on Evaluation Kit | a) IG demonstrated significant improvements in JHFT (five items, *p* <.05), BBT (*p* =.029), and GPT (*p* =.009) when compared with CG.  Also, IG was statistically significantly improved in DMMT (elbow flexion/extension) and hand strength test (all *p*-values < .01) when compared to CG.  b) 10% |
| Lee et al (2017) Taiwan | Single-centered, RCT | a) N = 50  IG = 26, CG = 24  b) IG: mean age = 59.35 ± 8.95  CG: mean age = 55.76 ± 9.59  c) IG: Female = 10  CG: Female = 3  d) IG: ischemic stroke = 16, hemorrhagic stroke = 10 CG: ischemic stroke = 14, hemorrhagic stroke = 7  e) Chronic stroke survivors | Hospital / occupational therapist | a) Neurorehabilitation Unit b) Occupational therapist c) VR (comprised a television, Microsoft Kinect, and a commercial game) + standard treatment  d) Dose: 1) Amount = 45 min interactive virtual reality balance-related games, 45 min standard treatment 2) Frequency = Twice/week 3) Duration = 6 weeks | a) Neurorehabilitation Unit b) Occupational therapist c) Standard treatment - strengthening, endurance training, ambulation, and ADL training d) Dose: 1) Amount = 90 min 2) Frequency = Twice/week 3) Duration = 6 weeks | Baseline, 6 weeks (post-intervention), 12 weeks (follow-up)  Primary outcome: 1) BBS Secondary outcomes: 1) FRT16 2) TUG cognition test 3) MBI 4) ABC 5) SIS  For both groups, modified Physical Activity Enjoyment Scale (M-PAES) and incidence for adverse events were recorded for each session. | a) Both groups demonstrated significant improvement over time in the BBS (*p* =.000) and TUG-cog test (*p* =.005).  IG reported to have higher M-PAES scores compared to CG (*p* =.027). No other significant differences were found.  b) 6% |
| Long et al. (2020) China | Assessor-blinded RCT | a) N = 60  IG = 30, CG = 30  b) IG = 53.28±15.30  CG = 54.11±14.81  c) IG: Female = 7  CG: Female = 11  d) IG: ischemic stroke = 21, hemorrhagic stroke = 4.  CG: ischemic stroke = 19, hemorrhagic stroke = 8.  e) Acute/ subacute  /chronic stroke survivors (onset time ≤ 1 year) | Acute hospital / therapists | a) Acute hospital  b) Therapist (not specified)  c) Used VR-based game system (Doctor Kinetic) with a touch-controlled computer screen, infrared sensor smart recognition camera, and a human-shaped model on the computer screen.  VR games consisted of five tasks including bilateral upper limb flexion; abduction activity; gold coins picking game, including shoulder circle; cross and mixed training for 3–5 min.  Difficulty and intensity were adjusted according to the participant’s ability.  d) Dose:  1) Amount = 45 min/session  2) Frequency = 5 times weekly  3) Duration = 3 weeks  e) Received conventional training as well | a) Acute hospital  b) Therapist (not specified)  c) Received conventional training  d) Dose:  1) Amount = 45 min/session  2) Frequency = 5 times weekly  3) Duration = 3 weeks | Baseline, post-intervention (after 3-week intervention)  Outcomes:  1) COPM  2) SSEQ  3) FMA-UE  4) FTHUE  5) MBI | a) A significant difference between groups in SSEQ was observed after intervention (Median Difference = 8, *p* = .043, Z = − 2.027).  And only daily activities  domain in SSEQ demonstrated significance (Median Difference = 6, *p* = .017, Z = − 2.392).  No significant differences in COPM were found between groups.  A significant difference in MBI was found between  groups (Median Difference = 10, *p* = .03, Z = − 2.171).  No significant between-group difference in FMA-UE and FTHUE was noted, and both groups had improved upper limb function (*p* < .05).  b) 13.3% |
| Marques-Sule et al. (2021) Spain | Single-blind RCT | a) N = 29  IG = 15, CG = 14  b) IG: mean age = 61.5 ± 8.4  CG: mean age = 58.2 ± 7.4,  c) IG: Female = 6  CG: Female = 5  d) Type of stroke not specified  e) Chronic stroke survivors | Lab of university rehabilitation clinic / Physical therapist | a) Lab of university rehabilitation clinic  b) Physical therapist c) Virtual rehabilitation program using Nintendo Wii with the Wii Remote and Wii Balance Board in addition to conventional PT.  d) Dose:  1) Amount = 30 min/session  2) Frequency = twice/week  3) Duration = 4 weeks  e) Lower limb balance training (15 minutes) and upper limb  training (15 minutes) | a) Lab of university rehabilitation clinic  b) Physical therapist  c) Received conventional PT  c) Dose:  1) Amount = 30 min/session  2) Frequency = twice/week  3) Duration = 4 weeks | Pre-intervention, post-intervention  Outcomes:  1) TUG  2) POMA  3) BBS  4) FMA-UL  5) BI  6) FAI | a) Significant differences in time for TUG (*p*< .001), POMA-total (*p* = .009), BBS (P = .003), FMA-UL (*p* = .0002), BI (*p* = .016), FAI (*p* < .001).  Significant differences for group*time interaction for TUG (*p* = .018), POMA-total (*p* = .001), BBS (*p* = .002), BI (*p* = .016), FAI (*p* < .001)  b) 0% |
| Mazher et al. (2025)  Pakistan | Double blinded RCT | a) N = 32  IG = 16, CG = 16  b) IG: mean age = 52.94 ± 5.28  CG: mean age = 56.17 ± 6.44  c) IG: Female = 3  CG: Female = 6  d) IG: ischemic stroke = 11, hemorrhagic stroke = 7 CG: ischemic stroke = 14, hemorrhagic stroke = 2  e) Subacute stroke survivors | Hospital/  Not specified | a) Physical therapy department b) Not specified  c) Xbox kinetic-based rehabilitation training for upper limbs (e.g., programs such as Boxing and Bowling in Kinect Sports Pack, Rally Ball, 20,000 leaks and Space Pop in the Kinect Adventure Pack) plus standardized physical therapy treatment d) Dose: 1) Amount = 30 min/session 2) Frequency = 5 times/week 3) Duration = 4 weeks | a) Physical therapy department b) Not specified  c) Conventional exercise therapy in addition to training in daily tasks such as feeding, grooming, dressing, using the restroom, and transferring  d) Dose: 1) Amount = 30 min/session 2) Frequency = 5 times/week 3) Duration = 4 weeks | Baseline, 4 weeks (post intervention), 8 weeks  Outcomes:  1) FMA-UE  2) SIS Version 3.0 | a) There were statistically significant differences between groups at post intervention and follow-up in QoL (*p* < .05). As for the upper extremity function, a statistically significant difference was observed between groups at 8 weeks.  b) 0% |
| Oh et al. (2019) South Korea | Single-blinded RCT | a) N = 33  IG = 18, CG = 15  b) IG = 57.4±12.2  CG = 52.6±10.7  c) IG: Female = 5  CG: Female = 5  d) Type of stroke not specified  e) Chronic stroke survivors | Not reported / Occupational therapist | a) Not specified  b) Occupational therapist  c) VR combined real instrument training.  Joystim, a 3-dimensional manipulator consists of a monitor, conventional computer, and real instruments (e.g., thumb pinch, doorknob, button, air tube, gas valve, tool turn, steering wheel) with 3° of freedom.  It consisted of nine modules, basic tools, games each, and two missions.  The level of difficulty could be adjusted based on each participant’s performance during the training period.  d) Dose:  1) Amount = 30 min/session  2) Frequency = 3 days weekly  3) Duration = 6 weeks | a) Not specified  b) Occupational therapist  c) Conventional occupational therapy which included UE training with task-related exercises and ADL board, hand fine motor training with pegboards, and perception and cognition training.  The level of difficulty could be adjusted based on each participant’s performance during the training period.  d) Dose:  1) Amount = 30 min/session  2) Frequency = 3 days weekly  3) Duration = 6 weeks | Pre-intervention. Post-intervention, 4 weeks post-intervention  Outcomes:  1) FMA-UE  2) MMT  3) Hand grip test  4) BBT  5) 9-HPT  6) MAS  7) K-MMSE  8) K-MoCA | a) IG demonstrated significant improvements in the MMT (*p* =.039) and MAS wrist extension (*p* =.041), MAS for elbow flexion (*p* =.022), BBT (*p* =.002) in a time dependent manner.  IG showed significant improvements in BBT (*p* =.010), 9-HPT (*p* =.025), lateral (*p* =.005), palmar (*p* =.012), and tip pinch (*p* =.006). Lateral pinch power was maintained at the follow-up (*p* =.002).  Both groups significantly improved in MMT finger extension, FMA-UE, grip power, palmar pinch power, tip pinch power, 9-HPT, K-MMSE, and K-MoCA.  Improvement rate in the tip pinch power (*p* =.036) scores and MAS for elbow flexion (*p* =.041) between the pretraining and post training periods was significantly higher in IG.  b) 6.1% |
| Ozen et al (2021) Turkey | Single-centered, RCT | a) N = 38  IG = 20, CG = 18  b) IG: mean age = 62.00 ± 13.12  CG: mean age = 69.8 ± 8.41  c) IG: Female = 5  GG: Female = 5  d) IG: ischemic stroke = 12, hemorrhagic stroke = 3 CG: ischemic stroke = 13, hemorrhagic stroke = 2  e) Subacute - chronic stroke patients (≥ three months since stroke) | University hospital / physiotherapist and occupational therapist | a) Physical Medicine and Rehabilitation ward b) Physiotherapist and occupational therapist c) Computer game assisted task specific exercises (CGATSE) + conventional therapy: 30 min CGATSE using the Rehabilitation Joystick for Computerized Exercise (Rejoyce) system d) Dose: 1) Amount = 30 min of CGATSE and 1 h physical therapy 2) Frequency = 5 times/week 3) Duration = 4 weeks | a) Physical Medicine and Rehabilitation ward b) Physiotherapist and occupational therapist c) Conventional neurorehabilitation physical therapy + occupational therapy: 1 h of proprioceptive neuromuscular and neurodevelopmental facilitation techniques, range of motion, strengthening exercises, balance-coordination, and ambulation training 30 min occupational therapy - task-based exercises d) Dose: 1) Amount = 30 min of occupational therapy, 1 h physical therapy 2) Frequency = 5 times/week 3) Duration = 4 weeks | Baseline, 4 weeks  Primary outcomes: 1) FMA-UE 2) BSSR Secondary outcomes: 1) MoCA 2) SSQOL | a) While FMA-UE, BSSR arm, and SSQOL improved significantly in both groups (*p* < 0.05), no significant between-group differences were observed.  b) 21.1% |
| Park & Ha (2023)  South Korea | 3-arm RCT  (One of the control groups (CG1) is excluded in this review as it included computer-assisted cognitive rehabilitation with some game elements) | a) N = 41  IG = 21, CG2 = 20  b) IG: mean age = 62.5 ± 4.7  CG2: mean age = 62.5 ± 4.8 years  c) IG: Female = 9  CG: Female = 8  d) IG: ischemic stroke = 11, hemorrhagic stroke = 9  CG: ischemic stroke = 12, hemorrhagic stroke = 8  e) Subacute - chronic stroke patients | Hospital/  Occupational therapist | a) Virtual reality room b) An occupational therapist c) One-on-one patient-specific individualized program with 30 min of conventional cognitive rehabilitation (morning) and 30 min of VR-based cognitive rehabilitation (afternoon).  VR-based cognitive (grounded in self-efficacy theory) consisted of immersive programs (e.g., Finding same fishes’, ‘Save the planet’, ‘Throwing dar’, and ‘Other contents’) with goggles and controllers (15 min of VR and 15 of individual training using a stroke cognitive recovery workbook).  Group discussions (4-5 participants in each group) were conducted every Friday.  d) Dose: 1) Amount = 30 min (conventional) + 30 min (VR-based) 2) Frequency = 5 times/week 3) Duration = 8 weeks | a) Rehabilitation therapy room  b) An occupational therapist c) Conventional cognitive rehabilitation (one-on-one) included paper-and-pencil tasks such as puzzles, calculation, picture matching, and others.  d) Dose: 1) Amount = 30 min morning and afternoon 2) Frequency = 5 times/week 3) Duration = 8 weeks | Baseline, 4 weeks, 8 weeks  Outcomes:  1) Stroke self-efficacy  2) Cognitive function – K-MMSE – 2:SV  3) Visual perception – MVPT-3  4) Activities of daily living – K-MBI  5) Health-related QoL – SF-12 | a) There were statistically significant differences between groups and time points in stroke self-efficacy (F = 78.62, *p* < .001), cognitive function (F = 9.33, *p* < .001), ADLs (F = 14.15, *p* < .001), and HRQoL (F = 213.87, *p* < .001).  b) 2.4% |
| Park et al. (2021)  South Korea | Single-site RCT | a) N = 44  IG = 22, CG = 22  b) IG: mean age = 60.59 ± 18.12,  CG: mean age = 62.29 ± 13.97 years  c) IG: Female = 10  CG: Female = 10  d) Type of stroke not specified  e) Acute stroke patients | Not reported/  Therapists | a) Not reported b) Therapists c) Game-based upper limb training with RAPAEL Smart GloveTM + conventional physical therapy.  Functional training and ADLs such as catching butterflies and balls, squeezing an orange are adjusted based on the level of difficulty suitable for participants.  d) Dose (intervention): 1) Amount = 30 min + 30 min (conventional physical therapy)  2) Frequency = 5 times/week 3) Duration = 4 weeks | a) Not reported b) Therapists c) Conventional physical therapy based on the exercise and tools to improve upper limb function (passive/active should joint and hand functions) and ADLs (basic, instrumental activities).  d) Dose: 1) Amount = 30 min  2) Frequency = 5 times/week 3) Duration = 4 weeks | Baseline, post-intervention (end of 4-week)  Outcomes:  1) Upper limb function  - FMA-UE  - Hand strength  - JTHFT  2) ADLs  - K-MBI | There were no significant interactions between time and group in FMA.  There were significant differences in hand strength, JTHFT, and K-MBI between two groups over time.  b) 0% |
| Rémy-Néris et al (2021) France | Multi-centered, RCT | a) N = 215  IG = 107, CG = 108  b) IG: mean age = 58.08 ± 14.05  CG: mean age = 58.53 ± 13.27  c) IG: Female = 40  CG: Female = 35  d) IG: ischemic stroke = 77, hemorrhagic stroke = 30 CG: ischemic stroke = 75, hemorrhagic stroke = 33  e) Subacute stroke survivors | Hospital / physiotherapist and occupational therapist | a) Rehabilitation centers b) Physiotherapists and occupational therapists c) Gravity-supported, games-based training using an exoskeleton + usual rehabilitation: The ArmeoSpring exoskeleton device was used to train movements (shoulder and elbow), pronation and supination, and grip-release. d) Dose: 1) Amount = 30 min x 2 times game-based training, 1.5 h upper limb rehabilitation 2) Frequency = 5 days/week 3) Duration = 4 weeks | a) Rehabilitation centers b) Physiotherapists and occupational therapists c) Basic stretching and active exercises + usual rehabilitation d) Dose: 1) Amount = 30 min x 2 times basic stretching and active exercises, 1.5 h upper limb 2) Frequency = 5 days/week 3) Duration = 4 weeks | Baseline, 30 days, 3 months, 6 months, 12 months  Primary outcome: 1) FMA-UE Secondary outcomes: 1) Change in sensorimotor impairment (FMA-UE) 2) Change in severity of shoulder pain - VAS 3) Change in spasticity - MAS 4) Function in self-care, continence, mobility, transfer, communication, and cognition - FIM 5) ARAT 6) EQ-5D 7) SIS 8) Cost utility 9) Participants' perception of exercise intervention | a) No significant between-group differences were observed in any outcome measures at any time point.  b) 20% |
| Rodríguez-Hernández et al. (2021)  Spain | 2-arm, single-site RCT | a) N = 46  IG = 23, CG = 23  b) IG: mean age = 62.6 ± 13.5  CG: mean age = 63.6 ± 12.2  c) IG: Female = 5  CG: Female = 3  d) IG: ischemic stroke = 21, hemorrhagic stroke = 2 CG: ischemic stroke = 18, hemorrhagic stroke = 2  e) Subacute stroke survivors | General hospital/  Physiotherapist and occupational therapist | a) Hospital rehabilitation unit b) Physiotherapist and occupational therapist c) VR plus conventional therapy.  Motor training with virtual reality devices: Hand Tutor© glove and 3DTutor© which were based on intensive and repetitive practice through movement and feedback instructions provided by the software with virtual environments and tasks that simulate movements that stroke survivors require for daily life. The Rehametrics© software [32] + Microsoft Kinect sensor was used for the recovery of the upper limb, trunk, and lower body.  By monitoring and capturing real-time movement, the system allowed the therapist to adjust treatment parameters, including difficulty, duration, range of motion, and the number of distracting/visual aids.  d) Dose: 1) Amount = VR (50 min) + conventional therapy (100 min)  2) Frequency = 5 consecutive days/week 3) Duration = 3 weeks | a) Hospital rehabilitation unit  b) Physiotherapist and occupational therapist c) Conventional therapy consisted of manual therapy techniques (massage); passive and active assisted mobilization; walking in parallel; exercises with and without resistance; active-assisted mobility exercises (upper limb and fingers) in a sitting position; moving objects horizontally on a table; elevation and superposition of objects; biomechanical tasks. d) Dose: 1) Amount = 75 min of physiotherapy + 75 min of occupational therapy 2) Frequency = 5 consecutive days/week 3) Duration = 3 weeks | Baseline, 3 weeks after the start (post-intervention), and 3 months after its completion (follow-up)  Outcomes: 1) EQ-5D-5L  2) EQ – VAS | a) IG had statistically significant higher EQ-VAS scores at post-intervention and follow-up when compared to CG.  At post-intervention and follow-up, IG demonstrated significantly better HRQoL compared to CG across most EQ-5D-5L dimensions (except for pain/ discomfort).  b) 6.5% |
| Shin et al (2015) South Korea | Single-blind, RCT | a) N = 35  IG = 18, CG = 17  b) IG: mean age = 53.3 ± 11.8  CG: mean age = 54.6 ± 13.4  c) IG: Female = 5 CG: Female = 3  d) No information  e) Chronic stroke survivors | Not specified / occupational therapists | a) Not specified b) Occupational therapists c) VR + occupational therapy: Game-based VR rehabilitation with the RehabMaster™ system [compliant depth sensor, 3D awareness sensor, infrared projectors (60-inch monitor), and image sensors for VR]. Participants sat on a chair in front of the monitor and depth sensor and moved their upper limbs/trunk based on the training protocol. d) Dose: 1) Amount = 30 min virtual reality rehabilitation, 30 min conventional occupational therapy 2) Frequency = 5 days/week 3) Duration = 4 weeks | a) Not specified b) Occupational therapists c) Occupational therapy: Included range of motion and strengthening exercises, table-top activities, and activities of daily living training d) Dose: 1) Amount = 30 min conventional occupational therapy + 30 min additional occupational therapy  2) Frequency = 5 days/week 3) Duration = 4 weeks | Baseline, 4 weeks  Outcomes: 1) SF-36 2) HAMD 3) FMA-UE | a) A significant difference between groups was observed for role limitation due to physical problems (*p* =.031). No other significant differences were found.  b) 8.6% |
| Shin et al (2016) South Korea | Single-blind, RCT | a) N = 46  IG = 24, CG = 22  b) IG: mean age = 57.2 ± 10.3  CG: mean age = 59.8 ± 13.0  c) IG: Female = 5  CG: Female = 5  d) IG: ischemic stroke = 15, hemorrhagic stroke = 9 CG: ischemic stroke = 14, hemorrhagic stroke = 8  e) There is no specified information found on the phase of stroke | National Rehabilitation Center / occupational therapist | a) Rehabilitation hospital b) Occupational therapists c) The RAPAEL Smart Glove: Glove-shaped sensor device and a software application (training games in the system with intended upper extremity movements).  d) Dose: 1) Amount = 30 min Smart glove + 30 min standard occupational therapy 2) Frequency = 5 days/week 3) Duration = 4 weeks | a) Rehabilitation hospital b) Occupational therapists c) Standard occupational therapy d) Dose: 1) Amount = 1 h  2) Frequency = 5 days/week 3) Duration = 4 weeks | Baseline, middle of the intervention (after the 10th session), immediately after the intervention, and 1 month after the intervention.  Primary outcome: 1) FMA-UE Secondary outcomes: 1) Hand function - JTT and PPT 2) SIS v3.0 | a) The FM (FM total, *p* =.006; FM-prox, *p* =.007; FM-dist, *p* =.024, JTT (JTT total, p=.032; JTT-gross, *p* =.025), and SIS (composite, *p* =.021; overall score, *p* =.015) scores were significantly greater in IG than CG.  No significant differences were found in PPT scores between two groups.  b) 50% |
| Şimşek & Çekok (2016) Turkey | Single-centered, RCT | a) N = 44  IG = 22, CG = 22  b) IG: mean age = 54.15 ± 20.29  CG: mean age = 61.5 ± 11.63  c) IG: Female = 5  CG: Female = 8  d) IG: ischemic stroke = 11, hemorrhagic stroke = 9 CG: ischemic stroke = 9, hemorrhagic stroke = 13  e) Subacute stroke survivors | Medicalpark ˙Izmir Hospital (Physical Therapy and Rehabilitation) / physiotherapists | a) Department of Physical Therapy and Rehabilitation b) Physiotherapist c) Nintendo Wii video game (5 games) for upper limbs and balance training: 3 sets of game each, with 5 min interval d) Dose: 1) Amount = 45-60 min/session 2) Frequency = 3 times/week 3) Duration = 10 weeks | a) Department of Physical Therapy and Rehabilitation b) Physiotherapist c) Bobath neurodevelopmental treatment (conventional therapy): d) Dose: 1) Amount = 45-60 min/session 2) Frequency = 3 times/week 3) Duration = 10 weeks | Baseline, post-treatment (after 10 weeks)  Outcomes: 1) FIM 2) NHP 3) VAS | a) No significant differences were found between groups with regard to FIM and NHP. IG reported higher satisfaction of treatment when compared with CG (*p* <.001). b) 4.5% |
| Song & Lee (2021)  South Korea | Multi-centered RCT | a) N = 10  IG = 5, CG = 5  b) IG: mean age = 64.20 ± 7.08  CG: mean age = 60.00 ± 10.88  c) IG: Female = 2  CG: Female = 2  d) IG: ischemic stroke = 3, hemorrhagic stroke = 2 CG: ischemic stroke = 4, hemorrhagic stroke = 1  e) Subacute/  chronic stroke survivors (at least 6 months) | Hospital/  Not reported | a) Hospital (not specified) b) Multidisciplinary rehabilitation teams  c) In addition to 60 minutes of conventional rehabilitation, participants also had a VR-based bilateral arm training intervention which consisted of 1) daily life training, 2) visual perception and cognitive component, 3) exercise evaluation component, and 4) 14 visual perception tasks.  DK2 Oculus Rift and Oculus Rift controller were used (immersive VR experience), participants were sitting while performing the training.  d) Dose (intervention): 1) Amount = 30 min 2) Frequency = 5 times/week 3) Duration = 4 weeks | a) Hospital (not specified)  b) Multidisciplinary rehabilitation teams  c) Normal bilateral arm training group (e.g., turning on lights, arranging a chest of drawers) in a real environment + 60 minutes conventional rehabilitation d) Dose: 1) Amount = 30 min  2) Frequency = 5 times/week 3) Duration = 4 weeks | Baseline, post-intervention (after 4 weeks)  Outcomes:  Upper extremity function:  - EMG  - MFT  - Sensory function test (two-point discrimination, proprioception, and stereognosis). | There was no statistically significant difference between groups in MFT (*p* = .07), EMG analysis and sensory function tests except for proprioceptive test that showed a significant difference between groups (*p* = .04)  b) 0% |
| Térémetz et al (2022) France | Single-centered, RCT | a) N = 43  IG = 21, CG = 22  b) IG: mean age = 55.8 (95% CI: 49.7 - 61.9)  CG: mean age = 56.2 (95% CI: 50.5 - 61.9)  c) IG: Female = 6  CG: Female = 10  d) No information  e) Chronic stroke survivors | Hospital / physiotherapist | a) University hospital b) Physiotherapist c) Wii therapy (3 games): Tennis, gold, and boxing 15 min of each sitting on a stool d) Dose: 1) Amount = 1 h 2) Frequency = 3 times/week 3) Duration = 4 weeks | a) University hospital b) Physiotherapist c) Conventional therapy: Focused on functional exercise - passive and active movements of impaired joints and functional, task-orientated reaching and grasping exercises d) Dose: 1) Amount = 1 h 2) Frequency = 3 times/week 3) Duration = 4 weeks | Baseline, the week after intervention  Primary outcome: 1) change in elbow extension and forward trunk motion  Secondary outcomes: 1) Pain - VAS 2) Perceived effort - 10-point Borg scale 3) FMA-UE 4) BBT 5) ARAT 6) MAL 7) SIS | a) No significant differences in mean change in elbow extension angle (*p* =.61) and forward trunk position (*p* =.65). No significant between-group differences in change in FMA-UE, ARAT, BBT, MAL scores, and SIS. b) 7% |
| Velmurugan et al. (2023)  India | Parallel group design, single-blind RCT | a) N = 40  IG = 20, CG = 20  b) IG: mean age = 52.45±6.24  CG: mean age = 53.65±5.87  c) IG: Female = 7  CG: Female = 6  d) IG: ischemic stroke = 16, hemorrhagic stroke = 4 CG: ischemic stroke = 14, hemorrhagic stroke = 6  e) Subacute and chronic stroke survivors | Department of physiotherapy in a college/  physiotherapists | a) Physiotherapy department  b) Physiotherapist  c) Five games from Nintendo Wii (N-Wii) software video games including Wii sports and Wii flit for upper limbs (e.g., tennis, punches out, light-rope tension, tilt table, and heading)  d) Dose: 1) Amount = each games x 3 sets with 5 minutes intervals between sets (a total of 45 minutes) 2) Frequency = 5 sessions/week 3) Duration = 6 weeks | a) Physiotherapy department  b) Physiotherapist  c) Active control: Motor relearning program (e.g., activities such as opening/closing bottle lids and drinking water in a glass).  d) Dose: 1) Amount = each task was repeated 10-15 minutes on affected side (a total of 45 min) 2) Frequency = 5 sessions/week 3) Duration = 6 weeks | Baseline, after 6 weeks of intervention  Outcome:  Upper limb motor function – FMA | a) The intervention group demonstrated significantly higher improvement in FMA- upper limb motor recovery when compared to the control group.  b) 0% |

Note: 9-HPT = 9-Hole Peg Test; 10MWT = 10-Meter Walk Test; ABC = Activities-specific Balance Confidence; ACE = Addenbrooke Cognitive Examination; ARAT = Action Research Arm Test; BBS = Berg balance scale; BBT = Box and Block Test = BBT; BI = Barthel Index; BMR = Brunnstrom motor recovery; CG = control group; COPM = Canadian Occupational Performance Measure; EBI = Extended Barthel Index; EMG = Electromyography; FAI = Frenchay Activity Index; FAS = Functional Ambulatory Scale; FIM = Functional Independence Measure; FM = Fugl-Meyer; FMA-UL = Fugl-Meyer Assessment Upper Limb; FRT 16 = Functional Reach Test 16; FTHUE = Functional Test for the Hemiplegic Upper Extremity; HADS = Hospital Anxiety and Depression Scale; HAMD = Hamilton Depression Rating Scale; IG = intervention group; JTT = Jebsen–Taylor hand function test; K-MBI = Korean version of the Modified Barthel Index; K-MMSE = Korean version of the Mini-Mental State Examination; K-MoCA = Korean-Montreal Cognitive Assessment; MAL = Motor Activity Log; MAS = Modified Ashworth Scale; MBI = Modified Barthel Index; MFT = manual function test; MMDT = Minnesota Manual Dexterity Test; MMSE = Mini Mental State Examination; MoCA = Montreal Cognitive Assessment; mRS = Modified Rankin Scale; MVPT-3 = Motor-Free Visual Perception Test, 3rd edition; NHP = Nottingham Health Profile; NIHSS = National Institute of Health Severity Scale; OWA = Outdoor Walking Assessment; POMA = Performance-oriented mobility assessment; PPT = Purdue pegboard test; SIS = Stroke Impact Scale; SSEQ = Stroke Self-Efficacy Questionnaire; SSQOL = Stroke-Specific Quality of Life; SUS = System Usability Scale; TSRQ-15 = Treatment Self-Regulation Questionnaire-15; TUG = Timed up and go; USER-P = Utrecht Scale for Evaluation of Rehabilitation-Participation; VAS = visual analogue scale; WAIS III = Wechsler Adult Intelligence Scale III; WMFT = Wolf Motor Function Test

*3.3* *Characteristics of participants*

Among the 34 included studies, the highest average age reported for participants in the intervention and control groups was 69.2 years (Lee et al., 2016) and 69.8 years (Ozen et al., 2021), respectively. Twenty-three studies specifically mentioned the inclusion of participants with both ischemic and hemorrhagic stroke. Conversely, 11 studies did not specify the type of stroke experienced by the participants (Amin et al., 2024; Dabrowska et al., 2025; da Silva Ribeiro et al., 2015; Kim, 2018; Latif et al., 2025; Marques-Sule et al., 2021; Oh et al., 2019; Park et al., 2021; Shin et al., 2015; Song & Lee, 2021; Térémetz et al., 2022). The phases of stroke in the included studies were categorized into acute, subacute, and chronic. The subacute phase is identified as occurring 15 to 180 days following the initial stroke, while the chronic phase begins at 180 days post-stroke, marked by typically slow or no clinical progress (Amman et al., 2014). One study focused on acute stroke survivors (Park et al., 2021). Nine studies focused on subacute stroke survivors (Amin et al., 2024; Cano-Mañas et al., 2020; de Rooij et al., 2022; Laffont et al., 2020; Lam et al., 2022; Mazher et al., 2025; Rémy-Néris et al., 2021; Rodríguez-Hernández et al., 2021; Şimşek & Çekok, 2016). Thirteen studies examined chronic stroke survivors (Allegue et al., 2022; da Silva Ribeiro et al., 2015; Huber et al., 2025; Kilinc et al., 2023; Kim, 2018; Kuo et al., 2023; Lee et al., 2017; Lee et al., 2016; Marques-Sule et al., 2021; Oh et al., 2019; Shin et al., 2015; Song & Lee, 2021; Térémetz et al., 2022). Four studies included participants across both acute and subacute phases (Adie et al., 2017; Ali et al., 2024; Choi et al., 2014; Dabrowska et al., 2025), and three studies included participants across both subacute and chronic phases (Ozen et al., 2021; Park & Ha, 2023; Velmurugan et al., 2023). Three studies did not specify the phase of stroke (Faria et al., 2016; Latif et al., 2025; Shin et al., 2016). Long et al. (2020) included stroke survivors with onset time ≤ 1 year.

*3.4* *Characteristics of interventions*

*3.4.1 Setting and providers*

Game-based interventions were predominantly conducted at rehabilitation centers or hospitals. Two studies reported that the interventions were conducted at participants’ homes (Adie et al., 2017; Allegue et al., 2022). Velmurugan et al. (2023) conducted the intervention in a college. Seven studies did not specify the intervention settings (da Silva Ribeiro et al., 2015; Kim, 2018; Kuo et al., 2023; Lee et al., 2016; Oh et al., 2019; Park et al., 2021; Shin et al., 2015).

All interventions were provided by either physical therapists, physiotherapists, or occupational therapists, with the following exceptions: one study was remotely monitored by a clinician (Allegue et al., 2022); one study used a psychologist to provide cognitive rehabilitation (Faria et al., 2016); and one used a trained movement scientist (Huber et al., 2025). No information was provided on the interventionist for two studies (Lee et al., 2016; Mazher et al., 2025), and another two studies only mentioned ‘therapist’ as the interventionist (Long et al., 2020; Park et al., 2021).

*3.4.2. Types of VR-based rehabilitation*

Twenty-six studies utilized non-immersive VR without head-mounted devices, two studies used semi-immersive VR (de Rooij et al., 2022; Shin et al., 2015), and four studies employed immersive VR (Amin et al., 2024; Dabrowska et al., 2025; Park & Ha, 2023; Song & Lee, 2021). The level of immersion was unable to be determined in two studies due to insufficient information (Latif et al., 2025; Velmurugan et al., 2023).

Of the included studies, 13 used mainstream entertainment or consumer gaming systems, such as the Microsoft Kinect (Lee et al., 2017), Nintendo Wii (Adie et al., 2017; Choi et al., 2014; da Silva Ribeiro et al., 2015; Kim, 2018; Marques-Sule et al., 2021; Şimşek & Çekok, 2016; Térémetz et al., 2022; Velmurugan et al., 2023), Oculus Quest 2 (Amin et al., 2024; Dabrowska et al., 2025), and Xbox 360° (Cano-Mañas et al., 2020; Mazher et al., 2025). Another 15 studies used non-commercial or dedicated rehabilitation gaming platforms and devices, including the AbleX (Lam et al., 2022), ArmAble (Ali et al., 2024), Armeo Spring (Rémy-Néris et al., 2021), Dividat Senso (Huber et al., 2025), Doctor Kinetic (Long et al., 2020), Hand Tutor glove and 3Dtutor (Rodríguez-Hernández et a., 2021), Jintronix (Allegue et al., 2022), JoyStim (Oh et al., 2019), and PABLO (Kuo et al., 2023), RAPAEL Smart Glove (Park et al., 2021; Shin et al., 2016), Rehabilitation Joystick for Computerized Exercise (Rejoyce) (Ozen et al., 2021), Reh@City (Faria et al., 2016), Thera-Trainer Balo (TTB) (Kilinc et al., 2023), RehabMaster (Shin et al., 2015).

Four studies did not specify whether the gaming platform was commercial or non-commercial (Laffont et al., 2020, Latif et al., 2025; Lee et al., 2016; Park & Ha, 2023). Song and Lee (2021) used custom VR content that was developed by a professor, delivered through the DK2 Oculus Rift and Oculus Rift controller to provide immersive experience. Finally, one study delivered the VR-based intervention through the Gait Real-time Analysis Interactive Lab (GRAIL) (de Rooij et al., 2022).

*3.4.3. Delivery dose*

Among the studies reviewed, individual session lengths ranged from a minimum of 15 minutes (Laffont et al., 2020) and to a maximum of 60 minutes (Ali et al., 2024; Rémy-Néris et al., 2021; Şimşek & Çekok, 2016; Térémetz et al., 2022). The frequency of interventions varied from daily (Adie et al., 2017) to only twice a week (da Silva Ribeiro et al., 2015; de Rooij et al., 2022; Huber et al., 2025; Kuo et al., 2023; Lam et al., 2022; Lee et al., 2017; Marques-Sule et al., 2021), while the overall duration of interventions ranged from two weeks (Ali et al., 2024) to 12 weeks (Huber et al., 2025; Kim, 2018). Notably, the most common intervention duration was four weeks (n = 10) among the included studies.

*3.4.4. Application of neurorehabilitation principles or theoretical frameworks*

There are 15 principles of neurorehabilitation post-stroke, guiding the design and implementation of rehabilitation interventions that enhance optimal brain recovery and functional improvement. According to Maier et al. (2019), these include: 1) repetitive practice, 2) spaced practice, 3) dosage, 4) task-specific practice, 5) goal-oriented practice, 6) variable practice, 7) increasing difficulty, 8) multisensory stimulation, 9) rhythmic cueing, 10) explicit feedback/knowledge of results, 11) implicit feedback/knowledge of performance, 12) modulate effector selection, 13) action observation/embodied practice, 14) motor imagery, and 15) social interaction.

All studies included in this review explicitly reported the dosage of the game-based intervention. Almost all studies implemented game-based interventions with progressively increasing difficulty, with the exception of eight studies (Adie et al., 2017; Choi et al., 2014; Dabrowska et al., 2025; Latif et al., 2025; Lee et al., 2016; Mazher et al., 2025; Song & Lee, 2021; Térémetz et al., 2022). Twelve studies provided explicit or implicit feedback to participants (e.g., visual feedback, verbal feedback) (Allegue et al., 2022; Amin et al., 2024; de Rooij et al., 2022; Faria et al., 2016; Kuo et al., 2023; Lee et al., 2016; Lee et al., 2017; Rodríguez-Hernández et a., 2021; Shin et al., 2015; Shin et al., 2016; Şimşek & Çekok, 2016; Térémetz et al., 2022). Eight studies specifically highlighted the use of repetitive practice within their game-based interventions (Ali et al., 2024; Cano‑Mañas et al., 2020; Ozen et al., 2021; Park et al., 2021; Rémy-Néris et al., 2021; Rodríguez-Hernández et a., 2021; Shin et al., 2016; Velmurugan et al., 2023). Three studies reported goal-oriented practice (da Silva Ribeiro et al., 2015; de Rooij et al., 2022; Faria et al., 2016). Notably, da Silva Ribeiro et al. (2015) selected Nintendo Wii games to achieve goals similar to those targeted in conventional rehabilitation.

The majority of studies did not incorporate a theoretical framework in the design or implementation of their game-based interventions. Notably, Allegue et al. (2022) and Park and Ha (2023) underpinned their VR-based interventions with self-determination theory (SDT) and self-efficacy theory, respectively. The former integrated SDT principles into motivational interviewing. Similarly, Kuo et al. (2023) and Lee et al. (2017) designed their VR game training protocols based on motor learning theory. In contrast, the remaining studies reviewed did not specify the use of any theoretical framework in the development of their interventions.

*3.5 Feasibility and safety outcomes*

Across 29 of the 34 included RCTs that reported screening figures, the participation rate ranged from 25.4% (Térémetz et al., 2022) to 100% (Allegue et al., 2022; Amin et al., 2024; de Rooij et al., 2022; Faria et al., 2016; Laffont et al., 2020; Oh et al., 2019, Ozen et al., 2021; Park & Ha, 2023; Park et al., 2021; Rodríguez-Hernández et a., 2021; Song & Lee, 2021) with a mean of approximately 84% and a median close to 95%. Most trials exceeded 80% participation among eligible patients, suggesting that, when offered, game‑based interventions were acceptable to the majority of stroke survivors. Five studies were clear outliers: Adie et al. (2017), Huber et al. (2025), Lee et al. (2016), Lee et al. (2017), and Shin et al. (2016) reported participation rate of 71%, 28.8%, 66.7%, 71.4%, and 64.8%, respectively. Térémetz et al. (2022) only had 25%, primarily due to a high refusal rate among otherwise eligible patients.

Retention after enrolment was generally good. Overall attrition ranged from 0% to 50%, with a mean of about 9% and a median of around 7%. All but one trial (Shin et al., 2016) reported withdrawal rates below 30%, a threshold commonly used to indicate feasible retention, and several trials (Amin et al., 2024; Choi et al., 2014; da Silva Ribeiro et al., 2015; Faria et al., 2016; Kim, 2018; Latif et al., 2025; Marques-Sule et al., 2021; Mazher et al., 2025; Park et al., 2021; Song & Lee, 2021; Velmurugan et al., 2023) reported no dropout. Attrition rates were broadly similar between game‑based and control arms, indicating that VR‑based programs were at least as acceptable as conventional therapy once participants had agreed to take part.

Adherence or engagement with the additional game‑based components was explicitly quantified in four trials (Ali et al., 2024; Allegue et al., 2022; Cano‑Mañas et al., 2020; Laffont et al., 2020). In these studies, adherence ranged from 83% to 95% of the prescribed dose, with three of the four reporting values above 90%. Although adherence was under‑reported overall, the available data suggest that participants who initiated game‑based or home‑based training were generally able to complete most of the planned sessions or practice time, surpassing the ≥75% engagement threshold often used to indicate acceptable usability.

Adverse events were described in varying detail. Adie et al. (2017) and Rémy‑Néris et al. (2021) reported a substantial number of serious adverse events (e.g. recurrent stroke, hospital readmission), but none were judged to be related to the trial interventions. Huber et al. (2025) reported that two participants withdrew during the intervention period due: one due to a non-casually related adverse event, and one due to an unrelated serious adverse event. When adverse events were broken down by group, their frequency and profile were similar in the intervention and control arms. Laffont et al. (2020) documented eight events possibly related to therapy (shoulder pain, complex regional pain syndrome, worsening spasticity), again distributed across both groups. Lee et al. (2017) reported 19 adverse events in the VR group versus 30 in the standard treatment group, consisting mainly of transient soreness, increased muscle tone, dizziness and pain that resolved with rest. Similarly, de Rooij. (2021) reported that 11 participants in the VR group and 15 participants in the conventional therapy group experienced events such as near-falls, dizziness, pain, and fatigue. Kuo et al. (2023) also noted shoulder soreness and transient dizziness in both groups.

In the remaining trials, either no adverse events were observed, or they were described as unrelated medical events or as minor symptoms leading to discontinuation in isolated cases. Importantly, no study reported a technology‑related serious adverse event, and VR‑based interventions were generally considered safe when delivered within structured rehabilitation programs.

*3.6 Quality appraisal*

Overall, the Cochrane ROB 2.0 rated two studies with an overall “low” risk of bias, 19 studies with “some concerns” and the remaining 13 studies as a “high” overall bias (Fig. 2). Studies rated as “high” were mostly due to data presented, such as domain results reported instead of the total score, differences from baseline between groups and no data table provided (Kim, 2018). Adie et al. (2017) and Lee et al. (2017) analyzed results based on the initial allocated number of participants when the availability of data was less than 95%, influencing their true value. Adie et al. (2017) and Kim (2018) had multiple eligible outcome measurements with the use of two different scales for the same outcome. Some studies were unable to be blinded since knowledge of interventions was required. The GRADEpro software rated the QoL outcome of low quality, the upper extremity functions as low to very low quality, and explanations are provided in Supplementary Material: Fig. S4.

[Insert Figure 2]

Fig. 2. Risk of bias assessment of included studies using the Revised Cochrane Risk of Bias Assessment Tool for Randomized Trials (RoB2).

*3.7 Effectiveness of game-based interventions*

*3.7.1 Quality of life*

The QoL was assessed using the Stroke Impact Scale (SIS) (n = 13) (Adie et al., 2017; Ali et al., 2024; Allegue et al., 2022; de Rooij et al., 2022; Faria et al., 2016; Huber et al., 2025; Kim, 2018; Kuo et al., 2023; Lee et al., 2017; Mazher et al., 2025; Rémy-Néris et al., 2021; Shin et al., 2016; Térémetz et al., 2022), Short Form 36 (SF-36) (n = 5) (da Silva Ribeiro et al., 2015; Kilinc et al., 2023; Laffont et al., 2020; Lam et al., 2022; Shin et al., 2015), SF-12 (n = 1) (Park & Ha, 2023), EuroQoL (EQ-5D) (n = 2) (Cano-Mañas et al., 2020; Rémy-Néris et al., 2021), EQ-5D-5L (n = 1) (Rodríguez-Hernández et a., 2021), Stroke-specific QoL (SS-QoL) (n = 3) (Amin et al., 2024; de Rooij et al., 2022; Ozen et al., 2021), WHODAS 2.0 (Dabrowska et al., 2025), and Nottingham Health Profile (NHP) (n = 1) (Şimşek & Çekok, 2016). Meta-analysis with random effects model for QoL was performed for the six studies involving 493 participants using SIS (Fig. 3). The pooled results showed a non-statistically significant small effect favoring conventional therapy (SMD = -0.09, 95% CI: -0.30-0.11, *p* = .36) with non-statistically significant heterogeneity ($I^{2}$ = 15%, *p* = .32).

To explore potential sources of heterogeneity across studies, subgroup analyses were conducted based on: 1) neurorehabilitation principle (feedback versus no feedback), 2) intervention components (VR-based versus a combination of VR and conventional therapy/usual care), and 3) type of VR system/device (mainstream entertainment/consumer gaming system versus non-commercial/dedicated rehabilitation gaming platform/device). The pool effect estimates for the intervention group compared to control remained non-statistically significant across all subgroups (*p* > .05) (Supplementary Material, Fig. S1). However, moderate to substantial heterogeneity was observed in some subgroups (*I*^2^ = 33%, 62%).

Sensitivity analyses were performed to assess the robustness of the primary findings. First, a sensitivity analysis excluding all three studies rated as having high risk of bias (Adie et al., 2017; Kim, 2018; Lee et al., 2017) yielded results consistent with the primary analysis (primary: SMD = -0.09, 95% CI: -0.30-0.11, *p* = .36 versus sensitivity: SMD = -0.06, 95% CI: -0.52-0.40, *p* = .81) [Supplementary Material, Fig. S1 (d)]. Heterogeneity increased from *I^2^* = 15% (*p* = .32) to *I^2^* = 62 (*p* = .07). Second, a sensitivity analysis excluding only Shin et al. (2016), the study with the highest attrition rate from the primary analysis also yielded consistent results, with heterogeneity reduced from *I^2^* = 15% (*p* = .32) to *I^2^* = 0% (*p* = .7) [Supplementary Material, Fig. S1 (e)]. When Shin et al. (2016) was removed (highest attrition rate) from the subgroup analysis comparing ‘mainstream entertainment/consumer gaming system’ versus ‘non-commercial/dedicated rehabilitation gaming platform/device’, there remained no statistically significant differences between both subgroups, with heterogeneity of *I^2^* = 0% and *I^2^* = 25% [Supplementary Material, Fig. S1 (f)].

Narratively summarizing, the findings on QoL across included studies are mixed. Amin et al. (2024) found that the immersive VR game intervention led to significant improvement in QoL compared to the control group at the 9th week follow-up, following a six-week intervention (*p*<.001). Similarly, Kim (2018), Mazher et al. (2025), Shin et al. (2016), and Şimşek & Çekok (2016) reported significant improvements in QoL at post-intervention with the use of game-based interventions (all *p*-values of <.05). Huber et al. (2025) found significant interaction effects favoring the exergame group, as measured by SIS 3.0 scores. Rodríguez-Hernández et a. (2021) reported that, at post-intervention, the intervention group demonstrated significantly better EQ-5D-5L scores across all dimensions (except pain/discomfort) compared to control group.

Two studies reported significant effects in specific QoL domains favoring the game-based intervention. Cano-Manãs et al. (2020) found significant improvements in the domains of anxiety or depression (*p* <.01), pain or discomfort (*p* <.01), and the visual analogue scale (*p* <.01). Shin et al. (2015) reported significant improvement s in the domain of role limitation due to physical problems (*p* =.031). However, the latter finding contrasts with da Silva Ribeiro et al. (2015), where physical functioning outcomes favored conventional therapy over the game-based intervention.

Eight studies reported no significant difference between groups at post-intervention (all *p* values > .05) (Faria et al., 2016; Kilinc et al., 2023; Kuo et al., 2023; Laffont et al., 2020; Lam et al., 2022; Ozen et al. (2021), Rémy-Néris et al. (2021) and Térémetz et al., 2022). Inconclusive findings were also reported. Allegue et al. (2022) found inconsistent results for the intervention group across four time points using the SIS-16 scores, whereas the control group showed improvements in activities of daily life, hand function, and mobility. Park and Ha (2023), using the SF-12, reported significant group-by-time interactions in health related QoL favoring the intervention group.

[Insert Figure 3]

Fig. 3. Forest plots: quality of life

*3.7.2 Upper extremity functions*

The pooled results from nine studies (Fig. 4a) using the Fugl-Meyer Assessment for Upper Extremity (FMA-UE), including 466 participants, demonstrated that game-based interventions did not yield statistically significant improvements in upper extremity functions compared to control groups (SMD = 0.56, 95% CI: -0.02-1.15, *p* = .06). The analysis exhibited considerable statistical heterogeneity ($I^{2}$ = 88%, *p* < .01), indicating substantial variability among the included studies. Subgroup analyses were conducted comparing 1) feedback versus no feedback in terms of neurorehabilitation principles, 2) stroke phase: chronic stroke versus acute or acute/subacute stroke, 3) training frequency, and 4) interventions delivered using consumer gaming system versus non-commercial/dedicated rehabilitation gaming platform/device. The findings are consistent with the primary analysis, where there were no statistically significant differences observed across the subgroup analyses (Supplementary Material Fig.S2 (a)-(d). Post-hoc sensitivity analyses were attempted to explore the effects by removing outliers (studies with high risk of bias/attrition rates). However, results remained similar to the primary and subgroup analyses (Supplementary Material Fig.S2 (e)-(h).

A random-effects meta-analysis of four studies (N = 470) examining upper extremity function using the Action Research Arm Test (ARAT) yielded a pooled SMD of 0.15 (95% CI: -0.22-0.52, *p* =.41), with substantial heterogeneity (*I^2^* = 73%, *p* = .01) (Fig. 4b). Sensitivity analysis excluding the sole study using immersive virtual reality (the other three studies using non-immersive VR) did not change the statistical significance of the result (Supplementary Material Fig. S3).

A narrative synthesis of five studies, which lacked sufficient data for meta-analysis, corroborated these findings. Studies reported no significant differences between game-based interventions and control groups in upper extremity motor function, as measured by standardized assessments such as the FMA-UE, ARAT, and the Motor Function Test (MFT). Rémy-Néris et al. (2021) observed no significant between-group differences in FMA-UE (*p*=.22) and ARAT (*p* = .074) scores following a four-week intervention. Consistent findings were reported by Térémetz et al. (2022), with no significant group differences in FMA-UE (*p* = .43) and ARAT (*p* = .93) scores. Similarly, three additional studies reported no significant between-group differences in upper extremity function following the interventions, as measured by the FMA-UE (Choi et al., 2014; Kuo et al., 2023; Long et al., 2020; Shin et al., 2015), or by MFT (Kim, 2018; Song & Lee, 2021). da Silva Ribeiro et al., 2015 reported that there were no significant differences between groups in FM scores across seven domains, including upper limb motor function where post-intervention scores were M = 44.7 (SD = 14.2) in intervention group versus M = 38.7(SD = 19.6) in control group, *p* = .486. Allegue et al. (2022) reported that both intervention and control groups had 50% (two out of four participants in each group) demonstrated an improvement change scores in FMA-UE, which was maintained over time. Similarly, Oh et al. (2019) reported that both intervention and control groups demonstrated significant improvements in FMA-UE. However, one study reported that the intervention group showed significant improvement in hand functional status compared to control group at post-intervention (*p* < .05) (Latif et al., 2025).

Song and Lee (2021) reported there was no statistically significant difference observed in upper extremity sensory functions (two-point discrimination and stereognosis tests) in the affected arm between group at the end of the four weeks training, except for proprioception test which reported to be statistically significant different between intervention and control groups (*p* = .04).

Regarding real-world arm use, as assessed by the Motor Activity Log, no significant between-group differences were found by Adie et al. (2017) at a six-month follow-up or by Térémetz et al. (2022) at one-week post-therapy. In contrast, Allegue et al. (2022) reported that all participants in the intervention group exhibited improvement from baseline to post-intervention, compared to 80% improvement in the control group.

Muscle strength in the upper arm, as measured by the Motricity Index (MI), was also evaluated. Ali et al. (2024) found no significant differences between groups after two weeks of game-based intervention compared with task-based training [−2.5 (95%CI: −5.8, 0.8); Cohen’s d = 0.19; *p* = .143].

Analyses of hand function revealed some differential effects. Shin et al. 2016), employing the Jebsen–Taylor hand function test (JTT) and Purdue pegboard test (PPT), found that the intervention group demonstrated significant improvements in the JTT-total (F = 4.073, df = 1.497, *p* = .032) and JTT-gross (F = 4.155, df = 1.705, *p* = .025) scores, as reflected in significant Time × Group interactions. However, no significant differences between groups were observed in fine hand motor function (Shin et al., 2016). In terms of hand dexterity, two studies reported that the intervention group demonstrated significantly greater improvement in the Box and Block Test (BBT) scores when compared to the control group (Amin et al., 2024; Kuo et al., 2023).

[Insert Figure 4]

Fig. 4. Forest plots: (a) upper motor functions measured by Fugl Meyer, (b) upper motor functions measured by ARAT

**4. Discussion**

This review evaluated the characteristics and effectiveness of VR-based interventions on QoL and upper extremity motor functions among stroke survivors. Thirty-four randomized controlled trials encompassing 1,834 participants were included. The interventions varied in terms of setting, technology platforms, dose, and theoretical underpinnings. Our feasibility analyses indicated that VR-based interventions were generally acceptable and safe: participation among eligible patients typically exceeded 80%, attrition rates were usually below 20%, and adherence to the prescribed VR or game-based components was above 80% in the few trials that reported it (Bovim et al., 2025; Su et al., 2024). However, despite this encouraging feasibility profile, the effects on QoL and upper extremity functions showed no significant advantage over conventional rehabilitation.

The meta-analysis revealed that VR-based interventions had a non-significant effect on QoL as measured by SIS, with inconsistencies observed across narrative synthesis findings. Our meta-analysis on QoL contrasts with a prior review reported by Domínguez-Téllez et al. (2020), which reported mixed outcomes for QoL, inconclusive results when assessed via the Modified Barthel Index (MBI), but favorable effects when measured with the Functional Independence Measure (FIM). However, it is important to note that neither the MBI nor the FIM is specifically designed to measure quality of life among stroke survivors, but can be used for patients with various conditions causing physical disability. One of the reasons for the non-significant effects could be the fact that our review conducted the pooled effects for pre- and post-intervention scores, which were of short duration. Furthermore, Bartlova et al. (2022) suggest that QoL is the worst among patients with the shortest time since their last stroke, whereas it improves with the time that has passed since the event.

Our findings suggest that VR interventions yield comparable outcomes to conventional therapy for improving upper extremity functions in stroke survivors, with no significant difference between groups. This contrasts with the meta-analysis by Domínguez-Téllez et al. (2020), which reported favorable results of VR interventions on UL motor function (Fugl-Meyer Assessment for upper extremity, standardized mean difference [SMD] = 1.53, 95% CI [0.51–2.54], *p* = .003). While their review pool had nine studies (year 2009-2016; sample sizes between 14 and 68 participants), the cited study is absent ‘Rubio (2016)’. Domínguez-Téllez et al. (2020) rated the included studies as methodologically robust using the PEDro scale. Further supporting the potential of VR, Wu et al. (2021) conducted a meta meta-analysis on six studies which reported to have satisfactory methodological quality and found that VR-based interventions significantly improved upper extremity functions, measured by FMA-UE, with a large effect size (SMD 4.606, 95% CI: 2.733-6.479, *p* <.05), albeit with considerable heterogeneity (*I*^2^ = 92.045) (Wu et al., 2021). Similarly, Soleimani et al. (2024) conducted a review with 34 RCTs and demonstrated VR’s significant advantage over conventional occupational therapy (SMD 0.63, 95% CI 0.33–0.92, *p* <.001), though heterogeneity remained (*I*^2^ = 82%) (Soleimani et al., 2024). The divergence between our findings and prior reviews may stem from temporal shifts in conventional rehabilitation standards. Notably, previous reviews included some studies that were conducted more than a decade ago. Stroke rehabilitation guidelines have evolved to recommend higher amounts (Stinear et al., 2020). Additionally, most VR interventions in our review were non-immersive (with only semi-immersive and one fully immersive VR intervention), whereas Kiper et al. (2023) reported that fully immersive VR significantly improved overall upper extremity functions as measured by FMA-UE (MD 6.33, 95% CI: 4.15-8.50, *I*^2^ = 25%, *p* <.001). This suggests immersion level critically influences outcomes, as fully immersive systems better simulate real-world tasks, thereby optimizing motor relearning.

Despite the meta-analyses of our intended outcomes revealing no statistically significant effects of VR-based interventions on QoL and upper extremity functions, these findings do not automatically imply clinical irrelevance. Interpreting these findings in the context of minimal clinically important differences (MCIDs) will provide additional insights into whether the observed effect sizes are large enough to be perceived as meaningful for stroke survivors. For the QoL, the pooled standardized mean difference of -0.09 (favoring control group) fell below established MCID thresholds by Lin et al. (2010) for SIS physical domains. Authors reported that the mean change scores must reach 9.2 points on the strength subscale, 5.9 points on ADL/IADL, 4.5 points on mobility, and 17.8 points on hand function to be regarded as clinically important improvements. This indicates that the magnitude of benefit may remain below what patients perceive clinically meaningful, which may require substantial modification of the intervention protocols. Nonetheless, the different versions of SIS used across included studies may have contributed to heterogeneity in the pooled estimate. Future studies should standardize the SIS version.

For the upper extremity functions, the pooled SMD of 0.56 approached statistical significance (*p* =.06) and exceeded commonly cited MCID thresholds for upper extremity motor recovery in stroke rehabilitation. Page et al. (2012) estimated the clinical important difference for the upper extremity Fugl Meyer to be 4.25 to 7.25 points in chronic stroke survivors using the ROC analysis, where the CID corresponds to an approximate SMD range of 0.25 to 0.60. Hence, the observed SMD of 0.56 falls within this range, suggesting that the effect, while imprecise due to wide confidence intervals and very low certainty evidence, may be clinically meaningful in some patients or settings. In contrast, the effect using ARAT (SMD = 0.15) fell below typical MCID thresholds for upper extremity function. Lang et al. (2008) reported MCID values for the ARAT of 12-17 points in the early subacute phase after stroke, while Van der Lee et al. (2001) established that ARAT can detect a clinically relevant difference of 5.7 points. Thus, these raw score thresholds correspond to SMDs of approximately 0.2 to 0.6, suggesting that the magnitude of improvement observed in this review is unlikely to be clinically detectable.

Outcome measure heterogeneity further complicates the interpretation of the pooled estimates. Nine different QoL were employed across studies, with only six RCTs being eligible for SIS-based pooling. The FMA-UE and ARAT assess fundamentally different aspects of motor recovery, neurological impairment versus functional task performance, and their differential sensitivity to change may partly explain the discrepancy in effect sizes and the considerable heterogeneity observed (*I^2^* = 88% and *I^2^* = 73%, respectively). Future trials should adopt a consensus-based core outcome set to enable more reliable synthesis.

The null findings may also reflect insufficient intervention intensity. The most common duration was four weeks (n = 10), with sessions of 20-60 minutes delivered two to five times weekly, below high repetition, task specific volumes recommended by current stroke rehabilitation guidelines (Kwakkel et al., 2023; O’Flaherty & Ali, 2024). Furthermore, the predominance of non-immersive VR (n = 26 of 34 studies) may have limited sensorimotor engagement; Kiper et al. (2023) reported that fully immersive VR yielded significant FMA-UE improvements (MD = 6.33, 95% CI: 4.15-8.50, *I^2^* = 25%), suggesting that immersion level critically influences outcomes. Collectively, these observations suggest the null findings may reflect study design constraints rather than an inherent ceiling on the therapeutic potential of VR.

A notable challenge in interpreting our pooled estimates is the considerable statistical heterogeneity observed across UE outcomes. While we conducted sensitivity analyses to assess the robustness of the findings, the sources of this heterogeneity were not fully explored. Several factors are likely to have contributed to the observed variability. First, the included studies enrolled patients at different stages of stroke recovery (acute to chronic phases). Chronicity is known to influence the trajectory of spontaneous neurological recovery and the responsiveness to rehabilitation interventions (Langhorne et al., 2011; Stinear et al., 2020). Stroke survivors in the subacute phase may exhibit greater capacity for improvement due to the natural recovery processes, whereas those in the chronic phase may show more modest gains but potentially greater differentiation between intervention and control groups. Thus, the pooling of studies across these distinct recovery stages may have diluted or exaggerated the observed effect sizes, contributing to the high *I*^2^ values. Second, the intervention characteristics varied including total dose, session frequency, duration of intervention, and the nature of the VR systems used. As noted earlier, fully immersive systems appear to yield larger effects (Kiper et al., 2023), suggesting that technological immersion is a key moderator. However, only one study in this review employed fully immersive VR, limiting the ability to conduct subgroup analyses by immersion level. Third, the control interventions were not uniform across studies, introducing further variability into the pooled comparisons. This lack of standardization in both arms is a well-recognized source of heterogeneity in rehabilitation systematic reviews. For instance, Pollock et al. (2014) highlighted that evidence related to the dose of interventions is limited by substantial heterogeneity and noted that a lack of high-quality evidence prevents robust comparisons of interventions. Additionally, future reviews with larger numbers of studies should consider meta-regression to systematically examine these moderators and better understand the effectiveness of VR-based interventions on patient outcomes.

Beyond clinical effectiveness, our descriptive synthesis of safety data further supports the overall safety of game-based rehabilitation. Although adverse events were not the focus of our primary analyses, it is important to extract these data descriptively to address potential safety concerns that clinicians might have when considering VR-based interventions. Across the trials that systematically reported adverse events, serious events (such as recurrent stroke or unplanned hospitalization) occurred in both arms and were consistently judged to be unrelated to the VR or game-based components (Adie et al., 2017; Rémy‑Néris et al., 2021). Where detailed information was available, most adverse events were mild and transient (typically musculoskeletal soreness, increased muscle tone, dizziness, or fatigue) and resolved after rest without lasting consequences (Kuo et al., 2023; Laffont et al., 2020; Lee et al., 2017). In some studies, the control groups experienced equal or greater numbers of such events compared with the VR groups (Laffont et al., 2020; Lee et al., 2017). Taken together, these data suggest that, when appropriately monitored, VR-based interventions have a safety profile comparable to that of conventional rehabilitation (Choi et al., 2016; Norouzi-Gheidari et al., 2021).

At the same time, several trials emphasized the importance of therapist guidance to ensure safe and effective use of game-based systems. Şimşek and Çekok (2016) reported that some patients using Nintendo Wii exhibited compensatory trunk and shoulder movements, postural imbalance, and increased muscle tone, leading the authors to recommend continuous physiotherapist supervision, particularly for individuals with persistent balance problems. Similarly, Lee et al. (2017) and Kuo et al. (2023) described how therapists adjusted task difficulty and posture in response to emerging soreness or dizziness. These observations highlight that VR-based training is not inherently “self-correcting”; without adequate supervision, there is a risk of reinforcing maladaptive movement patterns even in the absence of overt adverse events (Hung et al., 2014; Jandaghi et al., 2021). For home-based or minimally supervised programs, integrating initial supervised training, remote monitoring, and clear safety guidelines may therefore be essential (Proffitt & Lange, 2015; Winstein et al., 2016).

Among the 34 RCTs included in our review, only four were underpinned by theory, and only one of these contributed to the meta-analysis of QoL, and one to upper extremity functions. This limited theoretical integration into intervention development represents a significant gap, given that theory-based interventions are critical for targeting the underlying mechanisms of behavior change (Michie et al., 2008). Furthermore, beyond ensuring design efficacy, theory-driven interventions facilitate theory testing, allowing refinement of behavioral models (Craig et al., 2008) and improving our understanding of the factors that influence outcomes (Nilsen, 2015). The scarcity of such theoretically informed trials in our review may explain the inconsistent findings in QoL and upper extremity functions across studies.

*Limitations*

This review has several limitations that should be considered when interpreting the findings. First, the meta-analyses incorporated studies with considerable heterogeneity, particularly in intervention characteristics. This heterogeneity challenges the generalizability and comparison of the effects of the interventions. Second, the majority of included studies were rated as having either ‘some concerns’ or ‘high’ risk of bias in methodological quality. Consequently, with the overall certainty of evidence assessed using the GRADE criteria was rated as ‘low’ and ‘very low’ for outcomes (QoL and upper extremity functions), limiting confidence in the pooled estimates. Third, our analyses focused on short-term outcomes (baseline and immediately post-intervention), which restricts the ability to evaluate long-term effects or the sustainability of benefits. Lastly, our search was restricted to articles published in English, which potentially introduces language bias by excluding relevant articles published in other languages.

*Implications for practice and research*

Although the pooled effects of VR-based interventions were not statistically significant, these interventions nevertheless show promise as a complementary approach to conventional rehabilitation, particularly for mitigating access barriers such as distance and transportation. Given that the overall certainty of evidence was rated as low and very low, the clinical implications of our findings must be interpreted with considerable caution. The pooled analyses did not demonstrate a significant advantage of VR-based interventions over conventional rehabilitation. As such, VR-based interventions are not a replacement for conventional therapy in routine stroke care. However, VR-based interventions may still hold value as a complementary or adjunctive option, particularly for stroke survivors who face barriers to accessing traditional rehabilitation. To maximize their impact, a collaborative, multidisciplinary approach should be adopted to integrate VR-based interventions, tailoring them to patients’ specific needs, preferences, and technological literacy.

Our feasibility findings, with recruitment generally exceeding 20% of eligible patients and withdrawal rates below 30% in most trials, are consistent with commonly applied feasibility benchmarks and further support the practicality of integrating VR‑based programs into routine stroke care, provided that appropriate clinical supervision and follow-up are in place. Notably, adherence was only quantified in four trials, yet all reported high engagement with the VR or game‑based components (83–95% of the prescribed dose), suggesting good usability among participants who initiated these programs, while also highlighting the need for more consistent reporting of adherence in future trials.

Our review also highlights critical research gaps. First, there is a need for more rigorous study designs, adequately powered RCTs with longer follow-up periods. Future trials should also stratify participants by stroke chronicity and standardize intervention protocols to reduce heterogeneity and enable more meaningful meta-analytic synthesis. Second, the optimal content and design of interventions in the included studies remain unclear due to high heterogeneity and poor evidence quality among existing studies, highlighting the necessity of incorporating patients’ perspectives into the development process to better tailor interventions to their needs. Additionally, most included studies lacked a theoretical framework to guide intervention development. Future research should integrate theoretical models to underpin the design of game-based interventions, thereby facilitating a deeper understanding of the causal determinants (e.g., motivation/adherence to intervention).

**5. Conclusion**

This review found that VR-based interventions do not confer superior benefits over conventional rehabilitation for improving QoL or upper extremity functions in stroke survivors. The overall certainty of the evidence was rated as low to very low, substantially limiting confidence in the estimated effects. Consistent with this, the review identified several critical gaps in the literature, including methodological heterogeneity, limited use of theoretical frameworks, and a lack of long-term follow-up data. These gaps underscore the need for rigorously designed, adequately powered RCTs with greater theoretical underpinning. Although our descriptive synthesis suggested that these interventions are generally feasible and safe, this finding is based on limited reported data, particularly for adherence which was quantified in only a minority of RCTs. As such, the lack of demonstrable clinical effectiveness indicates that VR-based rehabilitation, in its current form should not replace conventional rehabilitation, but may serve as a complementary or adjunctive option, particularly when access to traditional rehabilitation is limited.

**Funding**

This work was supported by the XX Research Fellow Start Up Grant (WBS No. A-0010310-XX-XX). Dr. XX is supported by the Ministry of Health of the XX - conceptual development of research organization (FNBr, 65269XXX).

**Declaration of competing interests**

All authors declare no competing interests.

**Data Availability**

The data underlying this article are available in the article and in its online supplementary material.

**References**

Aderinto, N., AbdulBasit, M. O., Olatunji, G., & Adejumo, T. (2023). Exploring the

transformative influence of neuroplasticity on stroke rehabilitation: a narrative review of current evidence. *Annals of Medicine and Surgery (2012)*, *85*(9), 4425–4432. https://doi.org/10.1097/MS9.0000000000001137

Adie, K., Schofield, C., Berrow, M., Wingham, J., Humfryes, J., Pritchard, C., James,

M., & Allison, R. (2017). Does the use of Nintendo Wii Sports^TM^ improve arm function? Trial of Wii^TM^ in Stroke: a randomized controlled trial and economics analysis. *Clinical Rehabilitation*, *31*(2), 173–185. https://doi.org/10.1177/0269215516637893

Ali, A. S., Kumaran, D. S., Unni, A., Sardesai, S., Prabhu, V., Nirmal, P., Pai, A. R.,

Guddattu, V., & Arumugam, A. (2024). Effectiveness of an intensive, functional, and gamified rehabilitation program on upper limb function in people with stroke (EnteRtain): A multicenter randomized clinical trial. *Neurorehabilitation and Neural Repair*, *38*(4). https://doi.org/10.1177/15459683231222921

Allegue, D. R., Higgins, J., Sweet, S. N., Archambault, P. S., Michaud, F., Miller, W.,

Tousignant, M., & Kairy, D. (2022). Rehabilitation of upper extremity by telerehabilitation combined with exergames in survivors of chronic stroke: preliminary findings from a feasibility clinical trial. *JMIR Rehabilitation and Assistive Technologies*, *9*(2), e33745. https://doi.org/10.2196/33745

Amin, F., Waris, A., Syed, S., Amjad, I., Umar, M., Iqbal, J., & Omer Gilani, S.

(2024). Effectiveness of immersive virtual reality-based hand rehabilitation games for improving hand motor functions in subacute stroke patients. *IEEE Transactions on Neural Systems and Rehabilitation Engineering: A Publication of the IEEE Engineering in Medicine and Biology Society*, *32*, 2060–2069. https://doi.org/10.1109/TNSRE.2024.3405852

Ammann, B. C., Knols, R. H., Baschung, P., de Bie, R. A., & de Bruin, E. D. (2014).

Application of principles of exercise training in sub-acute and chronic stroke survivors: a systematic review. *BMC Neurology*, *14*, 167.

https://doi.org/10.1186/s12883-014-0167-2

Anwer, S., Waris, A., Gilani, S. O., Iqbal, J., Shaikh, N., Pujari, A. N., & Niazi, I. K.

(2022). Rehabilitation of upper limb motor impairment in stroke: A narrative

review on the prevalence, risk factors, and economic statistics of stroke and state of the art therapies. *Healthcare*, *10*(2), 190.

https://doi.org/10.3390/healthcare10020190

Baranyi, R. (2023). DeapSea: Workflow‐supported serious game design for stroke

rehabilitation. *International Journal of Computer Games Technology*, *2023*(1), 3169262.  https://doi.org/10.1155/2023/3169262

Barrett, N., Swain, I., Mecheraoui, C. (2016). The use and effect of video game design

theory in the creation of game-based systems for upper limb stroke

rehabilitation. *Journal of Rehabilitation and Assistive Technologies*

*Engineering,* 3, 2055668316643644.

https://doi.org/10.1177/2055668316643644

Bártlová, S., Šedová, L., Havierniková, L., Hudáčková, A., Dolák, F., & Sadílek, P.

(2022). Quality of life of post-stroke patients. *Zdravstveno Varstvo*, *61*(2), 101-

108. https://doi.org/10.2478/sjph-2022-0014

Béjot, Y., & Yaffe, K. (2019). Ageing population: A neurological

challenge. *Neuroepidemiology*, *52*(1-2),76–77.

https://doi.org/10.1159/000495813

Bovim, L. P. V., Rotevatn, E. Ø., Kvidaland, H. K., Bogen, B., Halvorsen, T., &

Engan, M. (2025). Feasibility of a group-based intervention to enhance health-related quality of life and physical activity in children and adolescents with chronic illness: a study protocol. *Pilot and Feasibility Studies*, *11*(1), 101. https://doi.org/10.1186/s40814-025-01682-w

Campbell, M., McKenzie, J. E., Sowden, A., Katikireddi, S. V., Brennan, S. E., Ellis,

S., Hartmann-Boyce, J., Ryan, R., Shepperd, S., Thomas, J., Welch, V., &

Thomson, H. (2020). Synthesis without meta-analysis (SWiM) in systematic reviews: reporting guideline. *BMJ (Clinical research ed.)*, *368*, l6890. https://doi.org/10.1136/bmj.l6890

Cano-Mañas, M. J., Collado-Vázquez, S., Rodríguez Hernández, J., Muñoz Villena,

1. J., & Cano-de-la-Cuerda, R. (2020). Effects of video-game based therapy

on balance, postural control, functionality, and quality of life of patients with subacute stroke: a randomized controlled trial. *Journal of Healthcare Engineering*, *2020*, 1–11. https://doi.org/10.1155/2020/5480315

Choi, J. H., Han, E. Y., Kim, B. R., Kim, S. M., Im, S. H., Lee, S. Y., & Hyun, C. W.

(2014). Effectiveness of commercial gaming-based virtual reality movement therapy on functional recovery of upper extremity in subacute stroke patients. *Annals of Rehabilitation Medicine*, *38*(4), 485–493. https://doi.org/10.5535/arm.2014.38.4.485

Choi, Y. H., Ku, J., Lim, H., Kim, Y. H., & Paik, N. J. (2016). Mobile game-based

virtual reality rehabilitation program for upper limb dysfunction after ischemic stroke. *Restorative Neurology and Neuroscience*, *34*(3), 455–463. https://doi.org/10.3233/RNN-150626

Coleman, E. R., Moudgal, R., Lang, K., Hyacinth, H. I., Awosika, O. O., Kissela, B.

M., & Feng, W. (2017). Early rehabilitation after stroke: a narrative

review. *Current Atherosclerosis Reports*, *19*(12), 59.

https://doi.org/10.1007/s11883-017-0686-6

Craig, P., Dieppe, P., Macintyre, S., Michie, S., Nazareth, I., Petticrew, M., & Medical

Research Council Guidance (2008). Developing and evaluating complex interventions: the new Medical Research Council guidance. *BMJ (Clinical research ed.)*, *337*, a1655. https://doi.org/10.1136/bmj.a1655

Dabrowská, M., Honzíková, L., Pastucha, D., Janura, M., Tomášková, H., Fiedorová,

I., Čechová, Š. A., Trdá, J., & Elfmark, M. (2025). Virtual reality as a potential therapy in a rehabilitation sanatorium for patients after ischemic stroke: impact on quality of life and social participation-a randomized trial. *Frontiers in Rehabilitation Sciences*, *6*, 1539175. https://doi.org/10.3389/fresc.2025.1539175

Dąbrowská, M., Pastucha, D., Janura, M., Tomášková, H., Honzíková, L., Baníková,

Š., Filip, M., & Fiedorová, I. (2023). Effect of virtual reality therapy on quality of life and self-sufficiency in post-stroke patients. *Medicina*, *59*(9), 1669. https://doi.org/10.3390/medicina59091669

da Silva Ribeiro, N. M., Ferraz, D. D., Pedreira, É., Pinheiro, Í., da Silva Pinto, A. C.,

Neto, M. G., Dos Santos, L. R. A., Pozzato, M. G. G., Pinho, R. S., &

Masruha, M. R. (2015). Virtual rehabilitation via Nintendo Wii® and conventional physical therapy effectively treat post-stroke hemiparetic patients. *Topics in Stroke Rehabilitation*, *22*(4), 299–305. https://doi.org/10.1179/1074935714Z.0000000017

de Rooij, I. J., van de Port, I. G., Punt, M., Abbink-van Moorsel, P. J., Kortsmit, M.,

van Eijk, R. P., ... & Meijer, J. W. G. (2021). Effect of virtual reality gait training on participation in survivors of subacute stroke: a randomized controlled trial. *Physical Therapy*, *101*(5). https://doi.org/10.1093/ptj/pzab051

Domínguez-Téllez, P., Moral-Muñoz, J. A., Salazar, A., Casado-Fernández, E., &

Lucena Antón, D. (2020). Game-based virtual reality interventions to improve

upper limb motor function and quality of life after stroke: Systematic review

and meta-analysis. *Games for Health Journal*, *9*(1), 1–10.

https://doi.org/10.1089/g4h.2019.0043

Faria, A. L., Andrade, A., Soares, L., & I Badia, S. B. (2016). Benefits of virtual reality

based cognitive rehabilitation through simulated activities of daily living: a randomized controlled trial with stroke patients. *Journal of Neuroengineering and Rehabilitation*, *13*(1), 96. https://doi.org/10.1186/s12984-016-0204-z

Fricke, J. & Unsworth, C. A. (1996). Inter-rater reliability of the original and modified

Barthel Index, and a comparison with the Functional Independence Measure. *Australian Occupational Therapy Journal, 43*, 22-29.

GRADEpro GDT. (2025). *GRADEpro Guideline Development Tool [Software]*.

www.gradepro.org; McMaster University and Evidence Prime. https://www.gradepro.org/

Hawkins, R. J., Jowett, A., Godfrey, M., Mellish, K., Young, J., Farrin, A., Holloway,

I., Hewison, J., & Forster, A. (2017). Poststroke trajectories: The process of

recovery over the longer term following stroke. *Global Qualitative Nursing Research*, *4*, 2333393617730209. https://doi.org/10.1177/2333393617730209

Higgins, J. P. T., Thomas, J., Chandler, J., Cumpston, M., Li, T., Page, M. J., &

Welch, V. A. (Eds.). (2024). *Cochrane handbook for systematic reviews of*

*interventions* (Version 6.5). Cochrane. https://www.cochrane.org/handbook

Huber, S. K., Knols, R. H., Held, J. P. O., Betschart, M., Gartmann, S., Nauer, N., &

de Bruin, E. D. (2025). PEMOCS: effects of a concept-guided, PErsonalized, MOtor-Cognitive exergame training on cognitive functions and gait in chronic Stroke-a randomized, controlled trial. *Frontiers in Aging Neuroscience*, *17*, 1514594. https://doi.org/10.3389/fnagi.2025.1514594

Hung, J. W., Chou, C. X., Hsieh, Y. W., Wu, W. C., Yu, M. Y., Chen, P. C., Chang, H. F., & Ding, S. E. (2014). Randomized comparison trial of balance training

by using exergaming and conventional weight-shift therapy in patients with chronic stroke. *Archives of Physical Medicine and Rehabilitation*, *95*(9), 1629–1637. https://doi.org/10.1016/j.apmr.2014.04.029

Jandaghi, S., Tahan, N., Akbarzadeh Baghban, A., & Zoghi, M. (2021). Stroke

patients showed improvements in balance in response to visual restriction exercise. *Physical Therapy Research*, *24*(3), 211–217. https://doi.org/10.1298/ptr.E10081

Karamians, R., Proffitt, R., Kline, D., & Gauthier, L. V. (2020). Effectiveness of virtual

reality- and gaming-based interventions for upper extremity rehabilitation poststroke: A meta-analysis. *Archives of Physical Medicine and Rehabilitation*, *101*(5), 885–896. https://doi.org/10.1016/j.apmr.2019.10.195

Kilinc, S., Ali, C., Doganer, I., Yaksi, E., & Ozdemir, F. (2023). Effects of virtual

balance training and conservative rehabilitation on balance in chronic stroke patients. *Neurology Asia*, *28*(3). https://doi.org/10.54029/2023kcw

Kim, J. H. (2018). Effects of a virtual reality video game exercise program on upper

extremity function and daily living activities in stroke patients. *Journal of Physical Therapy Science*, *30*(12), 1408–1411. https://doi.org/10.1589/jpts.30.1408

Kiper, P., Godart, N., Cavalier, M., Berard, C., Cieślik, B., Federico, S., Kiper, A.,

Pellicciari, L., & Meroni, R. (2023). Effects of immersive virtual reality on

upper-extremity stroke rehabilitation: A systematic review with meta

analysis. *Journal of Clinical Medicine*, *13*(1), 146.

https://doi.org/10.3390/jcm13010146

Kuo, F.-L., Lee, H.-C., Kuo, T.-Y., Wu, Y.-S., Lee, Y.-S., Lin, J.-C., & Huang, S.-W.

(2023). Effects of a wearable sensor–based virtual reality game on upper

extremity function in patients with stroke. *Clinical Biomechanics*, *104*, 105944.

https://doi.org/10.1016/j.clinbiomech.2023.105944

Kwakkel, G., Stinear, C., Essers, B., Munoz-Novoa, M., Branscheidt, M., Cabanas

Valdés, R., ... & Verheyden, G. (2023). Motor rehabilitation after stroke: European Stroke Organisation (ESO) consensus-based definition and guiding framework. *European Stroke Journal*, *8*(4), 880-894.

https://doi.org/10.1177/23969873231191304

Laffont, I., Froger, J., Jourdan, C., Bakhti, K., van Dokkum, L. E. H., Gouaich, A.,

Bonnin, H. Y., Armingaud, P., Jaussent, A., Picot, M. C., Le Bars, E.,

Dupeyron, A., Arquizan, C., Gelis, A., & Mottet, D. (2020). Rehabilitation of

the upper arm early after stroke: Video games versus conventional

rehabilitation. A randomized controlled trial. *Annals of Physical and*

*Rehabilitation Medicine*, *63*(3). https://doi.org/10.1016/j.rehab.2019.10.009

Lam, S. S. L., Liu, T. W., Ng, S. S. M., Lai, C. W. K., & Woo, J. (2022). Bilateral

movement-based computer games improve sensorimotor functions in subacute stroke survivors. *Journal of Rehabilitation Medicine*, *54*, jrm00307. https://doi.org/10.2340/jrm.v54.913

Lam, S. S. L., Ng, S. S. M., Lai, C. W. K., & Woo, J. (2020). Bilateral movement

computer games to improve motor function of upper limb and quality of life in patients with sub-acute stroke: a randomised controlled trial: abridged secondary publication. *Hong Kong Medical Journal = Xianggang yi xue za zhi*, *26 Suppl 6*(6), 34–37.

Lang, C. E., Edwards, D. F., Birkenmeier, R. L., & Dromerick, A. W. (2008).

Estimating minimal clinically important differences of upper-extremity measures early after stroke. *Archives of Physical Medicine and Rehabilitation*, *89*(9), 1693-1700.

Lang, C. E., Macdonald, J. R., Reisman, D. S., Boyd, L., Jacobson Kimberley, T.,

Schindler Ivens, S. M., Hornby, T. G., Ross, S. A., & Scheets, P. L. (2009).

Observation of amounts of movement practice provided during stroke

rehabilitation. *Archives of Physical Medicine and Rehabilitation*, *90*(10), 1692

-1698. https://doi.org/10.1016/j.apmr.2009.04.005

Langhorne, P., Bernhardt, J., & Kwakkel, G. (2011). Stroke rehabilitation. *The*

*Lancet, 377*(9778), 1693-1702.

Latif, A. V., Hasan, R., Nikmatiyah, W. O. S., Waluyo, Y., Mubarak, H., & Zainuddin,

A. A. (2025). The effect of virtual reality-based exergaming on hand function and fine motor skills in post-stroke patients in a low-resource setting. *Fizjoterapia Polska*, (3), 193-202. https://doi.org/10.56984/8ZG7D19QDHS

Laver, K., George, S., Ratcliffe, J., Crotty, M. (2011). Virtual reality stroke

rehabilitation -hype or hope? *Australian Occupational Therapy Journal, 58*(3), 215-219. https://doi.org/10.1111/j.1440-1630.2010.00897.x

Lee, H. C., Huang, C. L., Ho, S. H., & Sung, W.H. (2017). The effect of a virtual

reality game intervention on balance for patients with stroke: a randomized

controlled trial. *Games for Health Journal*, *6*(5), 303–311.

https://doi.org/10.1089/g4h.2016.0109

Lee, S., Kim, Y., & Lee, B. H. (2016). Effect of virtual reality‐based bilateral upper

extremity training on upper extremity function after stroke: a randomized controlled clinical trial. *Occupational Therapy International*, *23*(4), 357-368.

Li, J., Yang, L., Lv, R., Kuang, J., Zhou, K., & Xu, M. (2023). Mediating effect of post

stroke depression between activities of daily living and health-related quality of life: meta-analytic structural equation modeling. *Quality of Life Research*, *32*(2), 331-338.

https://doi.org/10.1007/s11136-022-03225-9

Li, X., He, Y., Wang, D., & Rezaei, M. J. (2024). Stroke rehabilitation: from diagnosis

to therapy. *Frontiers in Neurology*, *15*, 1402729. https://doi.org/10.3389/fneur.2024.1402729

Lin, K. C., Fu, T., Wu, C. Y., Wang, Y. H., Liu, J. S., Hsieh, C. J., & Lin, S. F. (2010).

Minimal detectable change and clinically important difference of the Stroke Impact Scale in stroke patients. *Neurorehabilitation and Neural Repair*, *24*(5), 486–492. https://doi.org/10.1177/1545968309356295

Lohse, K. R., Hilderman, C. G. E., Cheung, K. L., Tatla, S., Van der Loos, H. F. M.

(2014). Virtual reality therapy for adults post-stroke: a systematic review and meta-analysis exploring virtual environments and commercial games in therapy. *PLoS ONE 9*(3), e93318. https://doi.org/10.1371/journal.pone.0093318

Long, Y., Ouyang, R. G., & Zhang, J. Q. (2020). Effects of virtual reality training on

occupational performance and self-efficacy of patients with stroke: a randomized controlled trial. *Journal of Neuroengineering and Rehabilitation*, *17*(1), 150. https://doi.org/10.1186/s12984-020-00783-2

Maier, M., Ballester, B. R., & Verschure, P. F. M. J. (2019). Principles of

neurorehabilitation after stroke based on motor learning and brain plasticity

mechanisms. *Frontiers in Systems Neuroscience*, *13*, 74.

https://doi.org/10.3389/fnsys.2019.00074

Marques-Sule, E., Arnal-Gómez, A., Buitrago-Jiménez, G., Suso-Martí, L., Cuenca

Martínez, F., & Espí-López, G. V. (2021). Effectiveness of Nintendo Wii and physical therapy in functionality, balance, and daily activities in chronic stroke patients. *Journal of the American Medical Directors Association*, *22*(5), 1073-1080. https://doi.org/10.1016/j.jamda.2021.01.076

Mazher, Y., Shafee, I., & Dar, H. (2025). Effects of Exer gaming on upper extremity

function and activities of daily living in sub-acute stroke patient (Randomized Controlled Trial). *Foundation University Journal of Rehabilitation Sciences*, *5*(1), 34-41.

Medeiros, G. C., Roy, D., Kontos, N., & Beach, S. R. (2020). Post-stroke depression:

a 2020 updated review. *General Hospital Psychiatry*, *66*, 70-80.

https://doi.org/10.1016/j.genhosppsych.2020.06.011

Michie, S., Johnston, M., Francis, J., Hardeman, W., & Eccles, M. (2008). From

theory to intervention: mapping theoretically derived behavioural determinants to behaviour change techniques. *Applied Psychology*, *57*(4), 660–680.

https://doi-org.libproxy1.nus.edu.sg/10.1111/j.1464-0597.2008.00341.x

Miller, K. K., Porter, R. E., DeBraun-Sprague, E., Van Puymbroeck, M., Schmid, A.

A. (2016). Exercise after stroke: patient adherence and beliefs after discharge from rehabilitation. *Topics in Stroke Rehabilitation, 24*(2), 142-148.

https://doi.org/10.1080/10749357.2016.1200292

Nilsen, P. (2015). Making sense of implementation theories, models and

frameworks. *Implementation Science*, 10, 53 (2015). https://doi.org/10.1186/s13012-015-0242-0

Norouzi-Gheidari, N., Archambault, P. S., Monte-Silva, K., Kairy, D., Sveistrup, H.,

Trivino, M., Levin, M. F., & Milot, M. H. (2021). Feasibility and preliminary efficacy of a combined virtual reality, robotics and electrical stimulation intervention in upper extremity stroke rehabilitation. *Journal of Neuroengineering and Rehabilitation*, *18*(1), 61. https://doi.org/10.1186/s12984-021-00851-1

O’Flaherty, D., & Ali, K. (2024). Recommendations for upper limb motor recovery:

an overview of the UK and European rehabilitation after stroke guidelines (2023). *Healthcare*, *12*(14), 1433. https://doi.org/10.3390/healthcare12141433

Oh, Y. B., Kim, G. W., Han, K. S., Won, Y. H., Park, S. H., Seo, J. H., & Ko, M. H.

(2019). Efficacy of virtual reality combined with real instrument training for patients with stroke: a randomized controlled trial. *Archives of Physical Medicine and Rehabilitation*, *100*(8), 1400-1408.

https://doi.org/10.1016/j.apmr.2019.03.013

Ozen, S., Senlikci, H. B., Guzel, S., & Yemisci, O. U. (2021). Computer game

assisted task specific exercises in the treatment of motor and cognitive function and quality of life in stroke: A randomized control study. *Journal of Stroke and Cerebrovascular Diseases: The Official Journal of National Stroke Association*, *30*(9), 105991. https://doi.org/10.1016/j.jstrokecerebrovasdis.2021.105991

Page, S. J., Fulk, G. D., & Boyne, P. (2012). Clinically important differences for the

upper-extremity Fugl-Meyer scale in people with minimal to moderate impairment due to chronic stroke. *Physical Therapy*, *92*(6), 791–798.

Park, M., & Ha, Y. (2023). Effects of virtual reality-based cognitive rehabilitation in

stroke patients: a randomized controlled trial. *Healthcare*, *11*(21), 2846. https://doi.org/10.3390/healthcare11212846

Park, Y.-S., An, C.-S., & Lim, C.-G. (2021). Effects of a rehabilitation program using

a wearable device on the upper limb function, performance of activities of daily living, and rehabilitation participation in patients with acute stroke. *International Journal of Environmental Research and Public Health*, *18*(11), 5524. https://doi.org/10.3390/ijerph18115524

Pollock, A., Farmer, S. E., Brady, M. C., Langhorne, P., Mead, G. E., Mehrholz, J., &

van Wijck, F. (2014). Interventions for improving upper limb function after stroke. *Cochrane Database of Systematic Reviews,* (11), CD010820.

https://doi.org/10.1002/14651858.CD010820.pub2

Proffitt, R., & Lange, B. (2015). Considerations in the efficacy and effectiveness of

virtual reality interventions for stroke rehabilitation: moving the field forward. *Physical Therapy*, *95*(3), 441–448. https://doi.org/10.2522/ptj.20130571

Puderbaugh, M., & Emmady, P. D. (2023). Neuroplasticity. In *StatPearls*. StatPearls

Publishing.

Rajsic, S., Gothe, H., Borba, H. H., Sroczynski, G., Vujicic, J., Toell, T., & Siebert, U.

(2019). Economic burden of stroke: a systematic review on post-stroke care. *The European Journal of Health Economics : HEPAC: Health Economics in Prevention and Care*, *20*(1), 107–134. https://doi.org/10.1007/s10198-018-0984-0

Rémy-Néris, O., Le Jeannic, A., Dion, A., Médée, B., Nowak, E., Poiroux, É., &

Durand Zaleski, I. (2021). Additional, mechanized upper limb self-rehabilitation in patients with subacute stroke: The REM-AVC randomized trial. *Stroke*, *52*(6), 1938–1947. https://doi.org/10.1161/STROKEAHA.120.032545

Review Manager (RevMan). Version 8.13.0. The Cochrane Collaboration,

(December 18, 2024). https://revman.cochrane.org/info

Rodríguez-Hernández, M., Criado-Álvarez, J. J., Corregidor-Sánchez, A. I., Martín

Conty, J. L., Mohedano-Moriano, A., & Polonio-López, B. (2021). Effects of virtual reality-based therapy on quality of life of patients with subacute stroke: a three-month follow-up randomized controlled trial. *International Journal of Environmental Research and Public Health*, *18*(6), 2810. https://doi.org/10.3390/ijerph18062810

Saeedi, S., Ghazisaeedi, M., & Rezayi, S. (2021). Applying game-based approaches

for physical rehabilitation of poststroke patients: A systematic review. *Journal of Healthcare Engineering*, *2021*, 9928509. https://doi.org/10.1155/2021/9928509

Salatino, A., Zavattaro, C., Gammeri, R., Cirillo, E., Piatti, M. L., Pyasik, M., Serra,

H., Pia, L., Geminiani, G., & Ricci, R. (2023). Virtual reality rehabilitation for unilateral spatial neglect: A systematic review of immersive, semi-immersive and non-immersive techniques. *Neuroscience and biobehavioral reviews*, *152*, 105248. https://doi.org/10.1016/j.neubiorev.2023.105248

Salbach, N. M., Mountain, A., Lindsay, M. P., Blacquiere, D., McGuff, R., Foley, N.,

Corriveau, H., Fung, J., Gierman, N., Inness, E., Linkewich, E., O'Connell, C., Sakakibara, B., Smith, E. E., Tang, A., Timpson, D., Vallentin, T., White, K., Yao, J., & Canadian Stroke Best Practice Recommendations Advisory Committee, in collaboration with the Canadian Stroke Consortium and the Canadian Partnership for Stroke Recovery (2022). Canadian Stroke Best Practice Recommendations: Virtual Stroke Rehabilitation Interim Consensus Statement 2022. *American Journal of Physical Medicine & Rehabilitation*, *101*(11), 1076–1082. https://doi.org/10.1097/PHM.0000000000002062

Sanchez-Gil, J. J., Saez-Manzano, A., Lopez-Luque, R., Ochoa-Sepulveda, J. J., &

Canete-Carmona, E. (2025). Gamified devices for stroke rehabilitation: A systematic review. *Computer Methods and Programs in Biomedicine*, *258*, 108476. https://doi.org/10.1016/j.cmpb.2024.108476

Shin, J. H., Bog Park, S., & Ho Jang, S. (2015). Effects of game-based virtual reality

on health-related quality of life in chronic stroke patients: A randomized, controlled study. *Computers in Biology and Medicine*, *63*, 92–98. https://doi.org/10.1016/j.compbiomed.2015.03.011

Shin, J. H., Kim, M. Y., Lee, J. Y., Jeon, Y. J., Kim, S., Lee, S., Seo, B., & Choi, Y. (2016). Effects of virtual reality-based rehabilitation on distal upper extremity

function and health-related quality of life: a single-blinded, randomized controlled trial. *Journal of NeuroEngineering and Rehabilitation*, *13*(1). https://doi.org/10.1186/s12984-016-0125-x

Sidani, S., & Braden, C. J. (2011). Design, evaluation, and translation of nursing

interventions. Ames, IA: Wiley-Blackwell.

Şimşek, T. T., & Çekok, K. (2016). The effects of Nintendo Wii(TM)-based balance

and upper extremity training on activities of daily living and quality of life in

patients with sub-acute stroke: a randomized controlled study. *The International Journal of Neuroscience, 126*(12), 1061–1070. https://doi.org/10.3109/00207454.2015.1115993

Soleimani, M., Ghazisaeedi, M., & Heydari, S. (2024). The efficacy of virtual reality

for upper limb rehabilitation in stroke patients: a systematic review and meta-analysis. *BMC Medical Informatics and Decision Making*, *24*(1), 135. https://doi.org/10.1186/s12911-024-02534-y

Song, Y. H., & Lee, H. M. (2021). Effect of Immersive Virtual Reality-Based Bilateral

Arm Training in Patients with Chronic Stroke. *Brain Sciences*, *11*(8), 1032. https://doi.org/10.3390/brainsci11081032

Su, J. J., Wong, A. K. C., He, X. F., Zhang, L. P., Cheng, J., Lu, L. J., Lan, L., Wang,

Z., Lin, R. S. Y., & Batalik, L. (2024). Feasibility and effectiveness of cardiac telerehabilitation for older adults with coronary heart disease: A pilot randomized controlled trial. *Contemporary Clinical Trials Communications*, *42*, 101365. https://doi.org/10.1016/j.conctc.2024.101365

Stinear, C. M., Lang, C. E., Zeiler, S., & Byblow, W. D. (2020). Advances and

challenges in stroke rehabilitation. *The Lancet. Neurology*, *19*(4), 348–360. https://doi.org/10.1016/S1474-4422(19)30415-6

Térémetz, M., García, A. A., Hanneton, S., Roby-Brami, A., Roche, N., Bensmail, D.,

Lindberg, P. G., & Robertson, J. (2022). Improving upper-limb and trunk kinematics by interactive gaming in individuals with chronic stroke: A single-blinded RCT. *Annals of Physical and Rehabilitation Medicine, 65*(3), 101622–101622. https://doi.org/10.1016/j.rehab.2021.101622

Tosto-Mancuso, J., Tabacof, L., Herrera, J. E., Breyman, E., Dewil, S., Cortes, M.,

Correa Esnard, L., Kellner, C. P., Dangayach, N., & Putrino, D. (2022). Gamified neurorehabilitation strategies for post-stroke motor recovery: challenges and advantages. *Current Neurology and Neuroscience Reports*, *22*(3), 183–195. https://doi.org/10.1007/s11910-022-01181-y

Vázquez-Guimaraens, M., Caamaño-Ponte, J. L., Seoane-Pillado, T., & Cudeiro, J.

(2021). Factors related to greater functional recovery after suffering a

stroke. *Brain Sciences*, *11*(6), 802. https://doi.org/10.3390/brainsci11060802

Van der Lee, J. H., De Groot, V., Beckerman, H., Wagenaar, R. C., Lankhorst, G. J.,

& Bouter, L. M. (2001). The intra-and interrater reliability of the action research arm test: a practical test of upper extremity function in patients with stroke. *Archives of Physical Medicine and Rehabilitation*, *82*(1), 14-19.

Velmurugan, G., Viswanath, S., Andrews Milton, J. (2023). Effectiveness of virtual

reality training on upper limb motor function in stroke patients: a randomized

control trial. Indian Journal of Physiotherapy and Occupational Therapy,

17(3), 60-66.

Wang, L., Chen, J.-L., Wong, A. M. K., Liang, K.-C., & Tseng, K. C. (2022). Game

based virtual reality system for upper limb rehabilitation after stroke in a clinical environment: systematic review and meta-analysis. *Games for Health*, *11*(5), 277–297.

Winstein, C. J., Stein, J., Arena, R., Bates, B., Cherney, L. R., Cramer, S. C.,

Deruyter, F., Eng, J. J., Fisher, B., Harvey, R. L., Lang, C. E., MacKay-Lyons, M., Ottenbacher, K. J., Pugh, S., Reeves, M. J., Richards, L. G., Stiers, W., Zorowitz, R. D., & American Heart Association Stroke Council, Council on Cardiovascular and Stroke Nursing, Council on Clinical Cardiology, and Council on Quality of Care and Outcomes Research (2016). Guidelines for Adult Stroke Rehabilitation and Recovery: A Guideline for Healthcare Professionals From the American Heart Association/American Stroke Association. *Stroke*, *47*(6), e98–e169. https://doi.org/10.1161/STR.0000000000000098

World Stroke Organization. (2024). *Impact of Stroke*. World Stroke Organization. https://www.world-stroke.org/world-stroke-day-campaign/about-stroke/impact- of-stroke

Wondergem, R., Pisters, M. F., Wouters, E. J., Olthof, N., de Bie, R. A., Visser-Meily,

J. M., & Veenhof, C. (2017). The course of activities in daily living: who is at

risk for decline after first ever stroke?. *Cerebrovascular Diseases*, *43*(1-2), 1-8. https://doi.org/10.1159/000451034

Wu, J., Zeng, A., Chen, Z., Wei, Y., Huang, K., Chen, J., & Ren, Z. (2021). Effects of

virtual reality training on upper limb function and balance in stroke patients: systematic review and meta-meta-analysis. *Journal of Medical Internet Research*, *23*(10), e31051. https://doi.org/10.2196/31051

Yousufuddin, M., & Young, N. (2019). Aging and ischemic stroke. *Aging*, *11*(9),

2542–2544. https://doi.org/10.18632/aging.101931
